# Are Terminal Alkynes Necessary for MAO-A/MAO-B Inhibition? A New Scaffold Is Revealed

**DOI:** 10.3390/molecules29112486

**Published:** 2024-05-24

**Authors:** Panagiou Mavroeidi, Leandros P. Zorba, Nikolaos V. Tzouras, Stavros P. Neofotistos, Nikitas Georgiou, Kader Sahin, Murat Şentürk, Serdar Durdagi, Georgios C. Vougioukalakis, Thomas Mavromoustakos

**Affiliations:** 1 Laboratory of Organic Chemistry, Department of Chemistry, National and Kapodistrian University of Athens, 15771 Athens, Greece; gmavroeidi@hotmail.com (P.M.); leozor@chem.uoa.gr (L.P.Z.); nicktzouras@chem.uoa.gr (N.V.T.); stavrosneo94@gmail.com (S.P.N.); nikitage@chem.uoa.gr (N.G.); 2 Department of Analytical Chemistry, School of Pharmacy, Bahcesehir University, 34349 Istanbul, Turkey; serdardurdagi@gmail.com; 3 Department of Biochemistry, Faculty of Pharmacy, Agri Ibrahim Cecen University, 04100 Agri, Turkey; murat.senturk@istanbul.edu.tr; 4 Molecular Therapy Laboratory, Department of Pharmaceutical Chemistry, School of Pharmacy, Bahcesehir University, 34349 Istanbul, Turkey; 5 Computational Biology and Molecular Simulations Laboratory, Department of Biophysics, School of Medicine, Bahcesehir University, 34349 Istanbul, Turkey; 6 Laboratory for Innovative Drugs (Lab4IND), Computational Drug Design Center (HİTMER), Bahcesehir University, 34349 Istanbul, Turkey

**Keywords:** propargylamines, molecular dynamics, MM/GBSA, monoaminoxidases

## Abstract

A versatile family of quaternary propargylamines was synthesized employing the KA^2^ multicomponent reaction, through the single-step coupling of a number of amines, ketones, and terminal alkynes. Sustainable synthetic procedures using transition metal catalysts were employed in all cases. The inhibitory activity of these molecules was evaluated against human monoaminoxidase (hMAO)-A and hMAO-B enzymes and was found to be significant. The IC_50_ values for hMAO-B range from 152.1 to 164.7 nM while the IC_50_ values for hMAO-A range from 765.6 to 861.6 nM. Furthermore, these compounds comply with Lipinski’s rule of five and exhibit no predicted toxicity. To understand their binding properties with the two target enzymes, key interactions were studied using molecular docking, all-atom molecular dynamics (MD) simulations, and MM/GBSA binding free energy calculations. Overall, herein, the reported family of propargylamines exhibits promise as potential treatments for neurodegenerative disorders, such as Parkinson’s disease. Interestingly, this is the first time a propargylamine scaffold bearing an internal alkyne has been reported to show activity against monoaminoxidases.

## 1. Introduction

The design and development of synthetic protocols leading to the preparation of compounds with biological activity is a highly important goal of synthetic organic chemistry. This fact is reflected by the vast number of patents and related research articles published every year. Propargylamines have gained significant attention over recent decades, due to their unique structural characteristics, which, among others, allow for further functionalization [1]. The propargylamine core is found in compounds of great pharmaceutical interest, such as pargyline and selegiline, which are used to treat neurodegenerative diseases (NDs), such as depression, as well as Parkinson’s disease (PD) and Alzheimer’s disease (AD) [1,2,3,4].

Among others, propargylamine-based compounds act as potential inhibitors of the monoaminoxidase (MAO) enzymes, which are located at the superficial membrane of mitochondria, being responsible for the oxidative deamination of a variety of important flavin-binding neurotransmitters, such as dopamine, adrenaline, noradrenaline, tyramine, and serotonin [2,5,6,7,8]. In patients diagnosed with neurodegenerative disorders, an excessive breakdown of neurotransmitters by the MAO-B enzyme is observed. This excessive breakdown leads to a significant decrease in the concentration of neurotransmitters in the synaptic cleft. The reduction in the neurotransmitter concentration causes various problems associated with these neurodegenerative disorders, including the emergence of related symptoms, neuronal cell apoptosis, and the formation of harmful neurotoxic by-products, such as hydrogen peroxide, ammonia, and aldehydes [2,5,6,7,8]. Furthermore, MAO activation contributes to the formation of neurofibrillary tangles and the destruction of cholinergic neurons [5,6,7,8]. MAO inhibitors are pharmaceutical compounds providing benefits in the treatment of various neurodegenerative disorders. They also exhibit anti-inflammatory effects by inhibiting the degradation of biogenic amines and increasing catecholamine levels in immune and non-immune cells [8,9,10]. In addition to their role as MAO-B inhibitors, efforts have been made to develop propargylamine-based drugs with multitargeted effects. This approach aims to achieve simultaneous inhibition at different sites related to the underlying causes of neurodegenerative diseases [2,3]. A major challenge is the development of more potent selective MAO inhibitors [4]. Although the crystal structures of MAO-A and MAO-B isoforms share similarities, their mechanisms of action, tissue distribution, and regions involved in substrate–inhibitor identification are different [5,11]. Nevertheless, their solved structures allow for the application of drug–target interactions at the molecular level and the design of structure-based rational drug therapies.

As mentioned above, the propargylamine scaffold can be constructed in several ways, providing access to compounds with high synthetic value, including allenes and heterocycles such as pyridines, oxazoles, thiazoles, indoles, and triazoles [1,12,13]. The synthesis of these molecules can be facilitated due to the ability of the nitrogen atom of the propargylamine unit to act as a nucleophile. On the other hand, the alkyne moiety can play many chemical roles, depending on the presence of additional functional groups in the propargylamine compound of interest. For example, one approach towards pyrrolines [14] or pyrrolidines [15] involves the utilization of a double or a triple bond near the nitrogen atom or in a separate compound [16,17,18,19]. For oxazoles, the presence of a carbonyl group adjacent to the amine group is crucial for initiating the cyclization process [20,21]. Conversely, in oxazolidinones, the nitrogen atom initiates a nucleophilic attack on carbon dioxide, followed by subsequent nucleophilic attack by oxygen on the nearest sp-carbon atom of the triple bond. This sequence results in the formation of a five-membered heterocycle [22].

The synthesis of propargylamines can be accomplished by pre-forming the imine intermediates, followed by nucleophilic attack by acetylides, which can in turn be prepared in situ by the use of strong bases, or, even better, by employing transition metal catalysts [1]. Today, the most efficient route towards these compounds is the A^3^ coupling reaction, a multicomponent reaction involving the coupling of an amine, an aldehyde, and a terminal alkyne, as well as a transition state metal source as a catalyst [1,12]. Multicomponent reactions provide easy access to these valuable synthons under only one step, effectively contributing to atom and step economy. Additionally, the reactants used are, in most cases, simple and commercially available starting materials, whereas the catalysts used in the majority of cases are based on non-toxic, abundant, low-cost metal sources, such as Cu and Zn. The rate-limiting step of this transformation is the nucleophilic attack of the in situ-generated metal acetylide to the aldimine intermediate, also formed over the course of the reaction. When ketones are employed as carbonyl reagents, the corresponding reaction (KA^2^ coupling) is more challenging, given that the ketimine intermediate is much less reactive, both electronically and stereochemically, than its aldimine counterpart [23]. This is reflected by the relatively limited number of synthetic protocols based on this transformation, employing Au, Zn, or (mainly) Cu as metal sources [24,25,26,27,28,29,30]. Herein, we report the biological evaluation and molecular modeling studies of a series of tetrasubstituted propargylamine derivatives synthesized previously by our group for their ability to act as efficient MAO inhibitors.

## 2. Results

### 2.1. Synthesis of Propargylamines

**Scheme 1:**
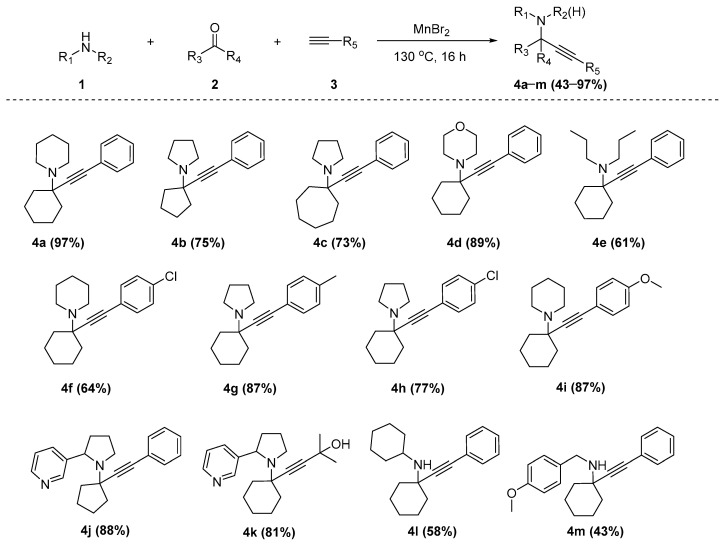
Synthesis of propargylamines **4a**–**4m** under MnBr_2_ catalysis.

The synthesis of the herein studied quaternary propargylamines was achieved by employing two previously reported sustainable protocols based on Mn or Zn catalytic systems [27,28]. More specifically, propargylamines **4a**–**4m** were synthesized via a multicomponent reaction between a variety of cyclic and linear alkyl ketones, a range of amines, and a number of aryl- or alkyl-substituted alkynes, under Mn catalysis (Scheme 1), in 43–97% isolated yields [28].

**Scheme 2:**
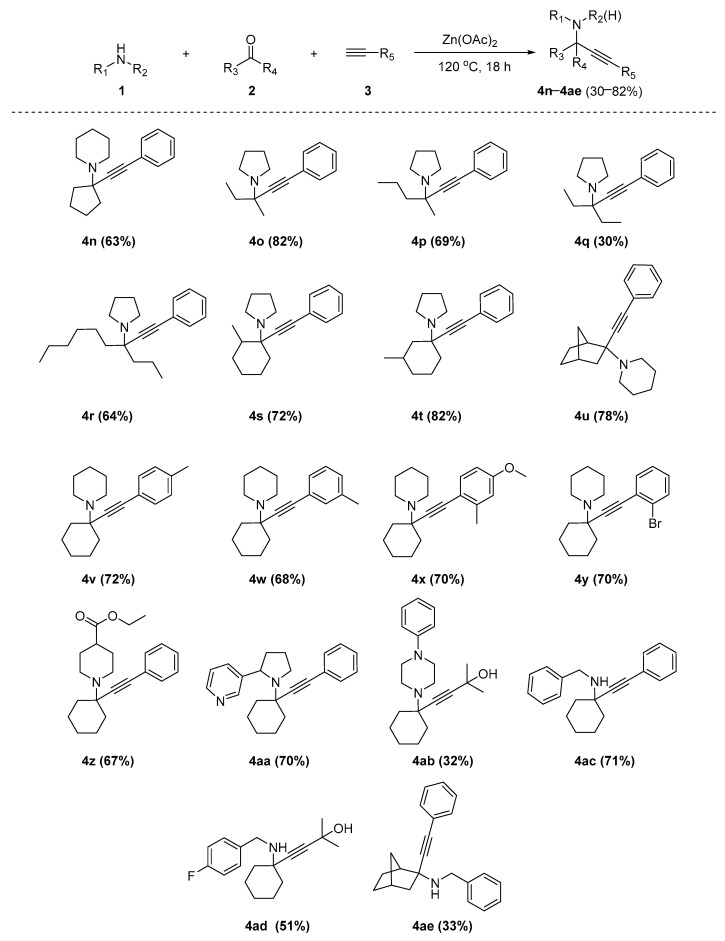
Synthesis of propargylamines **4n**–**4ae**, catalyzed by Zn(OAc)_2_.

Propargylamines **4n**–**4ae** were synthesized according to our Zn-based catalytic protocol (Scheme 2), employing a variety of amines, linear or cyclic alkyl ketones, and a variety of terminal alkynes, allowing for the isolation of the desired products in a 30–82% isolated yield [27].

### 2.2. In Vitro Biological Activity Assays

All the studied propargylamines demonstrated sub-micromolar inhibition of the hMAO-B enzyme. Among them, the most active compounds were also evaluated with hMAO-A (Table 1). In a study conducted by Ramsay et al., the IC_50_ value for pargyline as a MAO-B template was 2.25 μM, which served as the basis for the derivatization of the compounds and is known for its potent MAO inhibitory properties [31]. In contrast, the IC_50_ value for MAO-A was determined to be 118 μM. Notably, all the propargylamines synthesized and studied herein exhibit substantially lower inhibition values (ranging from 152.1 to 345.9 nM) for MAO-B, suggesting that these compounds are more effective inhibitors compared to pargyline. Among the propargylamines tested, **4k** and **4ad** showed the most significant efficacy against both MAO-A and MAO-B isoforms. This enhanced effectiveness can be attributed to their 2-methylbut-3-yn-2-ol moiety. However, propargylamine **4ab**, which also contains the same moiety, exhibits a slightly higher IC_50_ value than **4k** and **4ad**. This disparity can be attributed to the steric hindrance of the bulky groups situated near this specific group in its structure. On the other hand, molecule **4aa** demonstrates the weakest inhibition of both enzymes. Considering the selectivity index (SI) values, it becomes evident that molecule **4k** displays the most favorable and effective results.

**Scheme 3:**
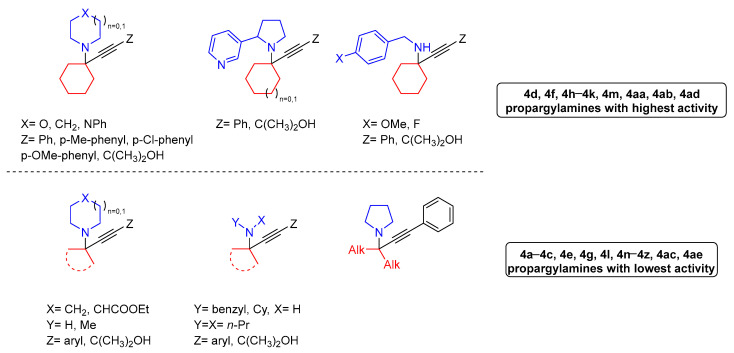
Classified synthetic compounds according to their activity.

From Table 1, the synthetic compounds can be classified into two major groups of molecules: those with the highest activity against the two enzymes and those with the lowest activity. Interestingly, the major modification that differentiates the two groups is the cyclopentene or cyclohexene ring existing only on the active compounds. The inactive ones contain open-chain smaller alkyl groups or cyclopropane. Thus, this structural hydrophobic part of the molecules appears to be essential for their activity. This will be illustrated below with the molecular modeling results (Scheme 3).

### 2.3. Molecular Modeling Results

#### 2.3.1. Molecular Docking and MD Simulations

Based on the in vitro biological activity results, we selected the six promising compounds (**4k**, **4j**, **4m**, **4ad**, **4aa**, and **4ab**) and employed Induced Fit Docking (IFD) calculations to evaluate their binding properties to the hMAO-A and hMAO-B isoforms. The IFD method is a widely used computational approach for predicting the binding modes of ligands to proteins, allowing for the simultaneous optimization of both the ligand and the protein structure in the docking process. This method takes into account the flexibility of the binding pocket residues, thus allowing for a more accurate prediction of the ligand binding affinity and the binding mode. The evaluation of the binding properties of the compounds can provide insights into their potential therapeutic applications. By comprehending the molecular-level interactions between compounds and enzymes, novel drug candidates can be pinpointed or the effectiveness of current ones can be enhanced. The protein preparation module was used to optimize the structures of hMAO-A and hMAO-B for the docking studies. The OPLS3e force field was performed to optimize the protein structure. The LigPrep module of the Maestro molecular modeling suite was used to prepare the ligands for docking studies. LigPrep can generate a variety of protonation states and tautomers for the compounds, and then it can calculate the low-energy conformers of the compounds. This ensures that the compounds are in a biochemically relevant state and that they can interact effectively with the protein. Overall, optimizing the protein and ligands before docking studies is a critical step to ensure accurate and reliable results. Ligands that selectively bind to hMAO-B over hMAO-A are of particular interest, because they can provide therapeutic benefits, while minimizing unwanted side effects. The high sequence identity between hMAO-A and hMAO-B targets can make it challenging to develop compounds with high selectivity for one over the other. In fact, there are significant differences between the active sites of hMAO-A and hMAO-B, which can be exploited to develop selective inhibitors. For example, the active site of hMAO-B is more hydrophobic than that of hMAO-A, which can be targeted by compounds with specific hydrophobic interactions. One such ligand is selegiline, which is an irreversible inhibitor of hMAO-B. Selegiline has been shown to have a higher selectivity for hMAO-B over hMAO-A, which is thought to contribute to its therapeutic efficacy in the treatment of Parkinson’s disease [32].

Tyr326 and Tyr398 are two key amino acid residues [33,34] in the active site of hMAO-B, and, therefore, in the molecular docking and MD simulations, we mainly observed their interactions with the selected ligands. For instance, **4k** binds to a considerably hydrophobic pocket and predominantly interacts with Pro104, Phe168, Leu171, Cys172, Ile199, Ile316, Tyr326, Tyr398, and Leu435. Additionally, Tyr398 and Leu171 participate in interactions through hydrogen bonds. These interactions help stabilize the binding of the ligands to the enzyme and are important for the catalytic activity of hMAO-B. The gatekeeper residues of hMAO-B are commonly recognized as Leu171, Ile199, and Tyr326 [35]. These amino acids are strategically located in the substrate binding pocket of hMAO-B, and are responsible for controlling the entry and exit of substrates and inhibitors. Specifically, Leu171 and Ile199 form a hydrophobic barrier that restricts the size and shape of the substrate binding pocket, while Tyr326 is involved in substrate recognition and binding through hydrogen bonding and π-stacking interactions. The substrate cavity has an ellipsoidal disk shape, and it is lined by specific amino acid residues on either side. Leu171, Cys172, and Tyr398 are on one side of the ellipsoidal disk-shaped cavity, while Ile198, Ile199, and Tyr435 are on the opposite side. The inhibitors that bind to this cavity are referred to as bound inhibitors. The specific interactions between the inhibitors and the amino acid residues in the substrate cavity may play an essential role in determining the binding affinity and specificity of the inhibitors [36].

Tyr188 is involved in the cavity, and it has a side chain that likely contributes to the shape and properties of the cavity. Additionally, the aromatic residues Tyr60, Tyr326, and Phe343 are involved in the cavity, possibly through their interactions with each other and other amino acid residues [36]. The formation of this cavity may have functional significance in the context of the protein in which it occurs. The specific shape and properties of the cavity can determine which molecules can bind to it and how tightly they bind, influencing the function and activity of the protein.

After docking, the selected compounds, which are presumably potential hit compounds, were subjected to all-atom MD simulations to assess their stability and dynamic behavior. MD simulation is a powerful computational technique used to study the behavior of atoms and molecules in a system over time. We performed MD simulations on the complexes of selected compounds with the active site of hMAO-A and hMAO-B as the initial conformations. The simulations were conducted over a period of 100 ns and an explicit solvent environment was used.

Root mean square deviation (RMSD) was used to quantify the structural fluctuations of a protein or a protein–ligand complex over the course of the MD simulations. In particular, RMSD is often used to monitor the stability of the protein backbone or a specific part of the protein, such as the ligand binding site. In general, a low RMSD value indicates that the protein or protein–ligand complex is relatively stable and has a well-defined structure, while a high RMSD value indicates that the protein or complex is undergoing significant structural fluctuations or is less stable. Figure S1 shows the time-dependent RMSD of selected ligand–protein complexes during the MD simulations. The results suggest that all of the ligand–protein complexes reached equilibrium status after an initial period of fluctuations and then remained stable for the rest of the simulation, except for the **4ab** ligand–protein complex. This implies that **4ab** may have induced some significant structural changes or instability in the protein–ligand complex during the simulations, which could have important implications for its binding affinity, activity, or selectivity.

Throughout the simulation, the RMSD fluctuations were consistently below a threshold of approximately 4 Å. This finding implies that the ligand–protein complexes did not undergo substantial conformational changes or alterations in their overall structural properties. The compounds exhibited the ability to fold into a stable state and remained in equilibrium with the protein throughout the simulations, suggesting that they maintained their binding interactions without causing significant structural perturbations. This desirable property makes these compounds promising hits, as it indicates their potential to interact with the target protein in a stable and effective manner, without compromising its structural stability or function.

The RMSD graphs reveal that among the hit compounds, **4j** and **4k** demonstrate lower fluctuations in RMSD compared to the rest. This observation implies that **4j** and **4k** can be more stable and exhibit more consistent binding interactions with the target protein than the other hit compounds. The root mean square fluctuation (RMSF) serves as a metric to assess the average deviation of individual atoms or residues within a protein structure from their average positions over MD simulations or other structural analysis. To identify regions of the protein that exhibit varying degrees of flexibility or dynamism, the RMSF values were plotted against the corresponding residue numbers, as illustrated in Figure S2. Regions characterized by higher RMSF values are visually represented as peaks in the graph. These peaks might indicate areas in the protein that possess greater flexibility, influenced by their location within the protein’s structure or their interactions with other molecules or solvent molecules in the simulation environment. It is crucial to recognize that higher RMSF values do not necessarily indicate disorder or instability in a protein region; rather, they suggest that those regions might display more freedom of movement or flexibility compared to other parts of the protein. Moreover, fluctuations in the RMSF values between different conformations or states of the protein can offer insights into conformational changes or other dynamic processes that may be significant for the protein’s function. RMSF provides an understanding of the average deviation of each atom in a protein over time. It is a valuable tool to analyze the flexibility and dynamic behavior of proteins, with higher RMSF values indicating more flexible and dynamic regions, while lower RMSF values correspond to more stable and rigid regions. Specifically, the residues numbered between 500 and 600 display increased fluctuations in the presence of all the selected compounds.

#### 2.3.2. Interactions between Selected Hit Compounds and the Target Protein

**Figure 1:**
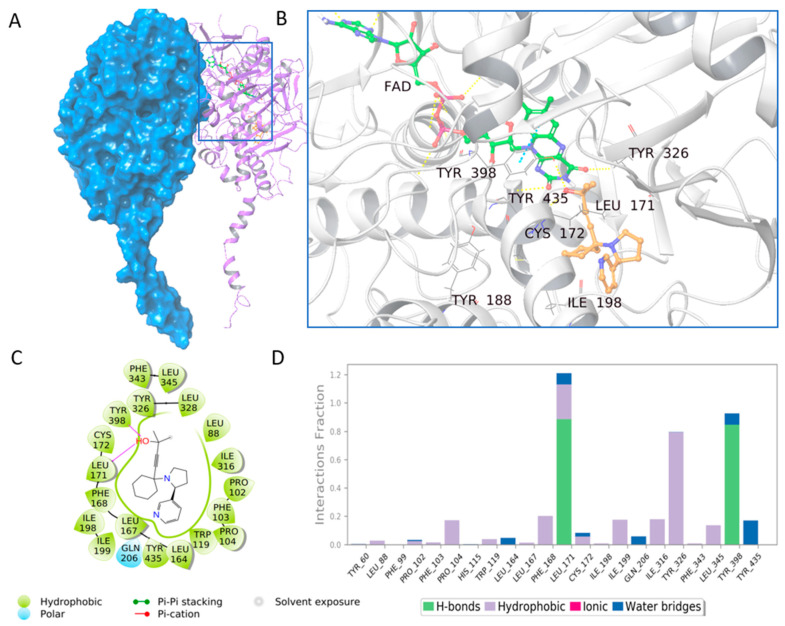
(**A**) Representation of the binding mode of **4k** with hMAO-B. (**B**) Three-dimensional ligand interactions of **4k** at the binding pocket of hMAO-B. The protein is depicted as ribbons, and the compound is shown as sticks. (**C**) Two-dimensional critical ligand interactions with amino acid residues of hMAO-B. The various types of interactions are represented in colors designated under the conformation. This conformation represents the final state of production phase after performing MD simulations. (**D**) Interaction fractions of **4k** throughout 100 ns MD simulations in the binding pocket of hMAO-B.

The 3D structure of a ligand provides information about the bioactive conformation of the ligand in the binding site, which can be important for optimizing the structure of the ligand to improve its binding affinity and selectivity. **4k** was found to exhibit the highest binding affinity with hMAO-B (152.1 nM) among the six selected compounds. Figure 1 displays the 3D binding mode and 2D ligand interaction graphs of **4k**. **4k** showed crucial hydrogen bonding interactions with the Leu171 and Tyr398 residues of hMAO-B and these hydrogen bond interactions were mostly conserved throughout the MD simulations, suggesting that these interactions are important for the binding affinity of **4k** with hMAO-B. Hydrophobic interactions occur between nonpolar regions of molecules and are important for stabilizing protein–ligand complexes in hydrophobic environments. The hydrophobic interactions between **4k** and some of the important amino acid residues such as Phe168, Leu171, Ile199, and Tyr326 contributed to the ligands’ additional stability in the binding site. The stability of the protein–ligand complex is an important factor in determining the overall efficacy of the ligand as a drug candidate. Hydrogen bonds and water bridges are important factors that contribute to the stability of ligand binding to a protein. The stable profile of **4k** at the binding site of hMAO-B can indeed be explained by the high number of hydrogen bonds and water bridges established by the ligand (residues Leu171, Tyr398, and Tyr435). The combination of hydrogen bonds and water bridges stabilizes the interaction between **4k** and hMAO-B, making it energetically favorable and leading to a stable binding profile.

**Figure 2:**
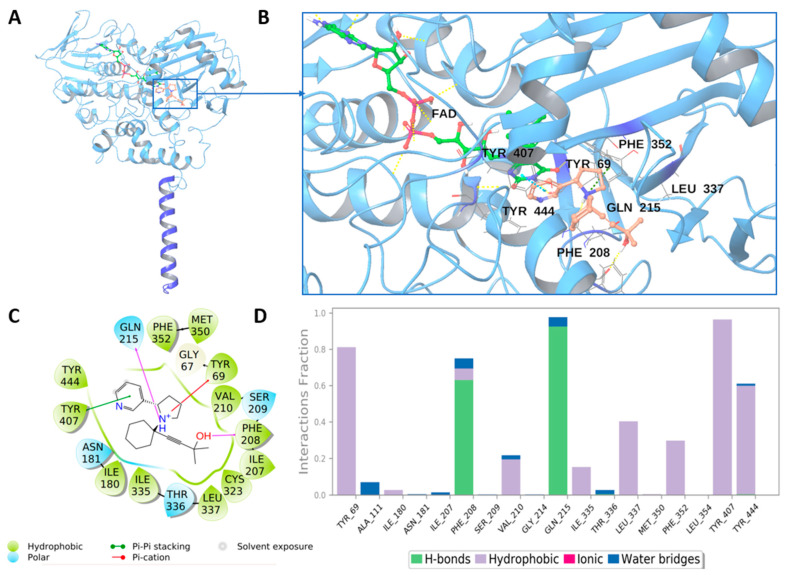
(**A**) Representation of the binding mode of **4k** with hMAO-A. (**B**) Three-dimensional ligand interactions of **4k** at the binding pocket of hMAO-A. The protein is depicted as ribbons, and the compound is shown as sticks. (**C**) Two-dimensional critical ligand interactions with amino acid residues of hMAO-B. The various types of interactions are represented in colors designated under the pose. This pose represents the final state of production phase after performing MD. (**D**) Interaction fractions of **4k** throughout 100 ns MD simulations in the binding pocket of hMAO-A.

The inhibiting capacity of the selected six compounds against hMAO-A was also evaluated. A potential drug candidate, **4k** interacts with specific residues through both hydrophobic and hydrogen bonding interactions within the binding pocket of hMAO-A. The hydrophobic amino acids Tyr69, Leu337, Phe352, Tyr407, and Tyr444 were identified as specific residues, indicating that **4k** may bind to hydrophobic pockets within the protein and stabilize the protein–ligand complex (Figure 2). Hydrophobic interactions play a crucial role in protein–ligand binding, as they facilitate the creation of a favorable environment for the ligand within the hydrophobic pockets of the protein.

**Figure 3:**
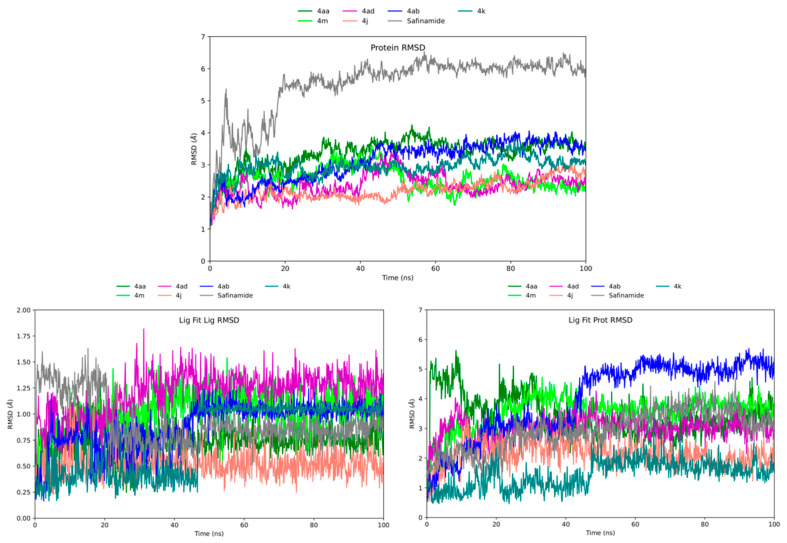
Protein RMSD graph of the Cα atoms of MAO-B throughout 100 ns MD simulations. LigFitProt RMSD graph for the 6 hit compounds and positive control throughout 100 ns MD simulations. LigFitLig RMSD graph for the 6 hit compounds and positive control throughout 100 ns MD simulations.

**Figure 4:**
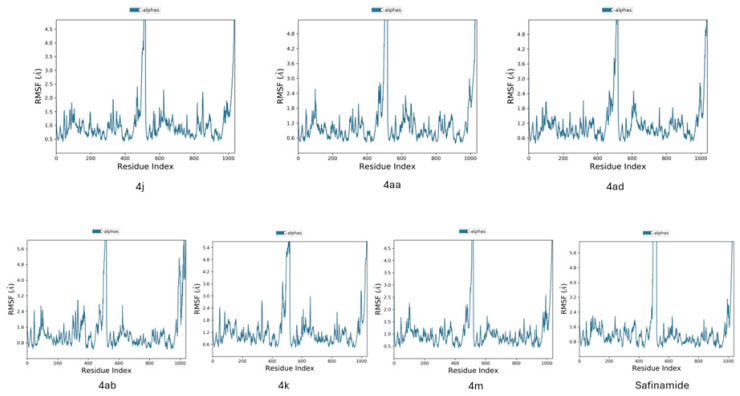
RMSF graphs for the 6 hit compounds and positive control.

In addition to hydrophobic interactions, hydrogen bonds can also play a significant role in protein–ligand binding by helping to orient the ligand within the binding pocket and contributing to the stability of the complex. During the simulation, Phe208 and Gln215 were both observed to form hydrogen bonds with **4k**. Throughout the MD simulations with **4k**, the interaction between the binding pocket residues Phe208 and Gln215 remained conserved (Figure 3 and Figure 4).

### 2.4. Binding Free Energy Analyses

**Figure 5:**
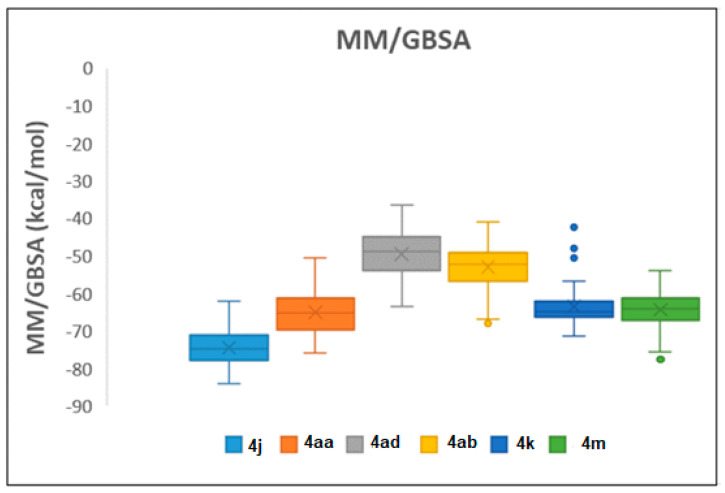
Box and whisker plot representation of MM/GBSA values of each compound with hMAO−B.

The molecular mechanics/Generalized Born surface area (MM/GBSA) is a computational method that is commonly used to predict the binding free energy of a protein–ligand complex. The method combines two different types of energy calculations: molecular mechanics (MM) and the Generalized Born surface area (GBSA) model. The MM calculates the energy of the protein–ligand complex based on the interactions between atoms, including electrostatic interactions, van der Waals forces, and bond stretching and bending. This component is useful for predicting the specific interactions between the protein and ligand, such as hydrogen bonding and hydrophobic interactions. The GBSA component calculates the solvation free energy of the complex based on the solvation of the atoms. This component takes into account the energetics of moving the solvent molecules out of the way to make room for the protein–compound complex to bind. It is useful for predicting the overall stability of the complex in solution. By combining these two components, the MM/GBSA can provide a more accurate estimate of the binding free energy of the protein–ligand complex than either method alone. The results of these calculations are summarized in Table 2, which shows the average MM/GBSA scores for each compound. The MM/GBSA scores are expressed as negative binding free energy values (ΔG_bind_), with more negative values indicating a stronger binding affinity between the ligand and the hMAO-B target. The range of binding free energy values are from −49.00 to −74.50 kcal/mol, which suggests that all compounds are potent inhibitors of the hMAO-B target. In Figure 5, the box and whisker plot of the MM/GBSA results for each compound with the hMAO-B target shows the distribution of the binding free energy values for each compound. The predicted binding free energy of the selected compounds at the binding pocket of hMAO-B provides valuable insights into their binding affinity and selectivity. By comparing the binding free energies of the selected compounds for hMAO-B with those for hMAO-A, it is clear that **4k** has a higher predicted binding affinity for hMAO-B and is more likely to be selective for hMAO-B. The average MM/GBSA score of **4k** is calculated as −63.07 ± 5.40 kcal/mol, indicating a strong predicted binding affinity for hMAO-B, which fits well with the experimental results.

### 2.5. ADME/Tox Properties

The physicochemical properties of the six selected compounds were predicted. According to pkCSM and pre ADMET, all the compounds obey Lipinski’s rule of five and Veber’s Rule, because they have less than seven rotatable bonds (Table 3). According to preADMET, the BBB value is greater than 1. As a result, all the studied propargylamines herein are classified as active in the central nervous system (CNS) (Table 3). The values for human intestinal absorption are high for all the compounds, and this signifies that these propargylamines might be absorbed better from the intestinal tract with oral administration (Table 3).

According to pkCSM, all the compounds have been predicted to be hepatotoxic. Except for the compound **4m**, all the other compounds exhibit negative AMES toxicity and, as a result, they are not mutagenic. Selegiline was incorporated as a sample of reference. As can be observed from the results, this has certain advantages (i.e., buffer solubility and pure water solubility) and disadvantages (i.e., hepatotoxicity, skin sensitivity, CYP_2C19 inhibitor)

### 2.6. Inhibitory Mechanism Discussion

**Scheme 4:**
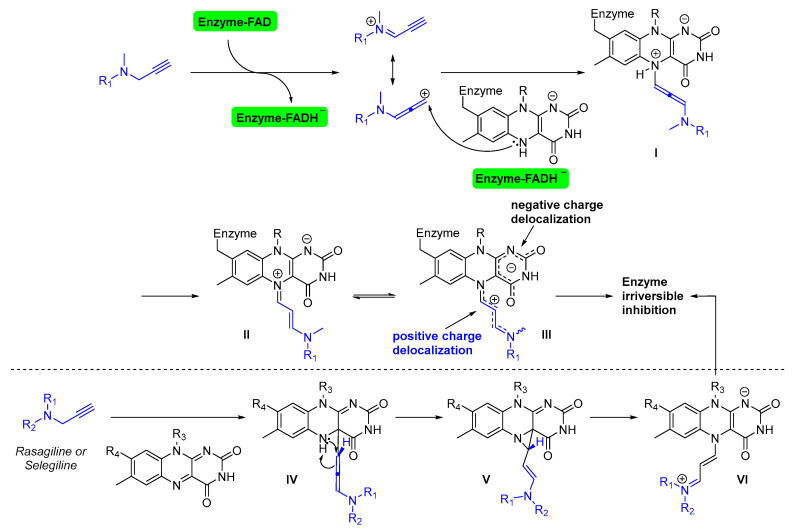
Proposed inhibition mechanism of propargylamine ASS234 (**up**) and rasagiline or selegiline (**down**).

The generally accepted MAO inhibition mechanism by propargylamine-based drugs relies on their irreversible reaction with target enzymes, suppressing their catalytic activity [37,38]. For example, in a study by Albreht, Ramsay, and co-workers published in 2018, the propargylamine-based ligand ASS234 was proposed to be initially oxidized by MAO, leading to the corresponding iminium cation and enzyme FADH^−^ (Scheme 4) [39,40]. Michael addition of the flavin nitrogen to the generated electrophilic species leads to MAO enzyme states **II** and **III**, deactivating its catalytic potential [1]. Along these lines, the key element in the propargylamines’ success as MAO inhibitors, and their distinction from naturally occurring amines within organisms, whose oxidation is naturally regulated by MAO enzymes, relies on the ability of the known propargylamine scaffolds to irreversibly bind to the enzyme. This is rationalized by the stabilization of enzyme–propargylamine structures **II** and **III**, due to their zwitterionic, charge-delocalized nature [37,41]. In 2019, Tandarić and Vianello proposed a slightly different mechanism for the inhibition of MAO enzymes by rasagiline and selegiline, based on detailed theoretical calculations [42,43]. According to this proposal, the FAD cofactor originally abstracts a hydride from the methylenic group of the propargylaminic scaffold of the inhibitors, towards the formation of **IV**, featuring an allene moiety (Scheme 4). The subsequent proton transfer to the allenic carbon center, towards a three-membered ring (**V**), followed by ring opening, leads to the same irreversible drug–enzyme inhibition (**VI**). Interestingly, taking into account that our compounds studied herein are not terminal alkynes, the deactivation mechanism suggested in the present case has to follow a different pathway. To the best of our knowledge, this is the first time such a propargylamine scaffold (i.e., bearing an internal rather than a terminal alkyne) is reported to show this type of biological activity, and we are currently mechanistically studying this effect.

## 3. Materials and Methods

All chemicals, catalysts, as well as starting materials used were received from Sigma-Aldrich (St. Louis, MO, USA), Merck (Rahway, NJ, USA), Fluorochem (Derbyshire, UK), Fluka (Buchs, Switzerland), Alfa Aesar (Haverhill, MA, USA), Fisher Scientific (Waltham, MA, USA), Acros Organics (Geel, Belgium), Lach-ner (Neratovice, Czech Republic), ChemLab (Zedelgem, Belgium), Penta (Epsom, UK), or Panreac (Barcelona, Spain). The majority of these were used without further purification; however, cyclohexanone, piperidine, morpholine, pyrrolidine, and p-methoxy benzylamine were purified via distillation prior to their use. All reactions, unless specified otherwise, were carried out under an argon atmosphere in flame-dried, Teflon-sealed screw-cap pressure tubes, Schlenk tubes, or microwave pressure tubes. Toluene was dried under phosphorous pentoxide and was stored over activated molecular sieves. The course of the reactions was monitored via GC-MS or thin layer chromatography (TLC), using silica gel 60-coated aluminum sheets (0.2 mm), absorbing at 254 nm (silica gel 60 F254), as well as using a potassium permanganate solution for visualization. All products, unless otherwise mentioned, were isolated by high-pressure gradient column chromatography, using silica gel 60 (230–400 mesh) and mixtures of hexanes/ethyl acetate or dichloromethane/methanol as the eluent system. NMR spectra were recorded on Bruker Avance-400 MHz or Varian Mercury 200 MHz instruments, using CDCl_3_ or MeOD-d_4_ as a solvent and its residual solvent peak as a reference. NMR spectroscopic data are given in the following order: chemical shift, multiplicity (s, singlet, bs, broad singlet, d, doublet, t, triplet, q, quartet, dd, doublet of doublets, dt, doublet of triplets, td, triplet of doublets, m, multiplet), coupling constant in hertz (Hz), and the number of protons. High-resolution mass spectrometry (HRMS) spectra were recorded using a QTOF maxis Impact (Bruker) spectrometer with electron spray ionization (ESI). GC-MS spectra were recorded with a Shimadzu GCMS-QP2010 Plus Chromatograph Mass Spectrometer using a MEGA (MEGA-5, FT: 0.25 μm, ID: 0.25 mm, L: 30 m, *T*_max_: 350 °C, Column ID no. 11475) column, using chloroform as the solvent. Reactions performed under microwave conditions were carried out in a dedicated CEM-Discover monomode microwave apparatus, operating at a frequency of 2.455 GHz, with continuous irradiation power from 0 to 300 W. The reactions were set up in 10 mL glass tubes, sealed with a Teflon septum and placed in the microwave cavity.

### 3.1. Synthesis of Propargylamines ***4a***–***4ae***

It must be noted that all propargylamines studied herein have been previously reported by our group and their spectroscopic data (^1^H and ^13^C NMR, as well as ESI-HRMS) are provided in the literature [27,28]. In order to study the hMAO-A and hMAO-B activities of the compounds, they are re-synthesized in the current work according to the following procedure.

#### General Procedure for the Synthesis of Propargylamines **4a**–**4m** under Mn Catalysis [28]

To a Teflon-sealed screw-cap pressure tube, flame dried and purged with Ar and equipped with a stirring bar and a rubber septum, MnBr_2_ (10.7 mg, 0.05 mmol, 0.05 equiv.) and the amine (1 mmol, 1 equiv.) substrate were added, and the mixture was stirred at rt (room temperature) until partial dilution of the catalyst. Under a flow of Ar, the alkyne (1 mmol, 1 equiv.) and the ketone (1 mmol, 1 equiv.) substrates were added, and the mixture was stirred at rt until the mixture was homogenized. The rubber septum was replaced with a Teflon screw-cap, and the reaction was sealed and heated at 130 °C for 16 h. The reaction was then cooled to rt, diluted with ethyl acetate, passed through a short silica gel plug and the filtrate was condensed in vacuo and purified via gradient column chromatography, using a mixture of hexanes/ethyl acetate. In several cases, the final products were further purified via precipitation using methanol as solvent, allowing for the precipitation of the final products at a high purity as solids.

1-(1-(phenylethynyl)cyclohexyl)piperidine (**4a**): [28,32] Product was synthesized according to the general procedure under Mn catalysis, purified with a gradient system of petroleum ether/ethyl acetate, with reagents used at 2 mmol scale, and isolated as a yellow oil at 97% yield (519 mg, 1.94 mmol). ^1^H NMR (200 MHz, CDCl_3_): *δ* 7.47–7.36 (m, 2H), 7.28–7.23 (m, 3H), 2.66 (bs, 4H), 2.11–2.05 (m, 2H), 1.83–1.34 (m, 14H).

1-(1-(phenylethynyl)cyclopentyl)pyrrolidine (**4b**): [28,32] Product was synthesized according to the general procedure under Mn catalysis, purified with a gradient system of petroleum ether/ethyl acetate, with reagents used at 2 mmol scale, and isolated as an orange/yellow oil at 75% yield (359 mg, 1.5 mmol). ^1^H NMR (200 MHz, CDCl_3_): *δ* 7.48–7.35 (m, 2H), 7.33–7.17 (m, 3H), 2.75 (t, *J* = 6.7 Hz, 4H), 2.21–1.98 (m, 2H), 1.93–1.64 (m, 10H).

1-(1-(phenylethynyl)cycloheptyl)pyrrolidine (**4c**): [28,32] Product was synthesized according to the general procedure under Mn catalysis, purified with a gradient system of petroleum ether/ethyl acetate, with reagents used at 2 mmol scale, and isolated as a yellow oil at 73% yield (390 mg, 1.46 mmol). ^1^H NMR (200 MHz, CDCl_3_): *δ* 7.42 (dd, *J_1_* = 6.5 Hz, *J_2_* = 3.0 Hz, 2H), 7.28 (dd, *J_1_* = 6.5 Hz, *J_2_* = 2.5 Hz, 3H), 2.79 (t, *J* = 6.0 Hz, 4H), 2.10–1.83 (m, 4H), 1.82–1.70 (m, 4H), 1.71–1.46 (m, 8H).

4-(1-(Phenylethynyl)cyclohexyl)morpholine (**4d**): [25,28] Product was synthesized according to the general procedure under Mn catalysis, with reagents used at 2 mmol scale under air conditions, purified with a gradient system of petroleum ether/ethyl acetate, and isolated as a yellow oil at 89% yield (480 mg, 1.78 mmol). ^1^H NMR (200 MHz, CDCl_3_): *δ* 7.52–7.37 (m, 2H), 7.31–7.23 (m, 3H), 3.81–3.70 (m, 4H), 2.79–2.65 (m, 4H), 2.07–1.95 (d, *J* = 12.0 Hz, 2H), 1.73–1.40 (m, 8H).

1-(phenylethynyl)-N, N-dipropylcyclohexan-1-amine (**4e**): [28] Product was synthesized according to the general procedure under Mn catalysis, purified with a gradient system of petroleum ether/ethyl acetate, and isolated as a pale-yellow oil at 61% yield (346 mg, 1.22 mmol). ^1^H NMR (200 MHz, CDCl_3_): *δ* 7.54–7.37 (m, 2H), 7.34–7.20 (m, 3H), 2.75–2.51 (t, *J* = 8.0 Hz, 4H), 2.23–1.99 (d, *J* = 11.0 Hz, 2H), 1.78–1.37 (m, 12H), 0.87 (t, *J* = 7.5 Hz, 6H).

1-(1-((4-chlorophenyl)ethynyl)cyclohexyl)piperidine (**4f**): [28] Product was synthesized according to the general procedure under Mn catalysis, purified with a gradient system of petroleum ether/ethyl acetate, and isolated as pale yellow crystals at 64% yield (346 mg, 1.26 mmol). ^1^H NMR (200 MHz, CDCl_3_): *δ* 7.33 (d, *J* = 8.5 Hz, 2H), 7.22 (d, *J* = 8.5 Hz, 2H), 2.63 (t, *J_1_* = 5.0 Hz, *J_2_* = 3.5 Hz, 4H), 2.03 (d, *J* = 10.0 Hz, 2H), 1.53 (m, 14H).

1-(1-(p–tolylethynyl)cyclohexyl)pyrrolidine (**4g**): [28] Product was synthesized according to the general procedure under Mn catalysis, purified with a gradient system of petroleum ether/ethyl acetate, and isolated as pale yellow crystals at 87% yield (465 mg, 1.74 mmol). ^1^H NMR (200 MHz, CDCl_3_): *δ* 7.32 (d, *J* = 8.0 Hz, 2H), 7.10 (d, *J* = 8.0 Hz, 2H), 2.89 (d, *J* = 6.0 Hz, 4H), 2.34 (s, 3H), 2.03 (t, *J* = 5.0 Hz, 2H), 1.90–1.79 (m, 4H), 1.73–1.66 (m, 8H).

1-(1-((4-chlorophenyl)ethynyl)cyclohexyl)pyrrolidine (**4h**): [28] Product was synthesized according to the general procedure under Mn catalysis, purified with a gradient system of petroleum ether/ethyl acetate, and isolated as pale yellow crystals at 77% yield (440 mg, 1.53 mmol). ^1^H NMR (200 MHz, CDCl_3_): *δ* 7.40–7.25 (d, *J* = 8.5 Hz, 2H), 7.18 (d, *J* = 8.5 Hz, 2H), 2.76–2.67 (m, 4H), 2.00–1.88 (d, *J* = 8.0 Hz, 2H), 1.81–1.38 (m, 12H).

1-(1-((4-methoxyphenyl)ethynyl)cyclohexyl)piperidine (**4i**): [28,44] Product was synthesized according to the general procedure under Mn catalysis, purified with a gradient system of petroleum ether/ethyl acetate, and isolated as pale yellow crystals at 87% yield (517 mg, 1.74 mmol). ^1^H NMR (200 MHz, CDCl_3_): *δ* 7.36 (d, *J* = 9.0 Hz, 2H), 6.81 (d, *J* = 9.0 Hz, 2H), 3.79 (s, 3H), 2.68–2.62 (t, *J* = 5.5 Hz, 4H), 2.31–1.91 (d, *J* = 12.5 Hz, 2H), 1.80–1.37 (m, 14H).

3-(1-(1-(Phenylethynyl)cyclopentyl)pyrrolidin-2-yl)pyridine (**4j**): [28,44] Product was synthesized according to the general procedure under Mn catalysis. After cooling the reaction to room temperature, crystals were formed. Ethyl acetate was added (2 × 5 mL), and the mixture was stirred until all viscous materials were removed from the reaction vessel. Then, the mixture was passed through a short silica gel plug. The remaining yellow solution was allowed to cool for 18 h, at which point, pale yellow crystals had precipitated. The solid was filtered through a sintered glass crucible, washed once with cold ethyl acetate and dried under reduced pressure, and the final product was obtained as yellow crystals at 88% yield (557 mg, 1.76 mmol). ^1^H NMR (200 MHz, CDCl_3_): *δ* 8.62 (s, 1H), 8.49–8.38 (d, *J* = 8.5 Hz, 1H), 7.82–7.69 (m, 1H), 7.51–7.37 (m, 2H), 7.34–7.13 (m, 4H), 4.30–4.22 (dd, *J_1_* = 17.5 Hz, *J_2_ =* 7.5Hz, 1H), 3.28–3.16 (s, 1H), 3.12–2.92 (q, *J* = 17.0 Hz, 1H), 2.42–2.09 (m, 2H), 2.05–1.42 (m, 10H).

2-methyl-4-(1-(2-(pyridin-3-yl)pyrrolidin-1-yl)cyclohexyl)but-3-yn-2-ol (**4k**): [28] Product was synthesized according to the general procedure under Mn catalysis, purified with a gradient system of petroleum ether/ethyl acetate, and isolated as pale yellow crystals at 81% yield (506 mg, 1.66 mmol) and >99:1 d.r., based on ^1^H NMR. ^1^H NMR (200 MHz, CDCl_3_): *δ* 8.55 (s, 1H), 8.37 (d, *J* = 5.0 Hz, 1H), 7.71 (d, *J* = 8.0 Hz, 1H), 7.16 (t, *J* = 6.5 Hz, 1H), 4.21 (d, *J* = 9.5 Hz, 1H), 3.41 (s, 1H), 3.12 (t, *J* = 6.0 Hz, 1H), 2.81 (q, *J* = 8.5 Hz, 1H), 2.15–1.93 (m, 1H), 1.93–1.74 (m, 2H), 1.80–1.60 (m, 3H), 1.55 (s, 6H), 1.49–1.32 (m, 5H), 1.34–1.15 (m, 2H), 1.10–0.83 (m, 3H).

N-cyclohexyl-1-(phenylethynyl)cyclohexan-1-amine (**4l**): [28,45] Product was synthesized according to the general procedure under Mn catalysis, purified with a gradient system of petroleum ether/ethyl acetate, and isolated as a yellowish oil at 58% yield (326 mg, 1.16 mmol). ^1^H NMR (200 MHz, CDCl_3_): *δ* 7.46–7.35 (m, 2H), 7.33–7.23 (m, 3H), 2.98–2.71 (dt, *J_1_* = 10.0 Hz, *J_2_* = 6.5 Hz, 1H), 2.04–1.84 (d, *J* = 10.0 Hz, 4H), 1.79–1.49 (m, 6H), 1.50–1.01 (m, 10H).

N-(4-methoxybenzyl)-1-(phenylethynyl)cyclohexanamine (**4m**): [24,28] Product was synthesized according to the general procedure under Mn catalysis, purified with a gradient system of petroleum ether/ethyl acetate, and isolated as an orange oil at 43% yield (275 mg, 0.86 mmol). ^1^H NMR (200 MHz, CDCl_3_): *δ* 7.53–7.40 (m, 2H), 7.36–7.27 (m, 5H), 6.86 (d, *J* = 8.5 Hz, 2H), 3.91 (s, 2H), 3.79 (s, 3H), 2.00–1.94 (m, 2H), 1.75–1.39 (m, 8H). ^13^C NMR (50 MHz, CDCl_3_) *δ*: 158.9, 133.3, 132.0, 130.0, 128.6, 128.1, 124.0, 114.1, 93.9, 85.1, 55.6, 55.5, 47.7, 38.5, 26.2, 23.3.

### 3.2. General Procedure for the Synthesis of Propargylamines ***4n***–***4ae*** under Zn Catalysis [27]

To a pressure tube, equipped with a stirring bar and a rubber septum, flame-dried and purged with Ar, Zn(OAc)_2_ (73.4 mg, 0.4 mmol, 0.2 equiv) and the amine (2 mmol, 1 equiv.) substrate were added and the mixture was allowed to stir at rt until partial dilution of the solid. Under a flow of Ar, the alkyne (2.0 mmol, 1.0 equiv.) substrate was added, and the mixture was stirred until partial dilution of the solid, followed by subsequent addition of the ketone (2.2 mmol, 1.1 equiv.) substrate and stirring under room temperature until the mixture was partially homogenized. The rubber septum was then replaced with the pressure tube cap, and the reaction was sealed and heated at 120 °C for 20 h. Afterwards, the reaction was cooled to rt, ethyl acetate (2 × 5 mL) was added, and the mixture was stirred for 5 min each time, in order to dissolve the crude reaction mixture. The mixture was then passed through a short silica gel-loaded plug, the filtrate was condensed in vacuo and the desired propargylamines were purified via gradient column chromatography, using a mixture of hexanes/ethyl acetate or dichloromethane/methanol. All propargylamines studied herein have been previously reported and their spectroscopic data (^1^H, ^13^C, and ^19^F NMR, as well as ESI-HRMS) are provided in the literature [27].

1-(1-(phenylethynyl)cyclopentyl)piperidine (**4n**): [27,46] Product was synthesized according to the general procedure under Zncatalysis, purified with a system of petroleum ether/ethyl acetate, and isolated as a yellow oil in 63% yield (319 mg, 1.26 mmol). ^1^H NMR (200 MHz, CDCl_3_): *δ* 7.41 (dd, *J_1_* = 6.5 Hz, *J_2_* = 3.0 Hz, 2H), 7.31–7.25 (m, 3H), 2.65–2.62 (m, 4H), 2.13–2.10 (m, 2H), 1.87–1.44 (m, 12H).

1-(3-Methyl-1-phenylpent-1-yn-3-yl)pyrrolidine (**4o**): [27,47] Product was synthesized according to the general procedure under Zn catalysis, purified with a system of petroleum ether/ethyl acetate, and isolated as an orange/brown oil at 82% yield (373 mg, 1.64 mmol). ^1^H NMR (200 MHz, CDCl_3_): *δ* 7.45–7.38 (m, 2H), 7.32–7.26 (m, 3H), 2.80 (t, *J* = 6.0 Hz, 4H), 1.84–1.79 (m, 4H), 1.74–1.65 (m, 2H), 1.42 (s, 3H), 1.05 (t, *J* = 7.5 Hz, 3H).

1-(3-Methyl-1-phenylhex-1-yn-3-yl)pyrrolidine (**4p**): [27,48] Product was synthesized according to the general procedure under Zn catalysis, purified with a system of petroleum ether/ethyl acetate, and isolated as an orange/brown oil at 69% yield (333 mg, 1.38 mmol). ^1^H NMR (200 MHz, CDCl_3_): *δ* 7.45–7.36 (m, 2H), 7.28–7.25 (m, 3H), 2.79 (t, *J* = 5.5 Hz, 4H), 1.85–1.74 (m, 4H), 1.73–1.47 (m, 4H), 1.43 (s, 3H), 0.95 (t, *J* = 7.0 Hz, 3H).

1-(3-Ethyl-1-phenylpent-1-yn-3-yl)pyrrolidine (**4q**): [27] Product was synthesized according to the general procedure under Zn catalysis, purified with a system of petroleum ether/ethyl acetate, and isolated as a yellow oil at 30% yield (145 mg, 0.60 mmol). ^1^H NMR (200 MHz, CDCl_3_): *δ* 7.41 (dd, *J_1_* = 6.5 Hz, *J_2_* = 3.0 Hz, 2H), 7.32–7.23 (m, 3H), 2.78 (s, 4H), 1.88–1.66 (m, 8H), 0.96 (t, *J* = 7.5 Hz, 6H).

1-(4-(Phenylethynyl)decan-4-yl)pyrrolidine (**4r**): [27] Product was synthesized according to the general procedure under Zn catalysis, purified with a system of petroleum ether/ethyl acetate, and isolated as a yellow oil at 64% yield (399 mg, 1.28 mmol). ^1^H NMR (200 MHz, CDCl_3_): *δ* 7.41 (dd, *J_1_* = 6.5 Hz, *J_2_* = 3.0 Hz, 2H), 7.32–7.24 (m, 3H), 2.75 (bs, 4H), 1.88–1.58 (m, 8H), 1.51–1.18 (m, 10H), 1.00–0.77 (m, 6H).

1-(2-Methyl-1-(phenylethynyl)cyclohexyl)pyrrolidine (**4s**): [27] Product was synthesized according to the general procedure under Zn catalysis, purified with a system of petroleum ether/ethyl acetate, and isolated as a yellow oil at 72% yield (385 mg, 1.44 mmol) and 56:44 d.r. according to ^1^H NMR. ^1^H NMR (200 MHz, CDCl_3_): *δ* 7.47–7.38 (m, 4H), 7.33–7.22 (m, 6H), 2.74 (m, 8H), 2.19–1.29 (m, 26H), 1.15 (d, *J* = 7.0 Hz, 3H, CH_3_, major diastereoisomer), 1.03 (d, *J* = 7.0 Hz, 3H, CH_3_, minor diastereoisomer).

1-(3-Methyl-1-(phenylethynyl)cyclohexyl)pyrrolidine (**4t**): [27] Product was synthesized according to the general procedure under Zn catalysis, purified with a system of petroleum ether/ethyl acetate, and isolated as a yellow oil at 82% yield (439 mg, 1.64 mmol) and 87:13 d.r. according to ^1^H NMR. ^1^H NMR (200 MHz, CDCl_3_): *δ* 7.40 (dd, *J_1_* = 6.5 Hz, *J_2_* = 3.0 Hz, 2H), 7.32–7.22 (m, 3H), 2.70 (bs, 4H), 2.12–1.97 (m, 2H), 1.87–1.07 (m, 9H), 1.02 (d, *J* = 6.0 Hz, CH_3_, minor diastereoisomer), 0.87 (d, *J* = 6.5 Hz, CH_3_, major diastereoisomer).

1-(2-(Phenylethynyl)bicyclo[2.2.1]heptan-2-yl)piperidine (**4u**): [27] Product was synthesized according to the general procedure under Zn catalysis, purified with a system of petroleum ether/ethyl acetate, and isolated as a yellow oil at 78% yield (436 mg, 1.56 mmol) and >99:1 d.r. ^1^H NMR (500 MHz, CDCl_3_): *δ* 7.42 (dd, *J_1_* = 8.0, *J_2_* = 1.5 Hz, 2H), 7.31–7.25 (m, 2H), 2.50 (bs, 4H), 2.43 (d, *J* = 3.5 Hz, 1H), 2.22 (s, 1H), 1.96 (d, *J* = 9.5 Hz, 1H), 1.94–1.86 (m, 2H), 1.58 (m, 4H), 1.51–1.40 (m, 3H), 1.38–1.35 (m, 1H), 1.34–1.32 (m, 1H), 1.32–1.21 (m, 2H).

1-(1-(p-Tolylethynyl)cyclohexyl)piperidine (**4v**): [27,46] Product was synthesized according to the general procedure under Zn catalysis, purified with a system of petroleum ether/ethyl acetate, and isolated as a yellow oil at 72% yield (405 mg, 1.44 mmol). ^1^H NMR (200 MHz, CDCl_3_): *δ* 7.33 (d, *J* = 6.5 Hz, 2H), 7.09 (d, *J* = 6.5 Hz, 2H), 2.69 (s, 4H), 2.33 (s, 3H), 2.14–2.07 (m, 2H), 1.73–1.44 (m, 14H).

1-(1-(m-Tolylethynyl)cyclohexyl)piperidine (**4w**): [27] Product was synthesized according to the general procedure under Zn catalysis, purified with a system of petroleum ether/ethyl acetate, and isolated as a yellow oil at 68% yield (383 mg, 1.36 mmol). ^1^H NMR (200 MHz, CDCl_3_): *δ* 7.30–7.05 (m, 5H), 2.69 (s, 4H), 2.33 (s, 3H), 2.10 (d, *J* = 12.5 Hz, 2H), 1.84–1.34 (m, 14H).

1-(1-((4-Methoxy-2-methylphenyl)ethynyl)cyclohexyl)piperidine (**4x**): [27] Product was synthesized according to the general procedure under Zn catalysis, purified with a system of petroleum ether/ethyl acetate, and isolated as a yellow solid at 70% yield (383 mg, 1.36 mmol). ^1^H NMR (200 MHz, CDCl_3_): *δ* 7.57 (d, *J* = 8.0 Hz, 1H), 7.48 (s, 1H), 7.24 (d, *J* = 7.5 Hz, 1H), 7.17–7.08 (m, 1H), 2.75 (s, 4H), 2.17 (d, *J* = 11.0 Hz, 2H), 1.79–1.42 (m, 14H).

1-(1-((2-Bromophenyl)ethynyl)cyclohexyl)piperidine (**4y**): [27] Product was synthesized according to the general procedure under Zn catalysis, purified with a system of petroleum ether/ethyl acetate, and isolated as an orange solid at 70% yield (485 mg, 1.40 mmol). ^1^H NMR (200 MHz, CDCl_3_): *δ* 7.57 (dd, *J_1_* = 8.0 Hz, *J_2_* = 1.5 Hz, 1H), 7.48 (dd, *J_1_* = 7.5 Hz, *J_2_* = 1.5 Hz, 1H), 7.24 (td, *J_1_* = 7.5 Hz, *J_2_* = 1.5 Hz, 1H), 7.17–7.08 (m, 1H), 2.75 (bs, 4H), 2.17 (d, *J* = 11.0 Hz, 2H), 1.79–1.42 (m, 14H).

Ethyl 1-(1-(phenylethynyl)cyclohexyl)piperidine-4-carboxylate (**4z**): [27] Product was synthesized according to the general procedure under Zn catalysis, purified with a system of petroleum ether/ethyl acetate, and isolated as a yellow oil at 67% yield (455 mg, 1.34 mmol). ^1^H NMR (200 MHz, CDCl_3_): *δ* 7.42 (dd, *J_1_* = 6.5 Hz, *J_2_* = 3.0 Hz, 2H), 7.35–7.23 (m, 3H), 4.13 (q, *J* = 7.0 Hz, 2H), 3.15 (d, *J* = 11.5 Hz, 2H), 2.42–2.18 (m, 3H), 2.13–1.43 (m, 14H), 1.24 (t, *J* = 7.0 Hz, 3H).

3-(1-(1-(Phenylethynyl)cyclohexyl)pyrrolidin-2-yl)pyridine (**4aa**): [27] Product was synthesized according to the general procedure under Zn catalysis. When the reaction was cooled, the reaction mixture turned to solid. The solid was treated with ethyl acetate (2 × 5 mL), and the mixture was stirred intensely until all viscous materials were removed from the reaction vessel. The mixture was then passed through a short silica gel-loaded plug, leaving a yellow solution, which was placed in the refrigerator overnight. Afterwards, the colorless crystals which had precipitated by then were filtered, washed with ethyl acetate, and dried under reduced pressure, forming the desired product at 70% yield (462 mg, 1.40 mmol). ^1^H NMR (200 MHz, CDCl_3_): *δ* 8.65 (s, 1H), 8.43 (d, *J* = 4.5 Hz, 1H), 7.76 (d, *J* = 8.0 Hz, 1H), 7.47 (dd, *J_1_* = 6.5 Hz, *J_2_* = 3.0 Hz, 2H), 7.37–7.27 (m, 3H), 7.21 (dd, *J_1_* = 8.0, *J_2_* = 5.0 Hz, 1H), 4.38 (d, *J* = 9.0 Hz, 1H), 3.33–3.16 (m, 1H), 2.97 (dd, *J_1_* = 16.5 Hz, *J_2_* = 8.5 Hz, 1H), 2.33–2.09 (m, 1H), 2.08–1.96 (m, 1H), 1.90–1.33 (m, 10H), 1.08 (m, 2H).

2-Methyl-4-(1-(4-phenylpiperazin-1-yl)cyclohexyl)but-3-yn-2-ol (**4ab**): [27] Product was synthesized according to the general procedure under Zn catalysis, purified with a system of petroleum ether/ethyl acetate, and isolated as an orange solid at 32% yield (209 mg, 0.64 mmol). ^1^H NMR (200 MHz, CDCl_3_): *δ* 7.32–7.19 (m, 2H), 6.93 (d, *J* = 8.5 Hz, 2H), 6.89–6.80 (m, 1H), 3.31–3.15 (m, 4H), 2.88–2.73 (m, 4H), 2.04–1.38 (m, 16H).

N-Benzyl-1-(phenylethynyl)cyclohexanamine (**4ac**): [24,27] Product was synthesized according to the general procedure under Zn catalysis, purified with a system of petroleum ether/ethyl acetate, and isolated as a yellow oil at 71% yield (411 mg, 1.42 mmol). ^1^H NMR (200 MHz, CDCl_3_): *δ* 7.56–7.16 (m, 10H), 3.97 (s, 2H), 2.01–1.95 (m, 2H), 1.77–1.41 (m, 8H).

4-(1-((4-Fluorobenzyl)amino)cyclohexyl)-2-methylbut-3-yn-2-ol (**4ad**): [27] Product was synthesized according to the general procedure under Zn catalysis, purified with a system of petroleum ether/ethyl acetate, and isolated as an orange oil at 51% yield (295 mg, 1.02 mmol). ^1^H NMR (200 MHz, CDCl_3_): *δ* 7.36–7.20 (m, 2H), 7.05–6.94 (m, 2H), 3.83 (s, 2H), 2.97–2.88 (m, 1H), 1.89–1.77 (m, 3H), 1.69–1.58 (m, 4H), 1.56 (s, 6H), 1.48–1.32 (m, 4H).

N-Benzyl-2-(phenylethynyl)bicyclo[2.2.1]heptan-2-amine (**4ae**): [27] Product was synthesized according to the general procedure under Zn catalysis, purified with a system of petroleum ether/ethyl acetate, and isolated as a yellow oil at 33% yield (199 mg, 0.66 mmol) and >99:1 d.r. ^1^H NMR (200 MHz, CDCl_3_): *δ* 7.51–7.21 (m, 10H), 4.01 (d, *J* = 12.5 Hz, 1H), 3.71 (d, *J* = 12.5 Hz, 1H), 2.51 (d, *J* = 3.0 Hz, 1H), 2.28 (s, 1H), 2.23–2.11 (m, 1H), 2.02 (m, 2H), 1.61–1.21 (m, 6H).

### 3.3. In Vitro MAO Enzyme Inhibitory Activities

The Novaroli et al. [49] method was used with minor modifications to study the biological activities of MAO-A and MAO-B in vitro. A preincubation step was carried out at 37 °C for 10 min, in which 140 μL of 0.1 M potassium phosphate-buffered solution at pH 7.4, 8 μL of 0.75 mM kynuramine, and 2 μL of a dimethyl sulfoxide (DMSO) solution (i.e., final DMSO and inhibitor concentrations of 1% (*v*/*v*) and 0–1 μM, respectively). Diluted human recombinant enzyme (50 μL) was added to achieve the final protein concentration, and the mixture was incubated at 37 °C. After 20 min, the reaction was stopped with the addition of 75 μL of 2 M NaOH. MAO-A activity was measured for 20 min using 0.06 mM kynuramine as the substrate at 316 nm, while MAO-B activity was measured for 10 min using 0.6 mM benzylamine at 250 nm. The product compound generated by the enzyme 4-quinolinol (which is fluorescent) was measured at Ex 310 nm/Em 400 nm using a microplate reader (Thermo Scientific-Multiskan GO). Each measurement was repeated three times. DMSO was used as control. IC_50_ values were then calculated. Benzylamine, kynuramine, R-(−)-Deprenyl hydrochloride (Selegiline) recombinant human MAO-A and MAO-B were purchased from Sigma-Aldrich (USA). The determination of IC_50_ values for inhibition of MAO enzymes involved the use of recombinant human MAO-A and MAO-B enzymes (purchased from Sigma-Aldrich). The initial rates of oxidation were measured in a 1 mL cuvette containing 50 mM of sodium phosphate (pH 7.4) at 25 °C, as described previously, except for the substrate concentrations and assay times. To measure the initial rates of oxidation, a 1 mL cuvette containing 50 mM of sodium phosphate (pH 7.4) at 25 °C was employed, following established protocols, with exceptions regarding substrate concentrations and assay durations. In this study, the activity of MAO-A was assessed using 0.06 mM of kynuramine as the substrate, monitoring absorbance changes at 316 nm over a 20 min period. Meanwhile, the assessment of MAO-B activity employed 0.6 mM of benzylamine as the substrate, with absorbance changes measured at 250 nm for 10 min. Substrate addition initiated the reaction, and the reaction rates were quantified as alterations in absorbance per minute.

### 3.4. Molecular Modeling

The compounds that were selected based on the in vitro results underwent molecular modeling simulations in order to thoroughly study their structural and dynamic characteristics. The simulations employed a range of techniques, including molecular docking, MD simulations, and binding free energy calculations. By utilizing these techniques, we could gain insight into the atomic-level interactions between the selected compounds and the target proteins. Additionally, the simulations enabled us to predict the binding affinity and binding mode of the compounds.

### 3.5. Target Protein Preparation

The Protein Data Bank (PDB) was used to obtain the X-ray diffraction (XRD) structures of the hMAO-A and hMAO-B targets with the PDB IDs 2Z5X [50] and 1S3B [36], respectively. In crystallographic studies, it is frequently observed that certain regions of the protein remain unresolved in the X-ray diffraction data, leading to the absence or incomplete representation of residues in the final structure. In the specific case of the hMAO-B crystal structure, it was found that the C-terminal residues, which are typically situated within a lipid bilayer, were not present in the structure. To complete the structure, the missing residues were modeled in our previous study and used as target structures in the current simulations [51].

### 3.6. Ligand Preparation

Proper ligand preparation is critical to obtain accurate and reliable results from these simulations. Selected compounds were optimized to ensure that they were in a reasonable conformation for docking or other simulations using the OPLS3e force field [51]. These compounds were subjected to energy minimization, which involves minimizing the potential energy of the molecule by adjusting its bond lengths and angles, in order to obtain a more stable structure using the LigPrep module of the Maestro Molecular Modeling Suit [52]. Compounds may exist in different ionization states and tautomeric forms, which can affect their binding affinity and specificity. Therefore, the selected compounds were typically ionized and tautomeric forms were generated to account for these possible variations using the Epik tool [53].

### 3.7. Receptor Grid Generation

The receptor grid is a three-dimensional grid that represents the specific binding site on the protein where the ligand is anticipated to interact. The selection of the binding site on the protein was determined by the known or predicted location of the active site. In the case of hMAO-A, the Cartesian coordinates (40.75, 26.78, −14.78) of the co-crystallized ligand were employed as the center of the grid box. Similarly, for hMAO-B, the coordinates (14.72, 128.54, 24.67) of the co-crystallized ligand were used as the center of the grid box.

### 3.8. Molecular Docking Studies

The prepared compounds underwent docking into the receptor grid using the IFD approach [54]. During the IFD process, both the receptor binding pocket residues and ligand undergo conformational changes to optimize their interactions. The docking algorithm assesses the potential energy of the compound at each point on the grid and searches for the most favorable orientation and conformation that result in the lowest energy. The predicted binding modes are subsequently ranked using a scoring function. This scoring function takes into account various factors including the energy of the complex, as well as considerations such as hydrogen bonding, electrostatic interactions, and van der Waals forces. By evaluating the binding affinity of each predicted compound–receptor complex, the scoring function aids in determining the strength of the binding between the compound and the receptor. No constraint was applied during the docking simulations (Glide/SP). Residues were refined with 5 Å ligand conformations. Glide re-docking was performed with by Glide/XP for top 20 poses.

### 3.9. Molecular Dynamics Simulations

By conducting MD simulations on the compound–protein complex, valuable information can be obtained regarding the stability of the binding mode as well as the dynamic behavior exhibited by the complex. These MD simulations offer insights into the thermodynamics and kinetics of the compound–protein interaction, enabling a deeper understanding of the underlying processes. Additionally, the simulations can provide detailed information about the specific interactions occurring between the ligand and the residues of the protein. To initiate MD simulations on the compound–protein complexes, the initial structure of the complex, obtained through IFD, was utilized as the starting ligand–target complex pose. The system underwent an energy minimization process to eliminate any unfavorable contacts and reduce its potential energy. Subsequently, the system was equilibrated under specific conditions, such as constant temperature and pressure, to attain a stable state. Throughout the simulations, the temperature of all systems was regulated at 310 K using the Nosé–Hoover constant temperature method [46] and constant pressure (1 atm) was used. Once equilibrated, the production MD simulations were performed under NPT ensemble over a period of 100 ns, during which the position and velocity of each atom in the system is calculated at each time step using Desmond [47]. The integration time step was 2 fs to observe the time interval between successive updates of the atomic positions and velocities in the simulation. Throughout the MD simulations, a comprehensive analysis of the interactions between the compound and protein residues was conducted. The stability of the binding mode was evaluated by tracking the RMSD of the compound’s position relative to its initial position. Additionally, the dynamic behavior of the complexes was assessed by observing the RMSF of both the protein residues and the compound.

### 3.10. Binding Free Energies Calculated by Molecular Mechanics/Generalized Born Surface Area (MM/GBSA)

The MM/GBSA method offers a rapid and effective approach for estimating the binding free energy between multiple ligands and a protein target. This method combines molecular mechanics calculations, which account for the interactions between the compound and protein, with a Generalized Born solvent model that describes the solvation free energy of the complex. The following equation is used to calculate binding free energy (ΔG(bind)):

ΔG(bind) = ΔG(solv) + ΔE(MM) + ΔG(SA)

where ∆G(solv) is the difference between the complex’s GBSA solvation energy and the sum of the unliganded protein and inhibitor solvation energies; ∆E(MM) is the difference between the sum of the unliganded protein and inhibitor energy and the minimized energies of the complex; and ∆G(SA) is the difference between the complex’s surface area energies and the sum of the unliganded protein and inhibitor’s surface area energies. The energy of optimum free receptors, free ligand, and a complex of the ligand with a receptor is calculated by Prime MM-GBSA. Snapshots of the compound–protein complexes were extracted from the MD trajectory at regular intervals. For each snapshot, the molecular mechanics energy of the compound–protein complex was computed, considering factors such as van der Waals interactions, electrostatic interactions, and solvation energies. The solvation energy was determined utilizing the Generalized Born model, which estimates the solvation energy based on the molecular surface area and electrostatic potential of the complex. The binding free energy of each studied compound was then estimated employing Schrodinger’s Prime module [55]. The VSGB solvation model was used during the simulations. Flexible residue distances were set as 3 Å from the ligand during the sampling [56,57].

### 3.11. ADMET Calculations

The studied molecules were sketched in ChemDraw and converted into .smi format. The pkCSM [58] and preADMET [31] servers were used to predict the drug-likeness of these compounds and some other toxicological properties.

## 4. Conclusions

Neurodegenerative diseases are still not fully treatable due to their complexity. Therefore, new, more selective, and more efficient molecules aiming at these diseases are needed. In this study, several quaternary propargylamine compounds previously reported by our group were re-synthesized through the KA^2^ multicomponent reaction. This method entails the coupling of an amine, a ketone, and a terminal alkyne in one step, utilizing an environmentally friendly metal catalyst. The quaternary propargylamine molecular scaffold was then evaluated against the enzymes hMAO-A and hMAO-B and was found to exert considerable inhibitory activity. The IC_50_ values for hMAO-A range between 765.6 nM and 861.6 nM and the IC_50_ values for hMAO-B range between 152.1 nM and 164.7 nM. The six most potent of these propargylamines were also thoroughly studied theoretically. They were found to obey the Lipinski rule, not showing any predicted toxicity. The key interactions of these molecules were studied using molecular docking and MD simulations, as well as MM/GBSA calculations. All six propargylamines exert favorable binding on both the hMAO-A and hMAO-B enzymes. Interestingly, such a propargylamine scaffold, bearing an internal alkyne rather than a terminal one, is, for the first time, reported to show activity against monoaminoxidases, to the best of our knowledge. Along these lines, the molecules reported herein are very promising leads for treatments of neurodegenerative disorders. Using the above results, we initiated a research program using rational drug design and a combination of molecular docking and MD simulations in silico, to discover even more potent candidates. New molecules have thus been designed, synthesized, and are currently being evaluated. The most potent will be subjected to in vivo experiments, in order to further explore their beneficial effect.

## Figures and Tables

**Table 1:** IC_50_ values and selectivity indexes of the propargylamine derivatives evaluated on MAO-A and MAO-B enzymes.

Compound Code	IC_50_ hMAO-B (nM) ^a^	IC_50_ hMAO-A (nM) ^a^	SI ^b^
**4a**	274.2 ± 2.68	-	
**4f**	175.3 ± 2.11	-	
**4k**	152.1 ± 1.95	797.1 ± 6.73	5.24
**4j**	164.7 ± 2.03	861.5 ± 6.97	5.23
**4h**	171.8 ± 2.10	-	
**4g**	189.2 ± 2.24	-	
**4e**	286.1 ± 2.74	-	
**4l**	294.5 ± 3.06	-	
**4b**	182.4 ± 2.21	-	
**4i**	172.2 ± 2.10	-	
**4m**	165.3 ± 2.01	802.9 ± 7.03	4.86
**4c**	183.1 ± 2.12	-	
**4d**	174.3 ± 2.08	-	
**4q**	317.2 ± 3.24	-	
**4ad**	161.3 ± 1.97	765.6 ± 6.42	4.74
**4r**	323.4 ± 3.21	-	
**4u**	345.9 ±3.51	-	
**4t**	191.5 ± 2.41	-	
**4y**	277.4 ± 2.71	-	
**4p**	203.1 ± 2.24	-	
**4aa**	168.3 ± 2.12	791.3 ± 6.72	4.70
**4ac**	278.4 ± 2.71	-	
**4s**	281.3 ± 2.75	-	
**4u**	276.7 ± 2.65	-	
**4ab**	167.2 ± 2.01	845.8 ± 6.83	5.06
**4x**	267.4 ± 2.52	-	
**4z**	213.1 ± 2.24	-	
**4w**	281.9 ± 2.72	-	
**4ae**	321.6 ± 2.94	-	
**4n**	272.3 ± 2.54	-	
**4o**	312.1 ± 3.17	-	
**Selegiline (irreversible inhibitor)**	5.82 ± 0.04	1452 ± 71	249.5
**Safinamide (reversible inhibitor) ^c^**	98	580,000	5918.4

^a^ IC_50_ values represent the concentration of inhibitor required to decrease enzyme activity by 50% and are the means of 3 independent experiments, each performed in triplicate. Data were expressed as mean ± SD, ^b^ SI = IC_50_ (hMAO-A)/IC_50_ (hMAO-B) ratio (SI: selectivity index). ^c^ From Leonetti et al. (Available online: https://pubs.acs.org/doi/abs/10.1021/jm070725e accessed on: 2 April 2024).

**Table 2:** Two-dimensional structures, IFD scores, average MM/GBSA scores, and IC_50_ values of selected six hit compounds with hMAO-A and hMAO-B.

**Comp.**	**2D Structure**	**IFD (hMAO-A) (kcal/mol)**	**IFD (hMAO-B) (kcal/mol)**	**Average MM/GBSA (hMAO-A) (kcal/mol)**
**4j**	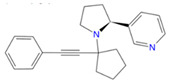	−10.18	−11.74	−60.11 ± 4.61
**4aa**	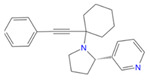	−12.19	−11.44	−57.19 ± 4.66
**4ad**	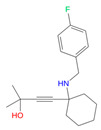	−12.09	−10.33	−52.24 ± 5.27
**4ab**	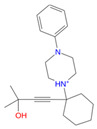	−13.22	−11.80	−50.81 ± 6.66
**4k**	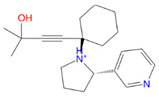	−12.42	−10.83	−47.30 ± 4.57
**4m**	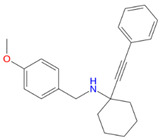	−12.64	−11.97	−43.63 ± 4.11
**positive control**	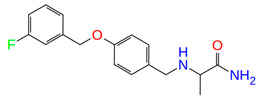	−12.28	−12.05	−54.95 ± 4.84
**Comp.**	**2D Structure**	**Average MM/GBSA (hMAO-B) (kcal/mol)**	**IC_50_ hMAO-A (nM)**	**IC_50_ hMAO-B** **(nM)**
**4j**	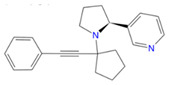	−74.5 ± 64.56	861.5 ± 6.97	164.7 ± 2.03
**4aa**	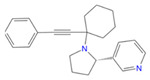	−64.50 ± 5.75	791.3 ± 6.72	168.3 ± 2.12
**4ad**	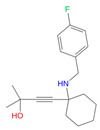	−49.00 ± 5.92	765.6 ± 6.42	161.3 ± 1.97
**4ab**	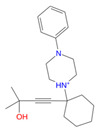	−52.53 ± 5.79	845.8 ± 6.83	167.2 ± 2.01
**4k**	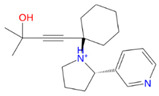	−63.07 ± 5.40	797.1 ± 6.73	152.1 ± 1.95
**4m**	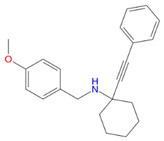	−63.91 ± 4.83	−802.9 ± 7.03	−165.3 ± 2.01
**Positive control**	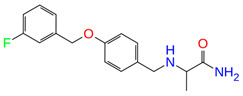	−74.23 ± 5.09	-	-

Positive control: Safinamide.

**Table 3:** The physicochemical parameters for these six compounds according to pkCSM and SwissADME.

**Properties**	**4k**	**4j**	**4m**
Molecular weight	312.45	316.44	345.48
LogP	3.69	4.58	5.283
Rotatable bonds	2	2	2
Hydrogen bond acceptors	3	2	2
Hydrogen bond donors	1	0	1
Surface area	139.66	144.470	156.40
Water solubility	−3.17	−4.47	−4.50
**Properties**	**4k**	**4j**	**4m**
*Toxicity*			
AMES toxicity	No	No	Yes
Max. tolerated dose (human)	−0.14	−0.60	−0.02
hERG I inhibitor	No	Yes	No
hERG II inhibitor	No	Yes	Yes
Oral rat acute toxicity (LD50)	2.73	2.83	2.99
Oral rat chronic toxicity	1.42	1.07	1.37
Hepatotoxicity	Yes	Yes	Yes
Skin sensitization	No	No	No
ADME	**4k**	**4j**	**4m**
BBB	2.94	2.26	13.98
Buffer_solubility_mg_L	3.91	338.63	97.37
Caco2	28.89	25.10	54.53
CYP_2C19_inhibition	Non	Non	Non
CYP_2C9_inhibition	Non	Non	Inhibitor
CYP_2D6_inhibition	Inhibitor	Inhibitor	Inhibitor
CYP_2D6_substrate	Substrate	Substrate	Substrate
CYP_3A4_inhibition	Inhibitor	Non	Non
CYP_3A4_substrate	Weakly	Weakly	Weakly
HIA	95.99	100	99.72
MDCK	60.88	57.83	48.16
Pgp_inhibition	Inhibitor	Non	Inhibitor
Plasma_Protein_Binding	90.24	85.52	96.90
Pure_water_solubility_mg_L	2462.22	133.67	9.40
Skin_Permeability	−1.36	−1.80	−1.27
**Properties**	**4ad**	**4aa**	**4ab**
Molecular weight	289.39	330.47	326.48
LogP	3.24	4.97	3.28
Rotatable bonds	3	2	2
Hydrogen bond acceptors	2	2	3
Hydrogen bond donors	2	0	1
Surface area	126.31	150.83	146.20
Water solubility	−2.58	−4.59	−3.49
**Properties**	**4ad**	**4aa**	**4ab**
*Toxicity*			
AMES toxicity	No	No	No
Max. tolerated dose (human)	−0.14	−0.29	−0.53
hERG I inhibitor	No	No	No
hERG II inhibitor	Yes	Yes	Yes
Oral rat acute toxicity (LD50)	2.64	2.24	2.78
Oral rat chronic toxicity	1.73	0.46	0.81
Hepatotoxicity	Yes	Yes	Yes
Skin ssensitization	No	No	No
ADME	**4ad**	**4aa**	**4ab**
BBB	8.41	5.91	9.92
Buffer_solubility_mg_L	9.47	101.39	0.60
Caco2	50.67	23.32	27.75
CYP_2C19_inhibition	Non	Non	Non
CYP_2C9_inhibition	Non	Non	Non
CYP_2D6_inhibition	Inhibitor	Inhibitor	Inhibitor
CYP_2D6_substrate	Substrate	Substrate	Substrate
CYP_3A4_inhibition	Non	Non	Non
CYP_3A4_substrate	Weakly	Weakly	Weakly
HIA	93.53	100.00	98.36
MDCK	1.28	66.63	45.59
Pgp_inhibition	Inhibitor	Inhibitor	Inhibitor
Plasma_Protein_Binding	100.00	86.35	89.64
Pure_water_solubility_mg_L	141.66	49.74	180.37
Skin_Permeability	−1.15	−1.48	−0.87
**Properties**	**Selegiline**		
Molecular weight	187.286		
LogP	2.1826		
Rotatable bonds	4		
Hydrogen bond acceptors	1		
Hydrogen bond donors	0		
Surface area	86.745		
Water solubility	−2.246		
**Properties**	**Selegiline**		
*Toxicity*			
AMES toxicity	No		
Max. tolerated dose (human)	0.32		
hERG I inhibitor	No		
hERG II inhibitor	No		
Oral rat acute toxicity (LD50)	2.18		
Oral rat chronic toxicity	1.80		
Hepatotoxicity	Yes		
Skin sensitization	Yes		
ADME	**Selegiline**		
BBB	4.43		
Buffer_solubility_mg_L	1594.05		
Caco2	31.94		
CYP_2C19_inhibition	Inhibitor		
CYP_2C9_inhibition	Non		
CYP_2D6_inhibition	Inhibitor		
CYP_2D6_substrate	Substrate		
CYP_3A4_inhibition	Non		
CYP_3A4_substrate	Substrate		
HIA	100.00		
MDCK	164.08		
Pgp_inhibition	Inhibitor		
Plasma_Protein_Binding	53.20		
Pure_water_solubility_mg_L	4487.36		
Skin_Permeability	−0.79		

## Data Availability

Data are contained within the article.

## Supplementary Materials

The following supporting information can be downloaded at https://www.mdpi.com/article/10.3390/molecules29112486/s1. Figures S1: ^1^H (200 MHz) and ^13^C (50 MHz) in CDCl_3_ for **4a**; Figure S2: ^1^H (200 MHz) and ^13^C (50 MHz) in CDCl_3_ for **4b**; Figure S3: ^1^H (200 MHz) and ^13^C (50 MHz) in CDCl_3_ for **4c**; Figure S4: ^1^H (200 MHz) and ^13^C (50 MHz) in CDCl_3_ for **4d**; Figure S5: ^1^H (200 MHz) and ^13^C (50 MHz) in CDCl_3_ for **4e**; Figure S6: ^1^H (200 MHz) and ^13^C (50 MHz) in CDCl_3_ for **4f**; Figure S7: ^1^H (200 MHz) and ^13^C (50 MHz) in CDCl_3_ for **4g**; Figure S8: ^1^H (200 MHz) and ^13^C (50 MHz) in CDCl_3_ for **4h**; Figure S9: ^1^H (200 MHz) and ^13^C (50 MHz) in CDCl_3_ for **4i**; Figure S10: ^1^H (200 MHz) and ^13^C (50 MHz) in CDCl_3_ for **4j**; Figure S11: ^1^H (200 MHz) and ^13^C (50 MHz) in CDCl_3_ for **4k**; Figure S12: ^1^H (200 MHz) and ^13^C (50 MHz) in CDCl_3_ for **4l**; Figure S13: ^1^H (400 MHz) and ^13^C (50 MHz) in CDCl_3_ for **4m**; Figure S14: ^1^H (200 MHz) and ^13^C (50 MHz) in CDCl_3_ for **4n**; Figure S15: ^1^H (200 MHz) and ^13^C (50 MHz) in CDCl_3_ for **4p**; Figure S16: ^1^H (200 MHz) and ^13^C (50 MHz) in CDCl_3_ for **4q**; Figure S17: ^1^H (400 MHz) and ^13^C (50 MHz) in CDCl_3_ for **4r**; Figure S18: ^1^H (200 MHz) and ^13^C (50 MHz) in CDCl_3_ for **4s**; Figure S19: ^1^H (200 MHz) and ^13^C (50 MHz) in CDCl_3_ for **4t**; Figure S20: ^1^H (200 MHz) and ^13^C (50 MHz) in CDCl_3_ for **4u**; Figure S21: ^1^H (200 MHz) and ^13^C (50 MHz) in CDCl_3_ for **4v**; Figure S22: ^1^H (200 MHz) and ^13^C (50 MHz) in CDCl_3_ for **4w**; Figure S23: ^1^H (200 MHz) and ^13^C (50 MHz) in CDCl_3_ for **4x**; Figure S24: ^1^H (200 MHz) and ^13^C (50 MHz) in CDCl_3_ for **4y**; Figure S25: ^1^H (200 MHz) and ^13^C (50 MHz) in CDCl_3_ for **4z**; Figure S26: ^1^H (200 MHz) and ^13^C (50 MHz) in CDCl_3_ for **4aa**; Figure S27: ^1^H (200 MHz) and ^13^C (50 MHz) in CDCl_3_ for **4ab**; Figure S28: ^1^H (200 MHz) and ^13^C (50 MHz) in CDCl_3_ for **4ac**; Figure S29: ^1^H (200 MHz), ^13^C (50 MHz) and ^19^F (188 MHz) in CDCl_3_ for **4ad**; Figure S30: ^1^H (200 MHz) and ^13^C (50 MHz) in CDCl_3_ for **4ae**.

## References

[1] Lauder K., Toscani A., Scalacci N., Castagnolo D. (2017). Synthesis and Reactivity of Propargylamines in Organic Chemistry. Chem. Rev..

[2] Marco-Contelles J., Unzeta M., Bolea I., Esteban G., Ramsay R.R., Romero A., Martínez-Murillo R., Carreiras M.C., Ismaili L. (2016). ASS234 As a New Multi-Target Directed Propargylamine for Alzheimer’s Disease Therapy. Front. Neurosci..

[3] Wang J.Z., Xia Y.Y., Grundke-Iqbal I., Iqbal K. (2013). Abnormal hyperphosphorylation of tau: Sites, regulation, and molecular mechanism of neurofibrillary degeneration. J. Alzheimers Dis..

[4] Zindo F.T., Joubert J., Malan S.F. (2015). Propargylamine as functional moiety in the design of multifunctional drugs for neurodegenerative disorders: MAO inhibition and beyond. Future Med. Chem..

[5] Behl T., Kaur D., Sehgal A., Singh S., Sharma N., Zengin G., Andronie-Cioara F.L., Toma M.M., Bungau S., Bumbu A.G. (2021). Role of Monoamine Oxidase Activity in Alzheimer’s Disease: An Insight into the Therapeutic Potential of Inhibitors. Molecules.

[6] Manzoor S., Hoda N.A. (2020). A comprehensive review of monoamine oxidase inhibitors as Anti-Alzheimer’s disease agents: A review. Eur. J. Med. Chem..

[7] Carradori S., Secci D., Petzer J.P. (2018). MAO inhibitors and their wider applications: A patent review. Expert Opin. Ther. Pat..

[8] Putnins E.E., Goebeler V., Ostadkarampour M. (2021). Monoamine Oxidase-B Inhibitor Reduction in Pro-Inflammatory Cytokines Mediated by Inhibition of cAMP-PKA/EPAC Signaling. Front. Pharmacol..

[9] Mathew B., Carradori S., Guglielmi P., Uddin M.S. (2020). New Aspects of Monoamine Oxidase B Inhibitors: The Key Role of Halogens to Open the Golden Door. Curr. Med. Chem..

[10] Das T., Saha S.C., Sunita K., Majumder M., Ghorai M., Mane A.B., Prasanth D.A., Kumar P., Pandey D.K., Al-Tawaha A.R. (2022). Promising botanical-derived monoamine oxidase (MAO) inhibitors: Pharmacological aspects and structure-activity studies. South. Afr. J. Bot..

[11] Nambiar M.P., Jayadevan S., Babu B.K., Biju A.R. (2022). Computational studies on the structural variations of MAO-A and MAO-B inhibitors—An in silico docking approach. Indian. J. Biochem. Biophys..

[12] Peshkov V.A., Pereshivko O.P., Van der Eycken E.V. (2012). A walk around the A3-coupling. Chem. Soc. Rev..

[13] Zorba L.P., Egaña E., Gomez-Bengoa E., Vougioukalakis G.C. (2021). Zinc Iodide Catalyzed Synthesis of Trisubstituted Allenes from Terminal Alkynes and Ketones. ACS Omega.

[14] Jayaprakash K., Venkatachalam C.S., Balasubramanian K.K. (1999). A convenient one-pot synthesis of N-aryl-3-pyrrolines. Tetrahedron Lett..

[15] Clique B., Monteiro N., Balme G. (1999). A one pot synthesis of various pyrrolidines via a tandem Michael addition-transition metal-catalysed cyclisation reaction. Tetrahedron Lett..

[16] Polindara-García L.A., Miranda L.D. (2012). Two-Step Synthesis of 2,3-Dihydropyrroles via a Formal 5-endo Cycloisomerization of Ugi 4-CR/Propargyl Adducts. Org. Lett..

[17] Goutham K., Rao Mangina N.S.V.M., Suresh S., Raghavaiah P., Karunakar G.V. (2014). Gold-catalysed cyclisation of N-propargylic β-enaminones to form 3-methylene-1-pyrroline derivatives. Org. Biomol. Chem..

[18] Ojima I., Vu A.T., Lee S.Y., McCullagh J.V., Moralee A.C., Fujiwara M., Hoang T.H. (2002). Rhodium-Catalyzed Silylcarbocyclization (SiCaC) and Carbonylative Silylcarbocyclization (CO–SiCaC) Reactions of Enynes. J. Am. Chem. Soc..

[19] Kushwaha K., Malakar C.C., Stas S., Lemiere F., Tehrani K.A. (2015). Indium(iii)-catalyzed tandem synthesis of 2-alkynyl-3,3-dichloropyrrolidines and their conversion to 3-chloropyrroles. RSC Adv..

[20] Suzuki S., Saito A. (2017). Single-Step Synthesis of Iodinated Oxazoles from N-Propargyl Amides Mediated by I2/Iodosylbenzene/Trimethylsilyl Trifluoromethanesulfonate Systems. J. Org. Chem..

[21] Wang B., Chen Y., Zhou L., Wang J., Tung C.-H., Xu Z. (2015). Synthesis of Oxazoles by Tandem Cycloisomerization/Allylic Alkylation of Propargyl Amides with Allylic Alcohols: Zn(OTf)_2_ as π Acid and σ Acid Catalyst. J. Org. Chem..

[22] García-Domínguez P., Fehr L., Rusconi G., Nevado C. (2016). Palladium-catalyzed incorporation of atmospheric CO_2_: Efficient synthesis of functionalized oxazolidinones. Chem. Sci..

[23] Zorba L.P., Vougioukalakis G.C. (2021). The Ketone-Amine-Alkyne (KA2) coupling reaction: Transition metal-catalyzed synthesis of quaternary propargylamines. Coord. Chem. Rev..

[24] Pereshivko O.P., Peshkov V.A., Van der Eycken E.V. (2010). Unprecedented Cu(I)-Catalyzed Microwave-Assisted Three-Component Coupling of a Ketone, an Alkyne, and a Primary Amine. Org. Lett..

[25] Cheng M., Zhang Q., Hu X.-Y., Li B.-G., Ji J.-X., Chan A.S.C. (2011). Gold-Catalyzed Direct Intermolecular Coupling of Ketones, Secondary Amines, and Alkynes: A Facile and Versatile Access to Propargylic Amines Containing a Quaternary Carbon Center. Adv. Synth. Catal..

[26] Pierce C.J., Nguyen M., Larsen C.H. (2012). Copper/Titanium Catalysis Forms Fully Substituted Carbon Centers from the Direct Coupling of Acyclic Ketones, Amines, and Alkynes. Angew. Chem. Int. Ed..

[27] Tzouras N.V., Neofotistos S.P., Vougioukalakis G.C. (2019). Zn-Catalyzed Multicomponent KA2 Coupling: One-Pot Assembly of Propargylamines Bearing Tetrasubstituted Carbon Centers. ACS Omega.

[28] Neofotistos S.P., Tzouras N.V., Pauze M., Gómez-Bengoa E., Vougioukalakis G.C. (2020). Manganese-Catalyzed Multicomponent Synthesis of Tetrasubstituted Propargylamines: System Development and Theoretical Study. Adv. Synth. Catal..

[29] Adejumo T.T., Tzouras N.V., Zorba L.P., Radanovic D., Pevec A., Grubišic S., Mitic D., Andelkovic K.K., Vougioukalakis G.C., Cobeljic B. (2020). Synthesis, Characterization, Activity, C. and DFT Calculations of Zn(II) Hydrazone Complexes. Molecules.

[30] Adejumo T.T., Danopoulou M., Zorba L.P., Pevec A., Zlatar M., Radanović D., Savić M., Gruden M., Anđelković K.K., Turel I. (2023). Correlating Structure and KA2 Catalytic Activity of Zn(II) Hydrazone Complexes. Eur. J. Inorg. Chem..

[31] https://preadmet.webservice.bmdrc.org/.

[32] Finberg J.P.M., Rabey J.M. (2016). Inhibitors of MAO-A and MAO-B in Psychiatry and Neurology. Front. Pharmacol..

[33] Choi J.W., Jang B.K., Cho N., Park J.H., Yeon S.K., Ju E.J., Lee Y.S., Han G., Pae A.M., Kim D.J. (2015). Synthesis of a series of unsaturated ketone derivatives as selective and reversible monoamine oxidase inhibitors. Bioorg. Med. Chem..

[34] Pisani L., Catto M., Nicolotti O., Grossi G., Di Braccio M., Soto-Otero R., Mendez-Alvarez E., Stefanachi A., Gadaleta D., Carotti A. (2013). Fine molecular tuning at position 4 of 2H-chromen-2-one derivatives in the search of potent and selective monoamine oxidase B inhibitors. Eur. J. Med. Chem..

[35] Tsugeno Y., Ito A. (1997). A key amino acid responsible for substrate selectivity of monoamine oxidase A and B. J. Biol. Chem..

[36] Binda C., Hubalek F., Li M., Herzig Y., Sterling J., Edmondson D.E., Mattevi A. (2004). Crystal structures of monoamine oxidase B in complex with four inhibitors of the N-propargylaminoindan class. J. Med. Chem..

[37] Albreht A., Vovk I., Mavri J., Marco-Contelles J., Ramsay R.R. (2018). Evidence for a Cyanine Link Between Propargylamine Drugs and Monoamine Oxidase Clarifies the Inactivation Mechanism. Front. Chem..

[38] Szewczuk L.M., Culhane J.C., Yang M., Majumdar A., Yu H., Cole P.A. (2007). Mechanistic Analysis of a Suicide Inactivator of Histone Demethylase LSD1. Biochemistry.

[39] Chajkowski-Scarry S., Rimoldi J.M. (2014). Monoamine Oxidase A and B Substrates: Probing the Pathway for Drug Development. Fut. Med. Chem..

[40] Lang D., Kalgutkar A., Lee J., Scott Obach R., Fisher M. (2003). Non-P450 Mediated Oxidative Metabolism of Xenobiotics. Drug Metabolizing Enzymes.

[41] Kalir A., Sabbagh A., Youdim M.B.H. (1981). Selective acetylenic ‘suicide’ and reversible inhibitors of monoamine oxidase types a and B. Br. J. Pharm..

[42] Tandarić T., Vianello R. (2019). Computational Insight into the Mechanism of the Irreversible Inhibition of Monoamine Oxidase Enzymes by the Antiparkinsonian Propargylamine Inhibitors Rasagiline and Selegiline. ACS Chem. Neurosci..

[43] Hosseini-Sarvari M., Moeini F. (2014). Nano copper(i) oxide–zinc oxide catalyzed coupling of aldehydes or ketones, secondary amines, and terminal alkynes in solvent-free conditions. New J. Chem..

[44] Shah A.P., Sharma A.S., Jain S., Shimpi N.G. (2018). Microwave assisted one pot three component synthesis of propargylamine, tetra substituted propargylamine and pyrrolo[1,2-a]quinolines using CuNPs@ZnO–PTh as a heterogeneous catalyst. New J. Chem..

[45] Sugiishi T., Nakamura H. (2012). Zinc(II)-Catalyzed Redox Cross-Dehydrogenative Coupling of Propargylic Amines and Terminal Alkynes for Synthesis of N-Tethered 1,6-Enynes. J. Am. Chem. Soc..

[46] Hoover W.G. (1985). Canonical dynamics: Equilibrium phase-space distributions. Phys. Rev. A Gen. Phys..

[47] Bosica G., Abdilla R. (2017). The KA2 coupling reaction under green, solventless, heterogeneous catalysis. J. Mol. Catal. A Chem..

[48] (2011). Desmond, v4.9.

[49] Novaroli L., Reist M., Favre E., Carotti A., Catto M., Carrupt P.A. (2005). Human recombinant monoamine oxidase B as reliable and efficient enzyme source for inhibitor screening. Bioorg Med. Chem..

[50] Son S.-Y., Ma J., Kondou Y., Yoshimura M., Yamashita E., Tsukihara T. (2008). Structure of human monoamine oxidase A at 2.2-Å resolution: The control of opening the entry for substrates/inhibitors. Proc. Natl. Acad. Sci. USA.

[51] Is Y.S., Durdagi S., Aksoydan B., Yurtsever M. (2018). Proposing Novel MAO-B Hit Inhibitors Using Multidimensional Molecular Modeling Approaches and Application of Binary QSAR Models for Prediction of Their Therapeutic Activity, Pharmacokinetic and Toxicity Properties. ACS Chem. Neurosci..

[52] Release S. (2017). 2017-2: LigPrep.

[53] Shelley J.C., Choletti A., Frye L.L., Greenwood J.R., Timlin M.R., Uchimaya M. (2007). Epik: A software program for pK(a) prediction and protonation state generation for drug-like molecules. J. Comput. Aided Mol. Des..

[54] Sherman W., Day T., Jacobson M.P., Friesner R.A., Farid R. (2006). Novel Procedure for Modeling Ligand/Receptor Induced Fit Effects. J. Med. Chem..

[55] Jacobson M.P., Pincus D.L., Rapp C.S., Day T.J.F., Honig B., Shaw D.E., Friesner R.A. (2004). A Hierarchical Approach to All-Atom Protein Loop Prediction. Proteins: Struct. Funct. Bioinform..

[56] Shan Y., Kim E.T., Eastwood M.P., Dror R.O., Seeliger M.A., Shaw D.E. (2011). How does a drug molecule find its target binding site?. J. Am. Chem. Soc..

[57] Bashford D., Case D.A. (2000). Generalized born models of macromolecular solvation effects. Annu. Rev. Phys. Chem..

[58] Pires D.E.V., Blundell T.L., Ascher D.B. (2015). pkCSM: Predicting Small-Molecule Pharmacokinetic and Toxicity Properties Using Graph-Based Signatures. J. Med. Chem..

