# Supplementary material for: Are Terminal Alkynes Necessary for MAO-A/MAO-B Inhibition? A New Scaffold Is Revealed
Source: Molecules. 2024 May 24;29(11):2486. doi: 10.3390/molecules29112486 (PMC11174103; doi:10.3390/molecules29112486)
Supplement: Supplementary file 1 [file molecules-29-02486-s001.zip › molecules-2973003-supplementary.pdf]

Supporting Information

Are terminal alkynes necessary for MAO-A/MAO-B inhibition? A new scaffold is revealed

Panagiou Mavroeidi [1], Leandros P. Zorba [1], Nikolaos V. Tzouras [1], Stavros P. Neofotistos [1], Nikitas Georgiou [1], Kader Sahin [2], Murat Şentürk [3], Serdar Durdagi [4,5,6,*], Georgios C. Vougioukalakis [1,*] and Thomas Mavromoustakos [1,*]

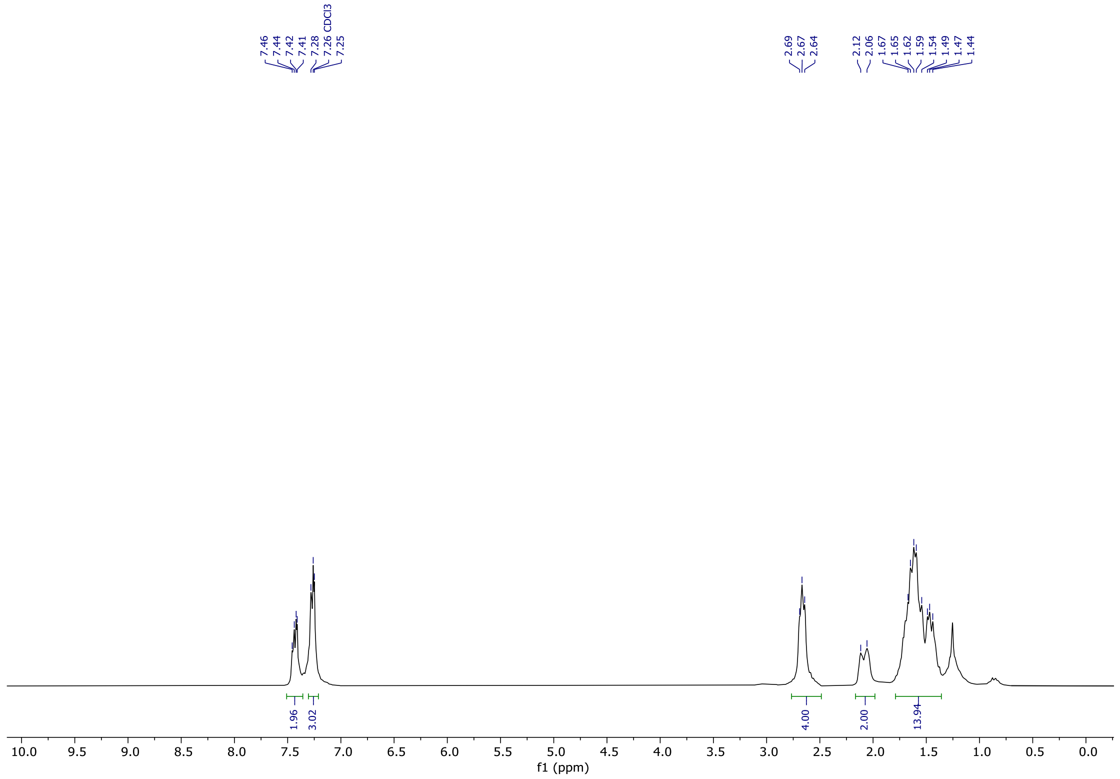

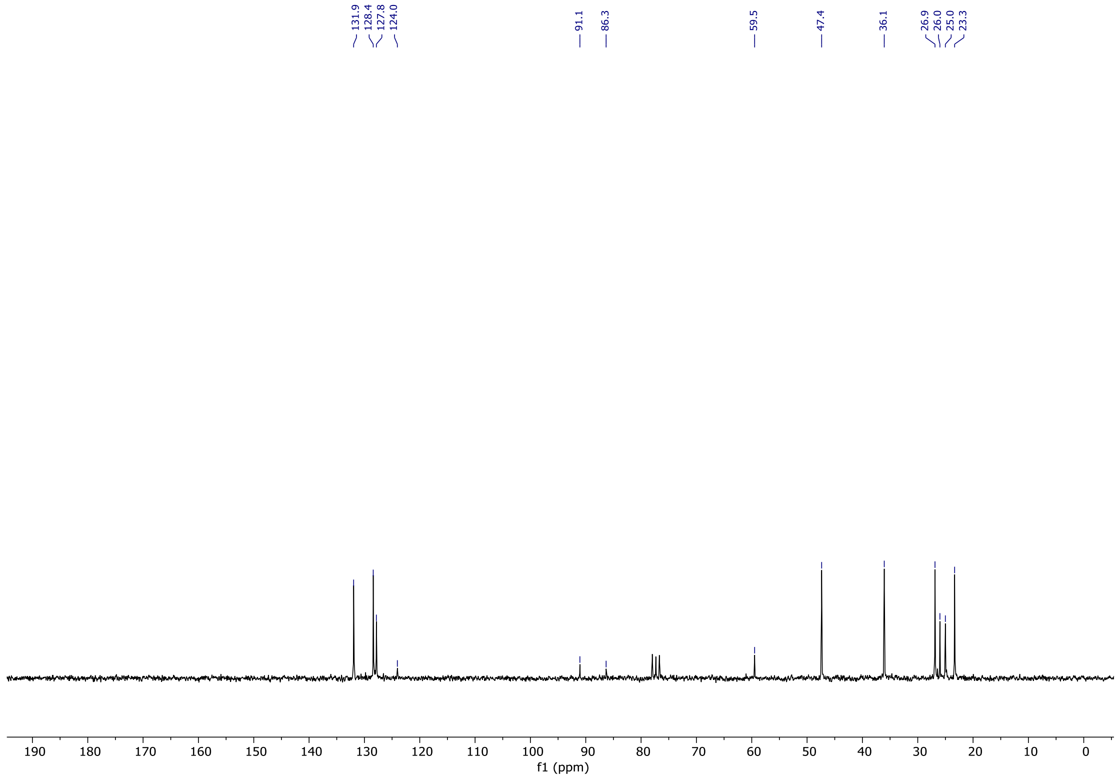

Figure S1: ^{1}H (200 MHz) and ^{13}C (50 MHz) in $CDCl_3$ for **4a**.

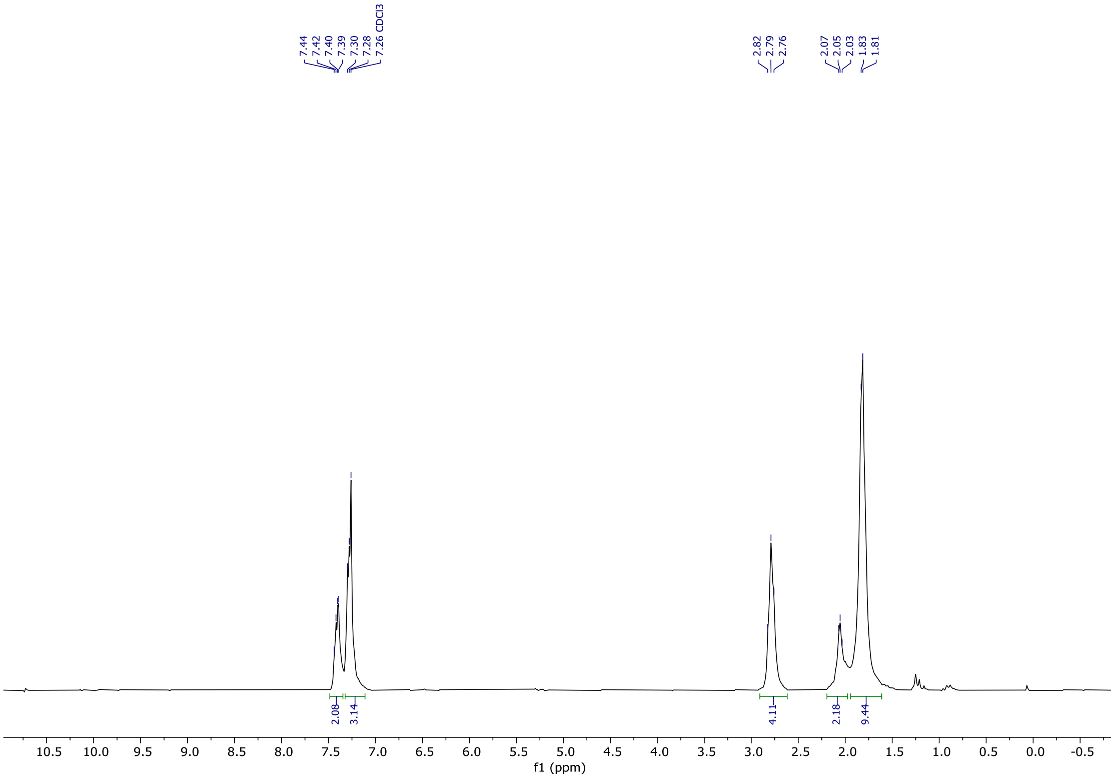

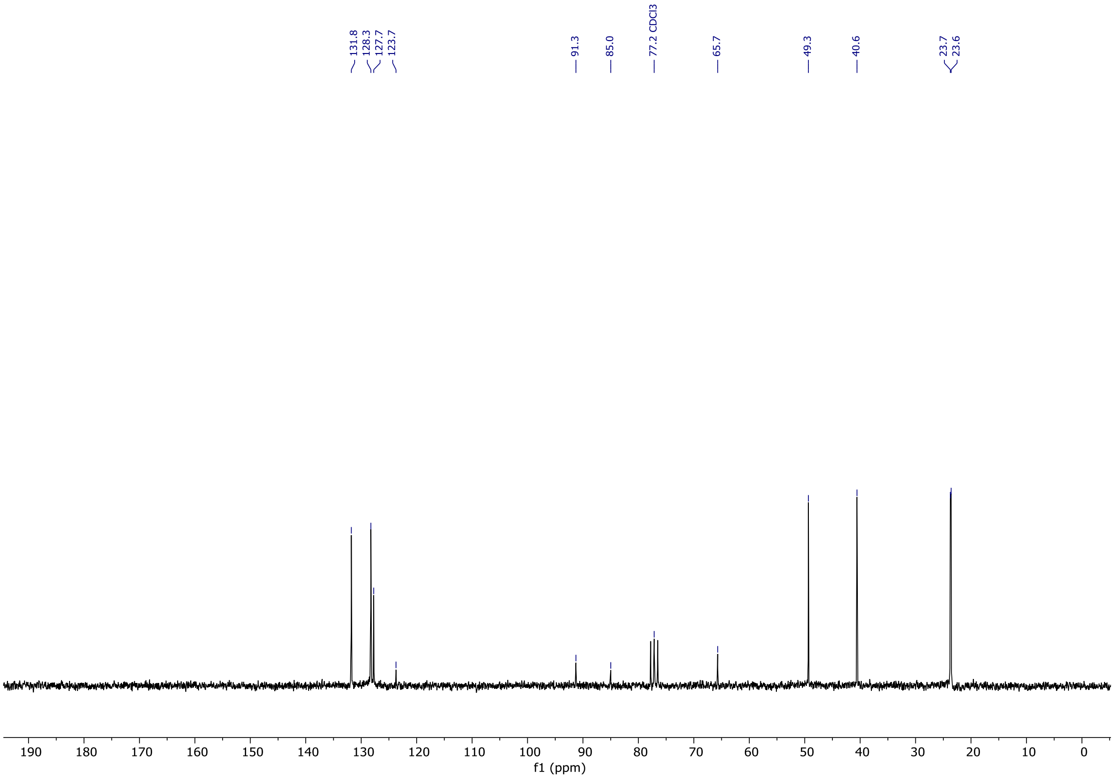

Figure S2: ^{1}H (200 MHz) and ^{13}C (50 MHz) in $CDCl_3$ for **4b**.

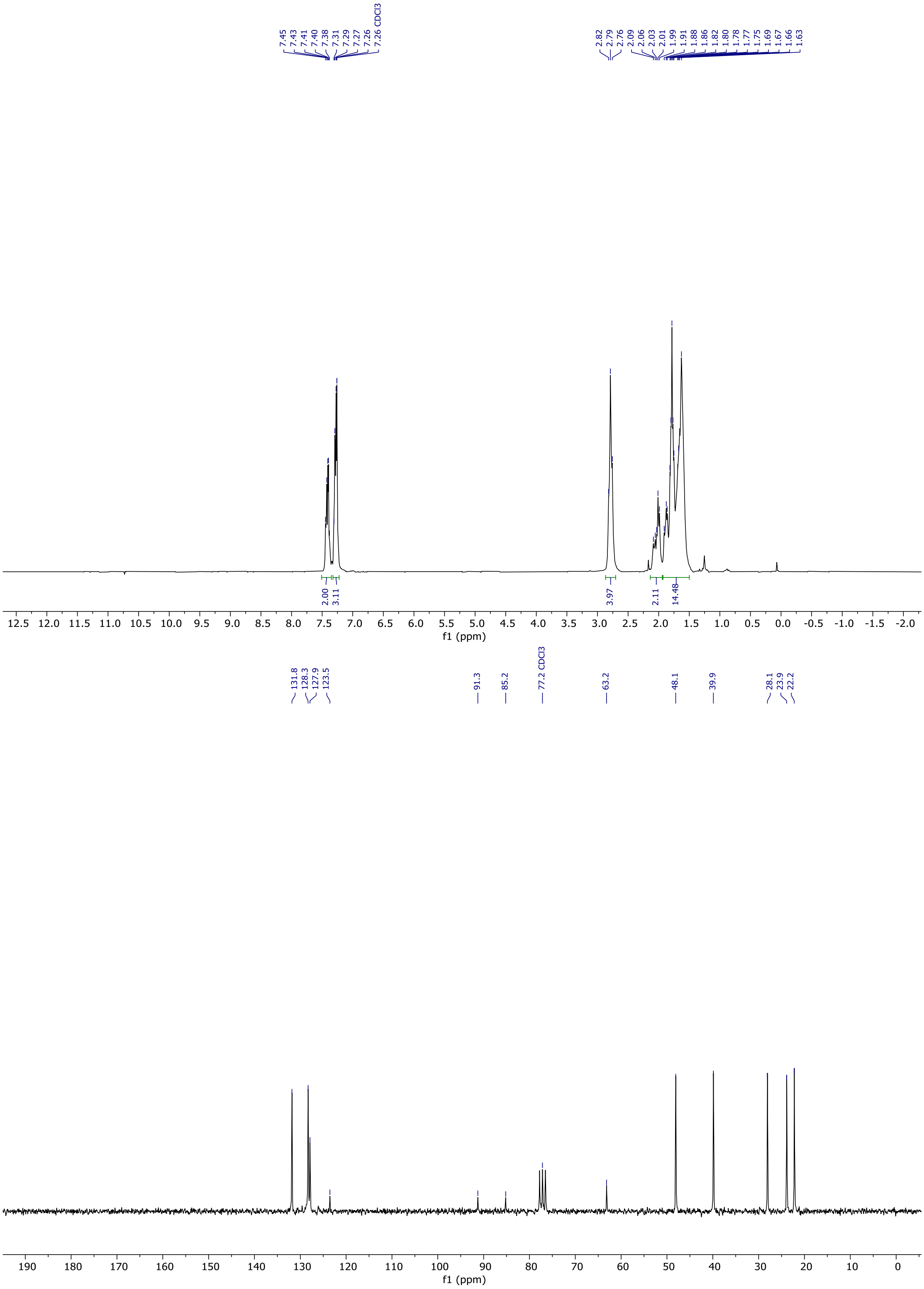

Figure S3: ^{1}H (200 MHz) and ^{13}C (50 MHz) in $CDCl_3$ for **4c**.

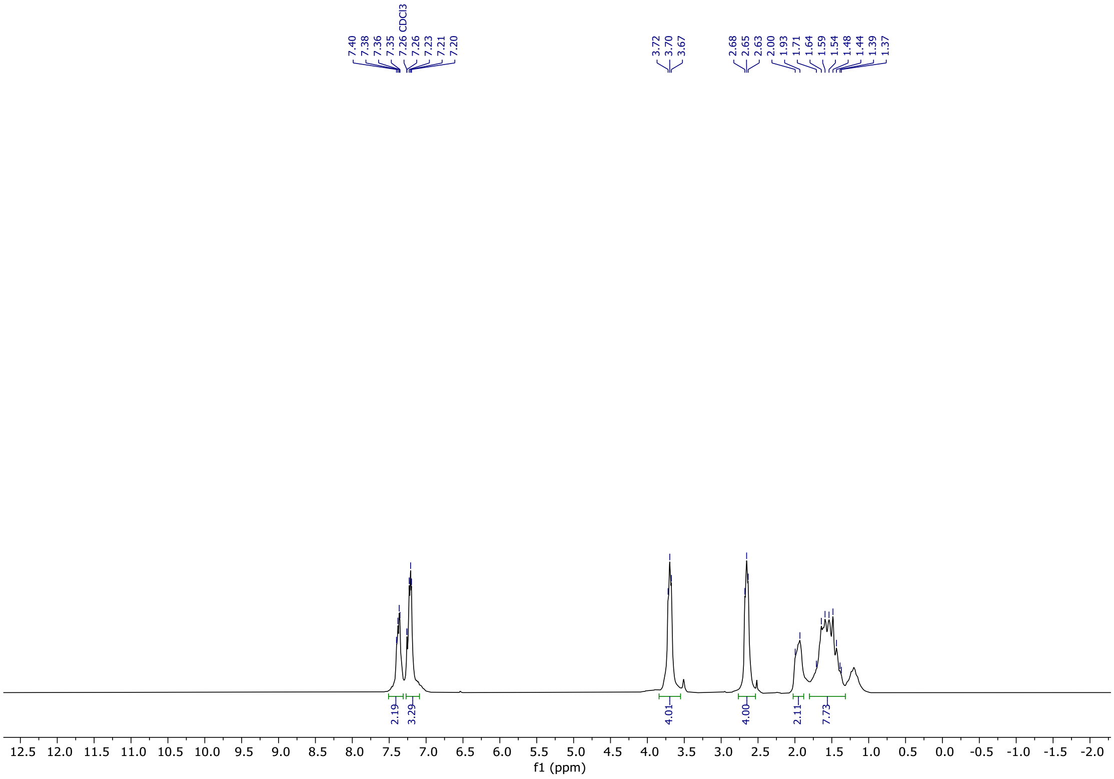

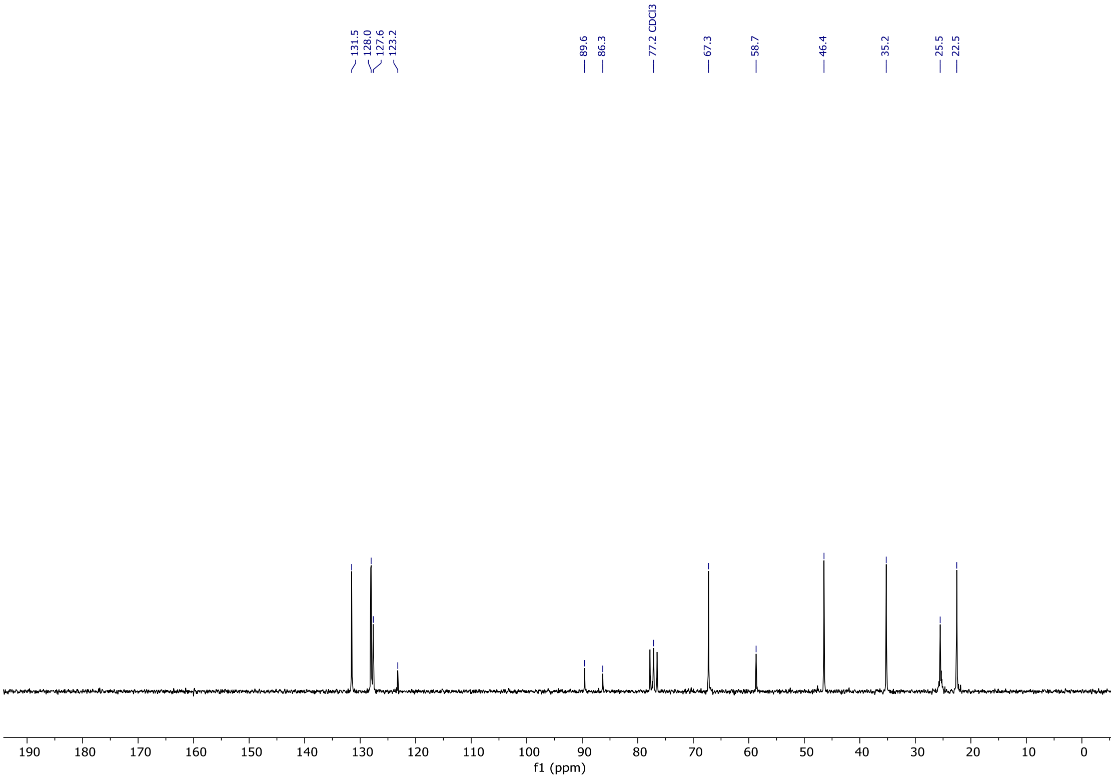

Figure S4: ^{1}H (200 MHz) and ^{13}C (50 MHz) in $CDCl_3$ for **4d**.

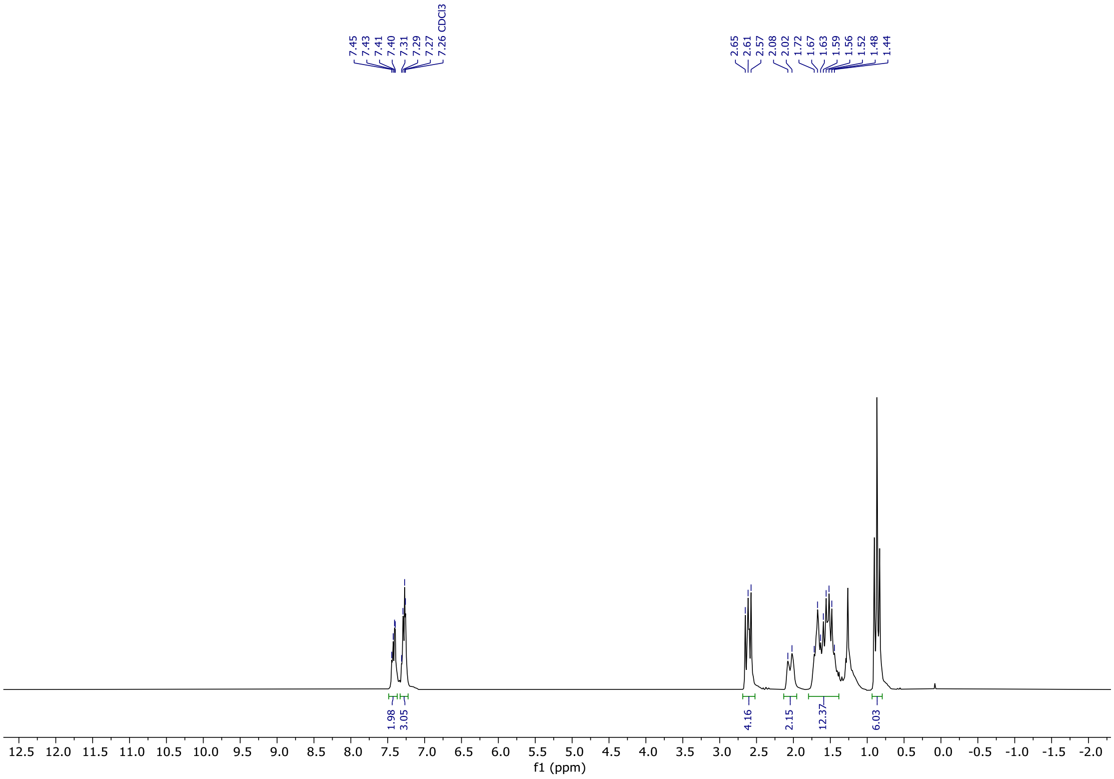

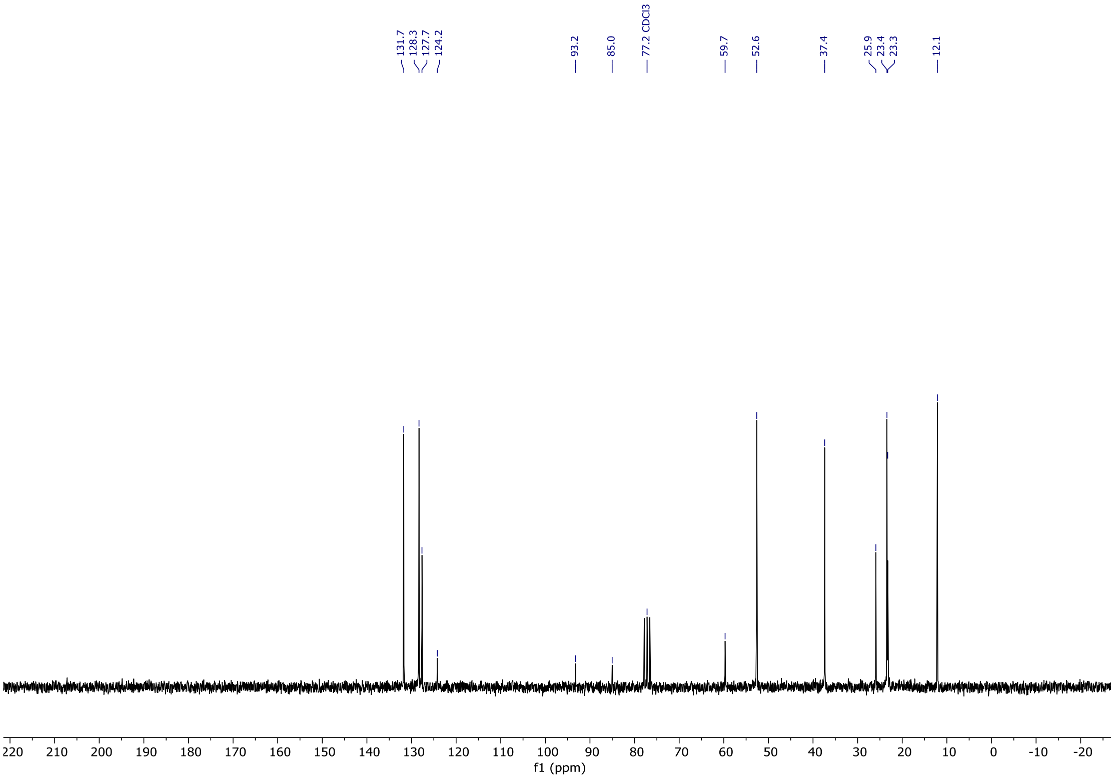

Figure S5: ^{1}H (200 MHz) and ^{13}C (50 MHz) in $CDCl_3$ for **4e**.

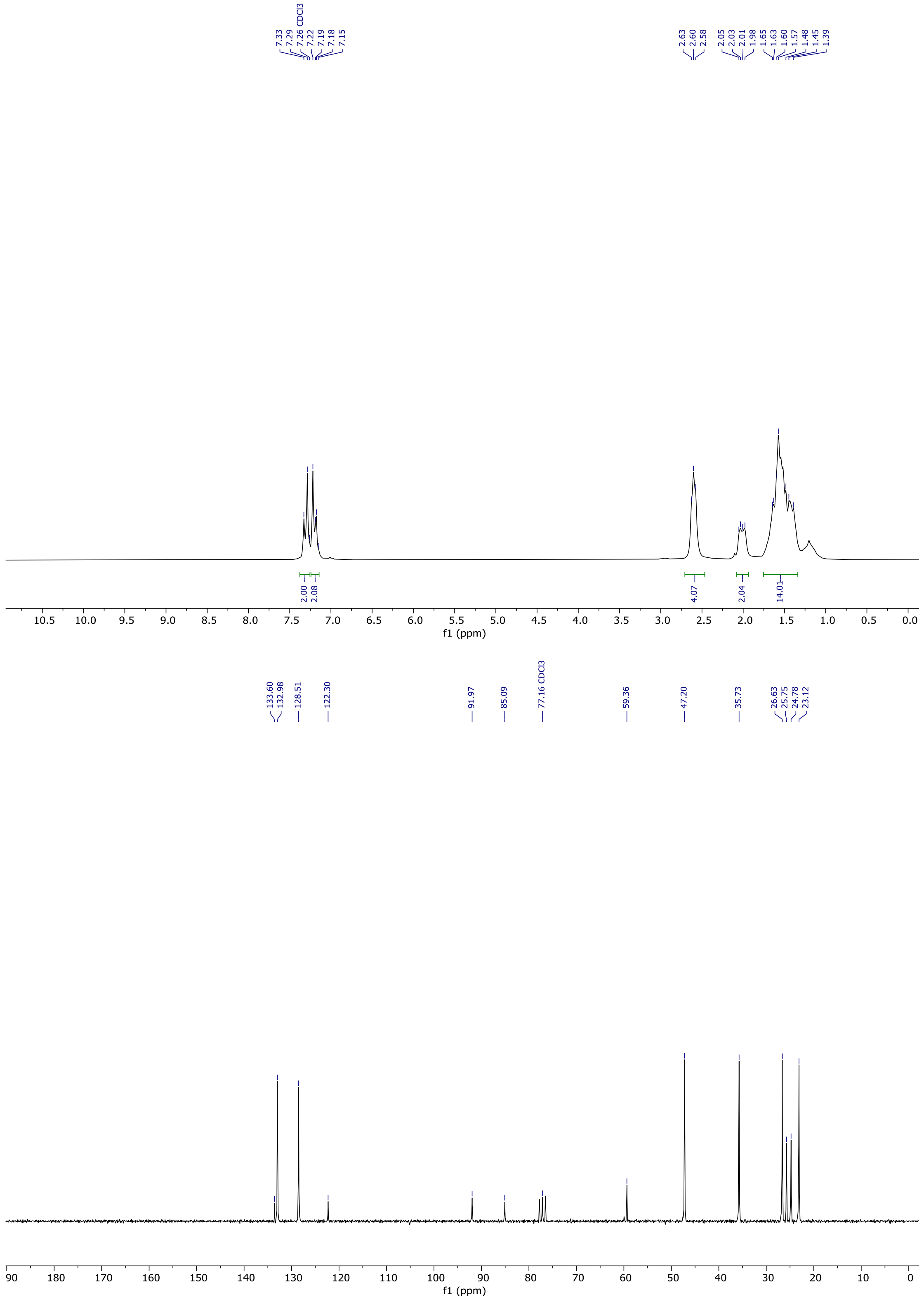

Figure S6: ^{1}H (200 MHz) and ^{13}C (50 MHz) in $CDCl_3$ for **4f**.

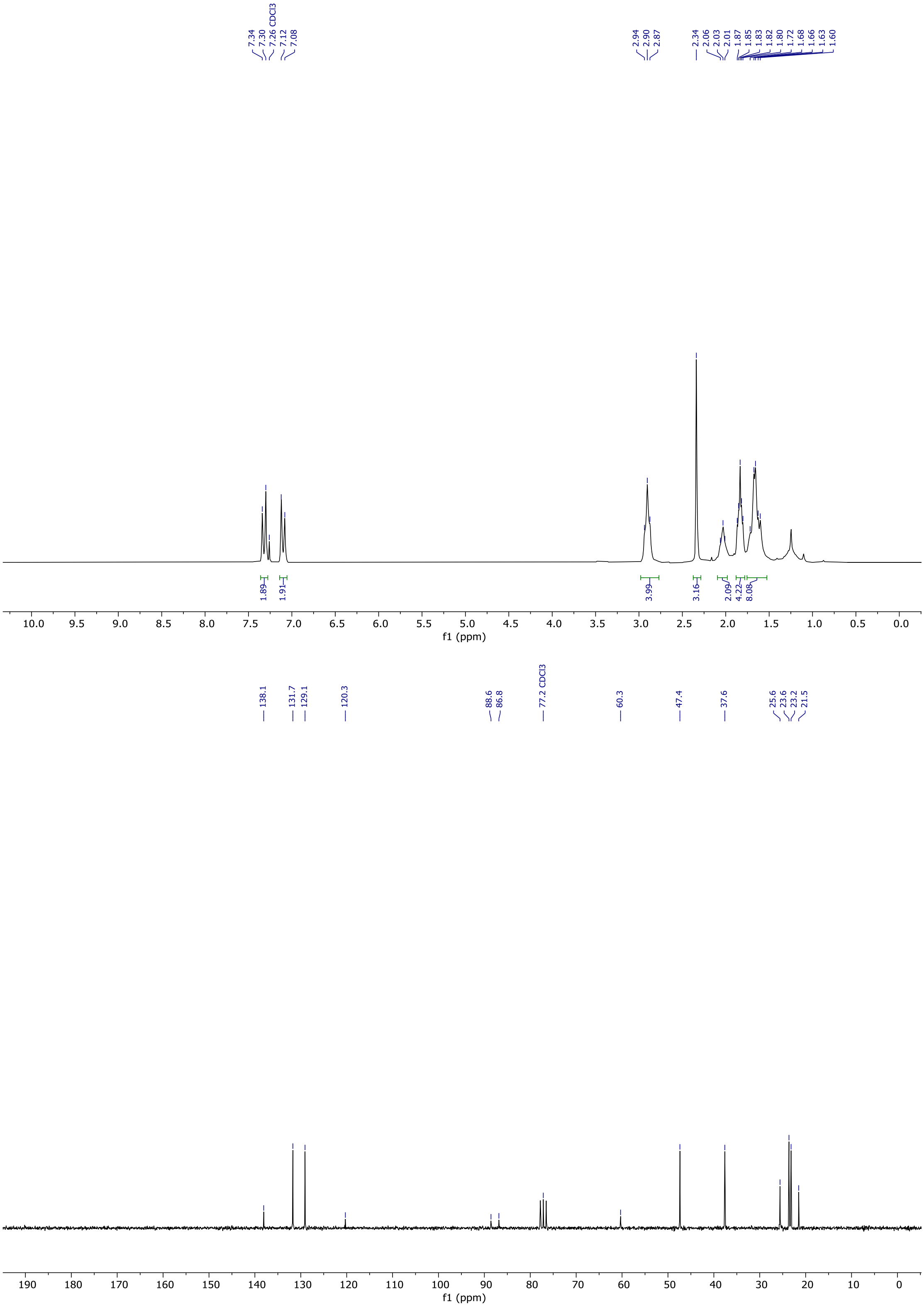

Figure S7: ^{1}H (200 MHz) and ^{13}C (50 MHz) in $CDCl_3$ for **4g**.

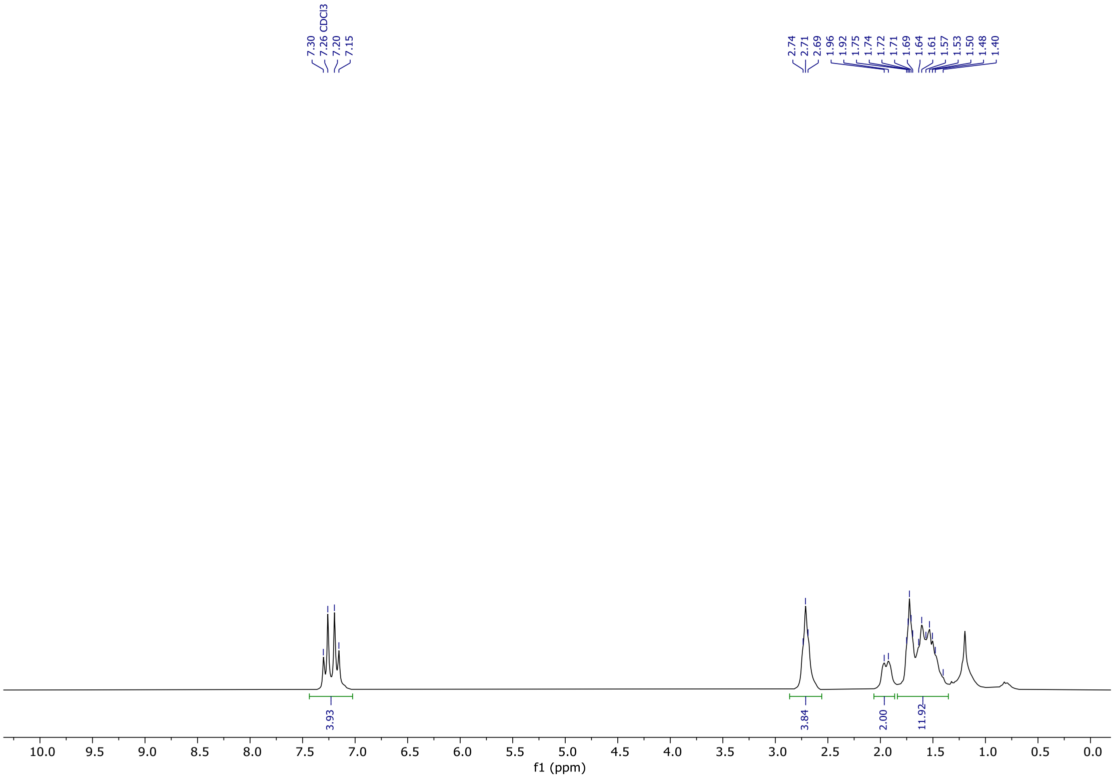

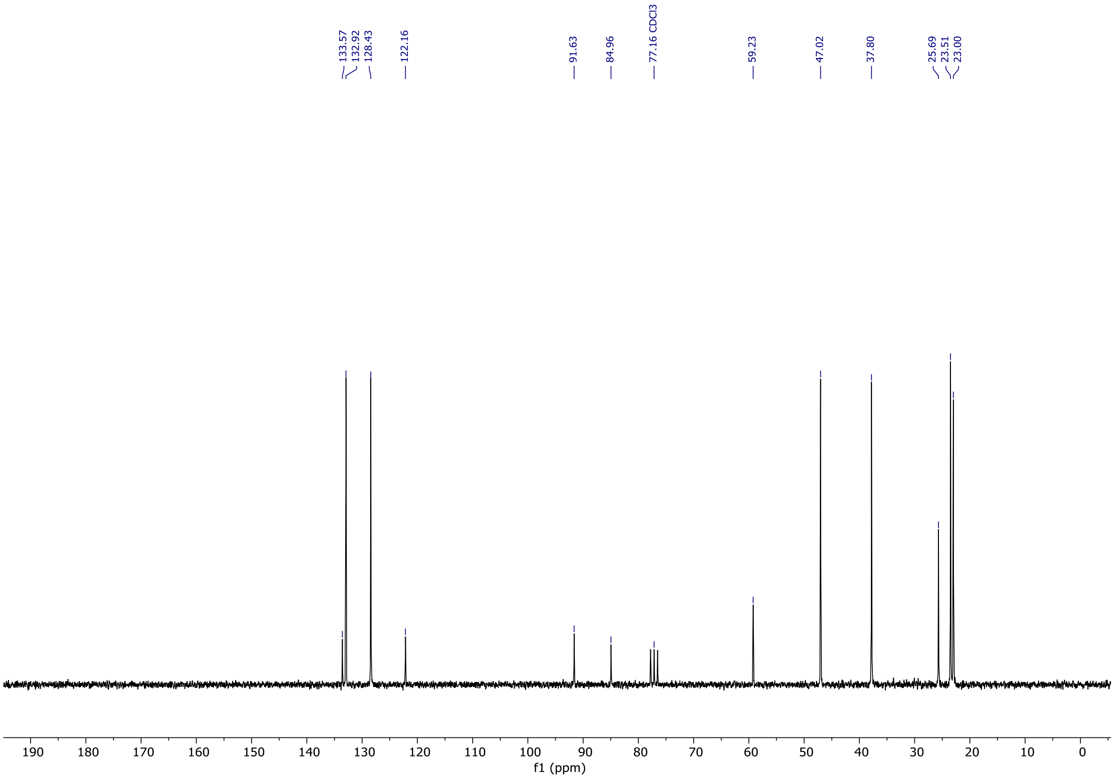

Figure S8: ^{1}H (200 MHz) and ^{13}C (50 MHz) in $CDCl_3$ for **4h**.

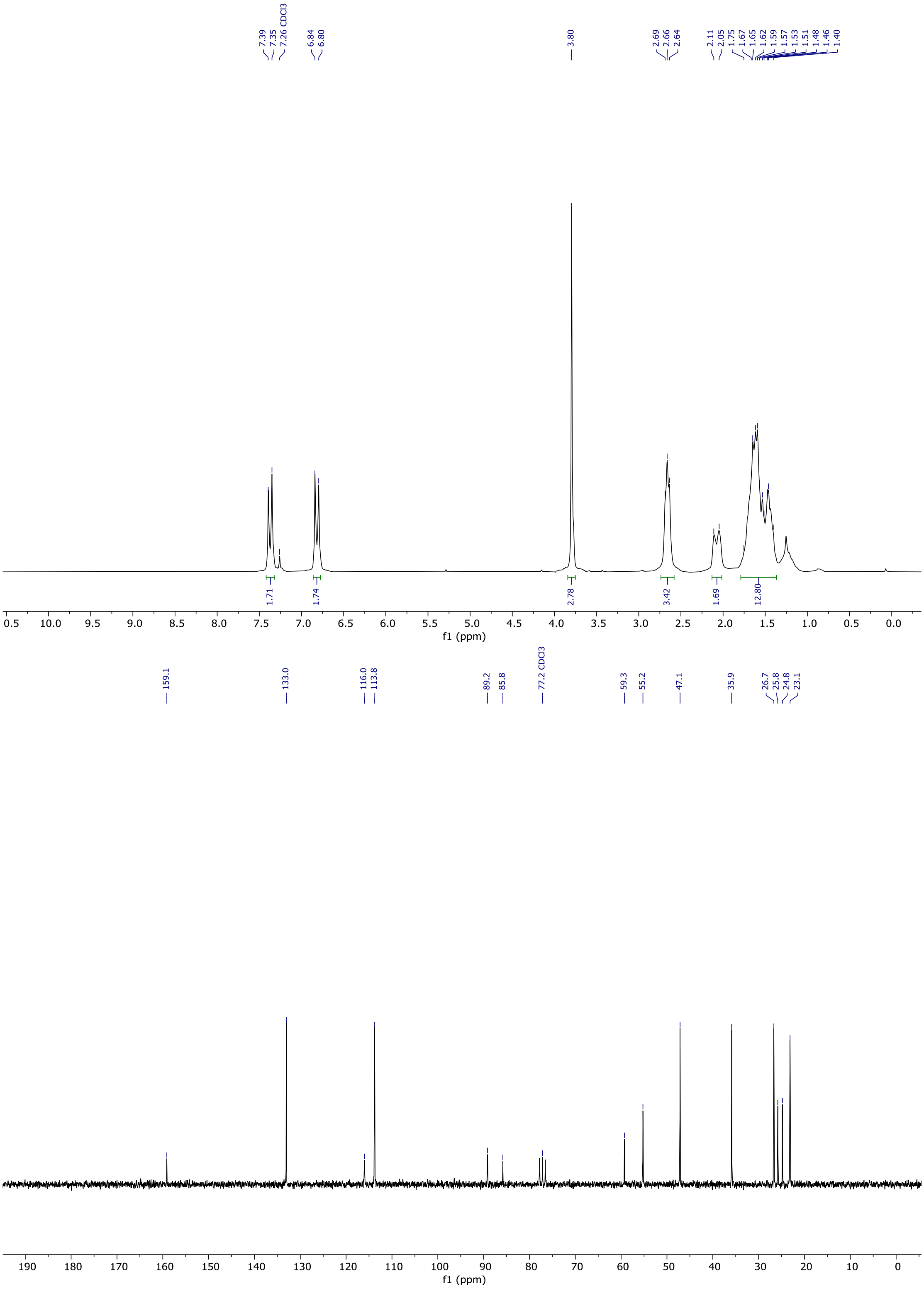

Figure S9: ^{1}H (200 MHz) and ^{13}C (50 MHz) in $CDCl_3$ for **4i**.

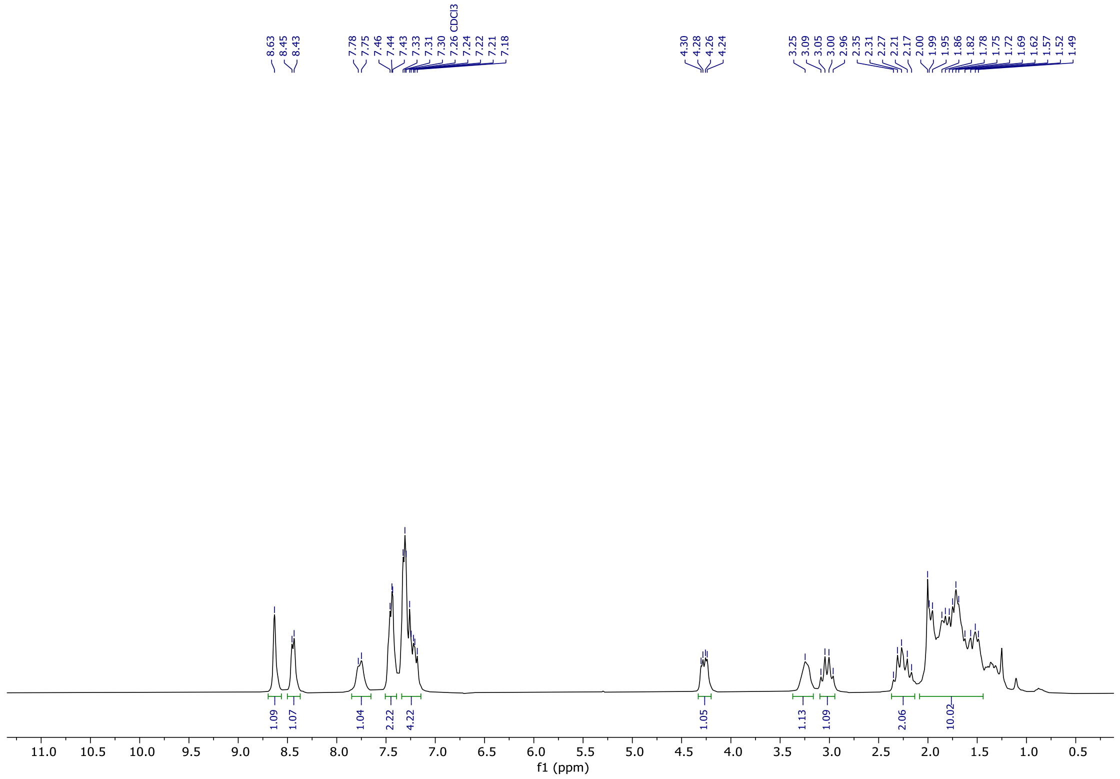

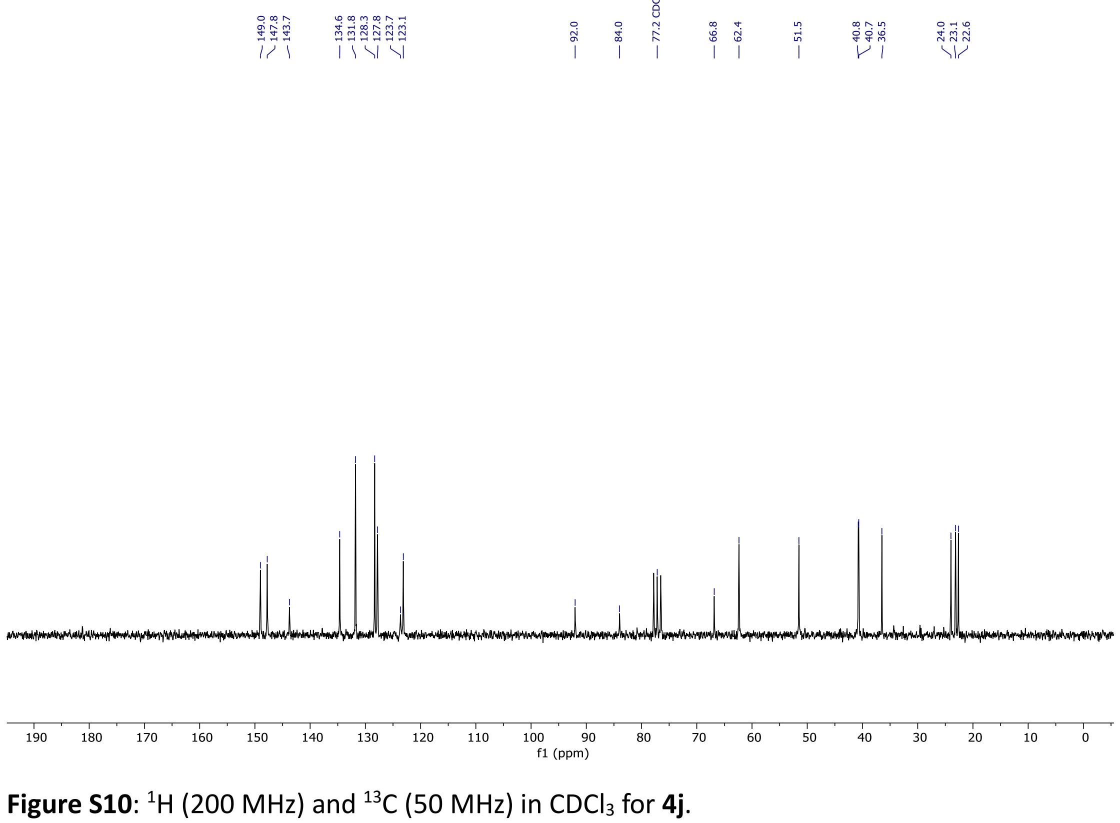

Figure S10: ^{1}H (200 MHz) and ^{13}C (50 MHz) in $CDCl_3$ for **4j**.

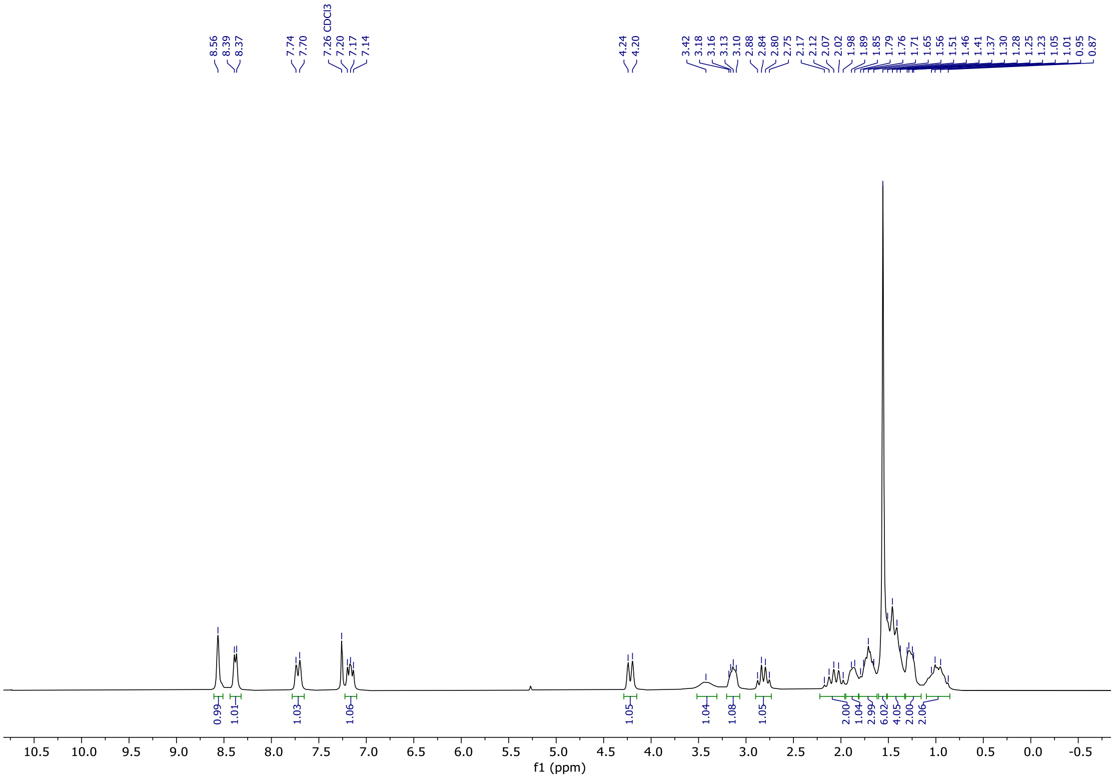

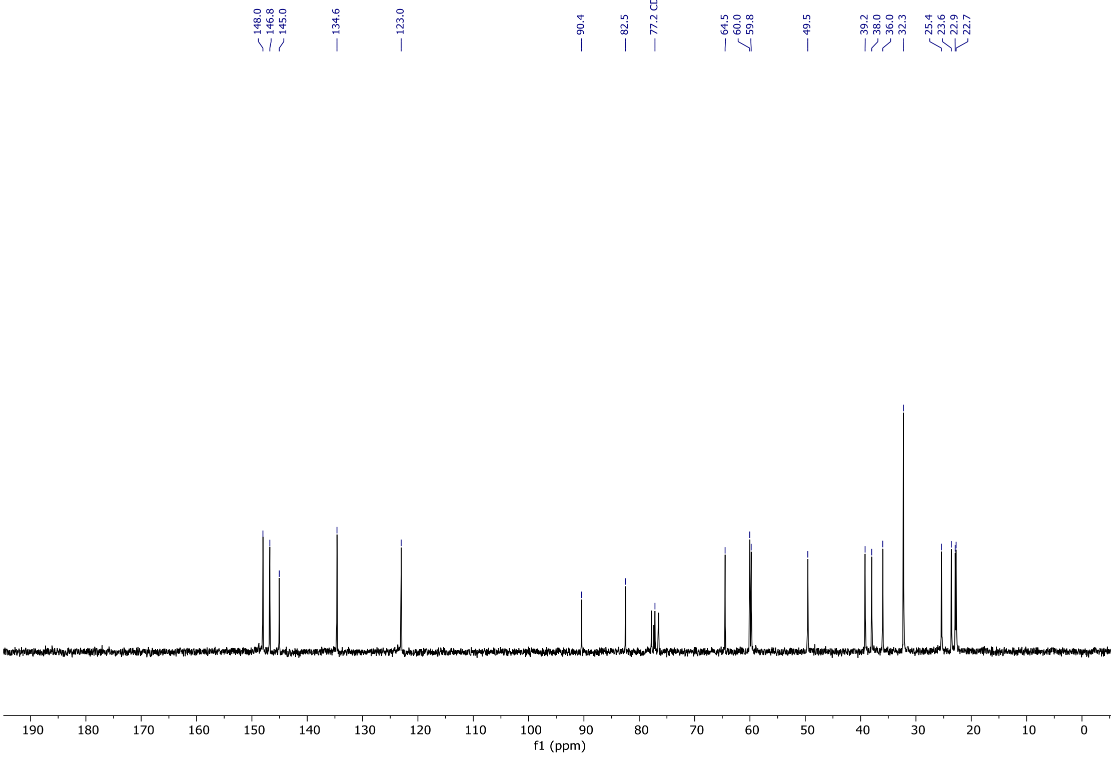

Figure S11: ^{1}H (200 MHz) and ^{13}C (50 MHz) in $CDCl_3$ for **4k**.

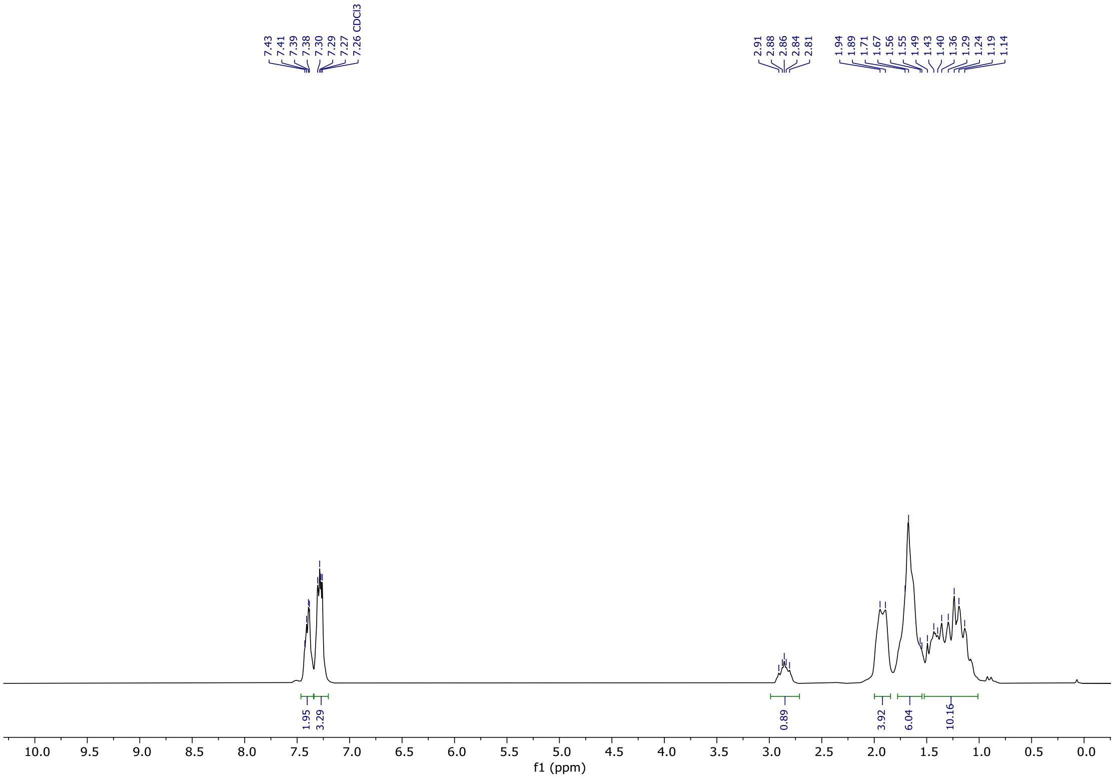

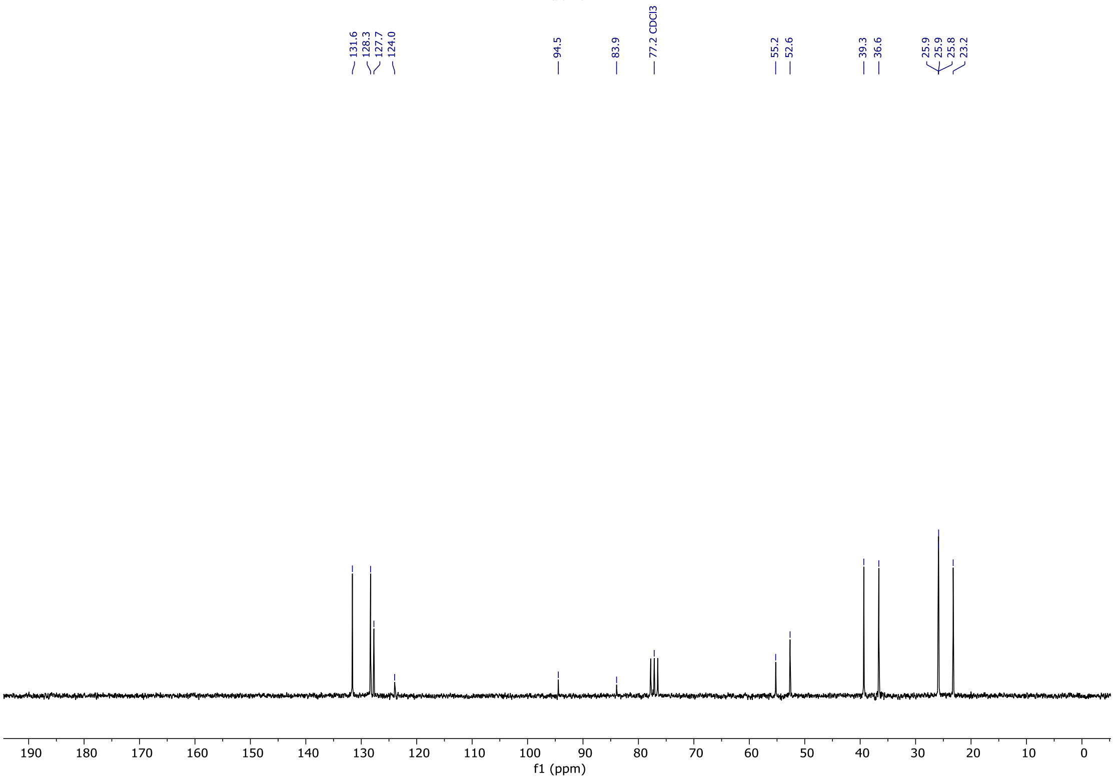

Figure S12: ^{1}H (200 MHz) and ^{13}C (50 MHz) in $CDCl_3$ for **4l**.

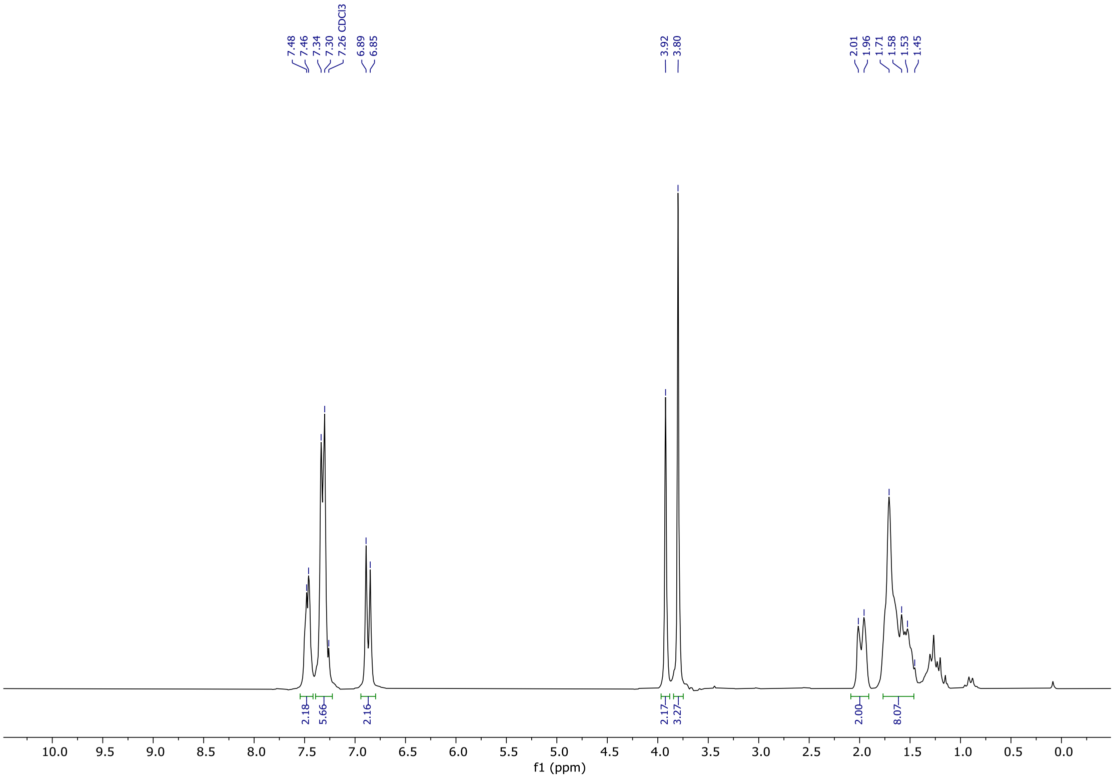

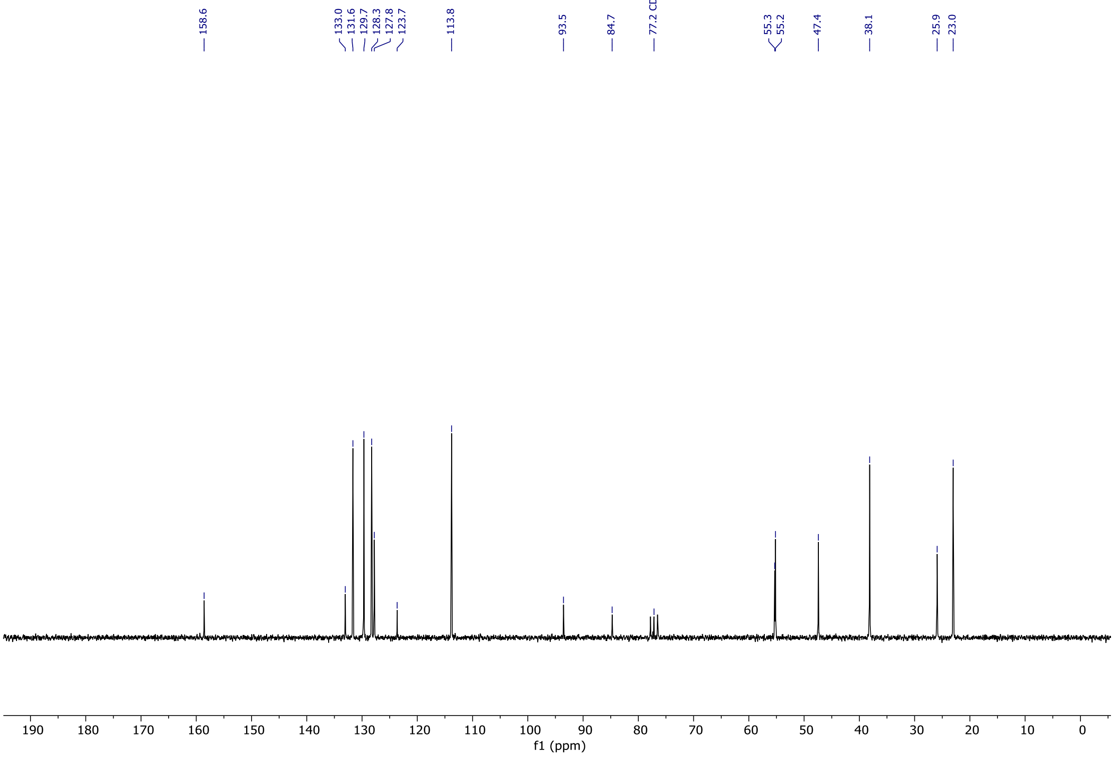

Figure S13: ^{1}H (400 MHz) and ^{13}C (50 MHz) in $CDCl_3$ for **4m**.

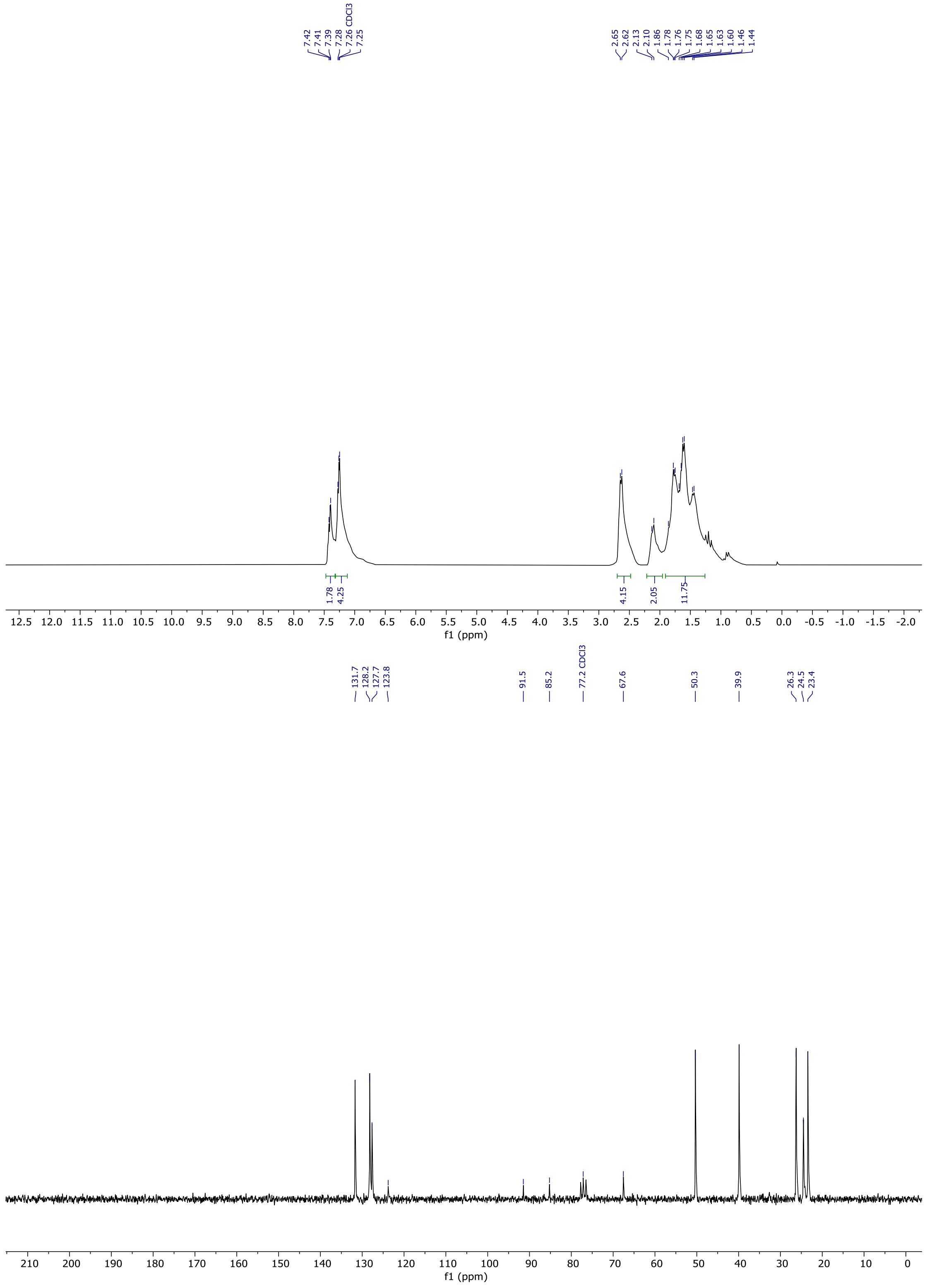

Figure S14: ^{1}H (200 MHz) and ^{13}C (50 MHz) in $CDCl_3$ for **4n**.

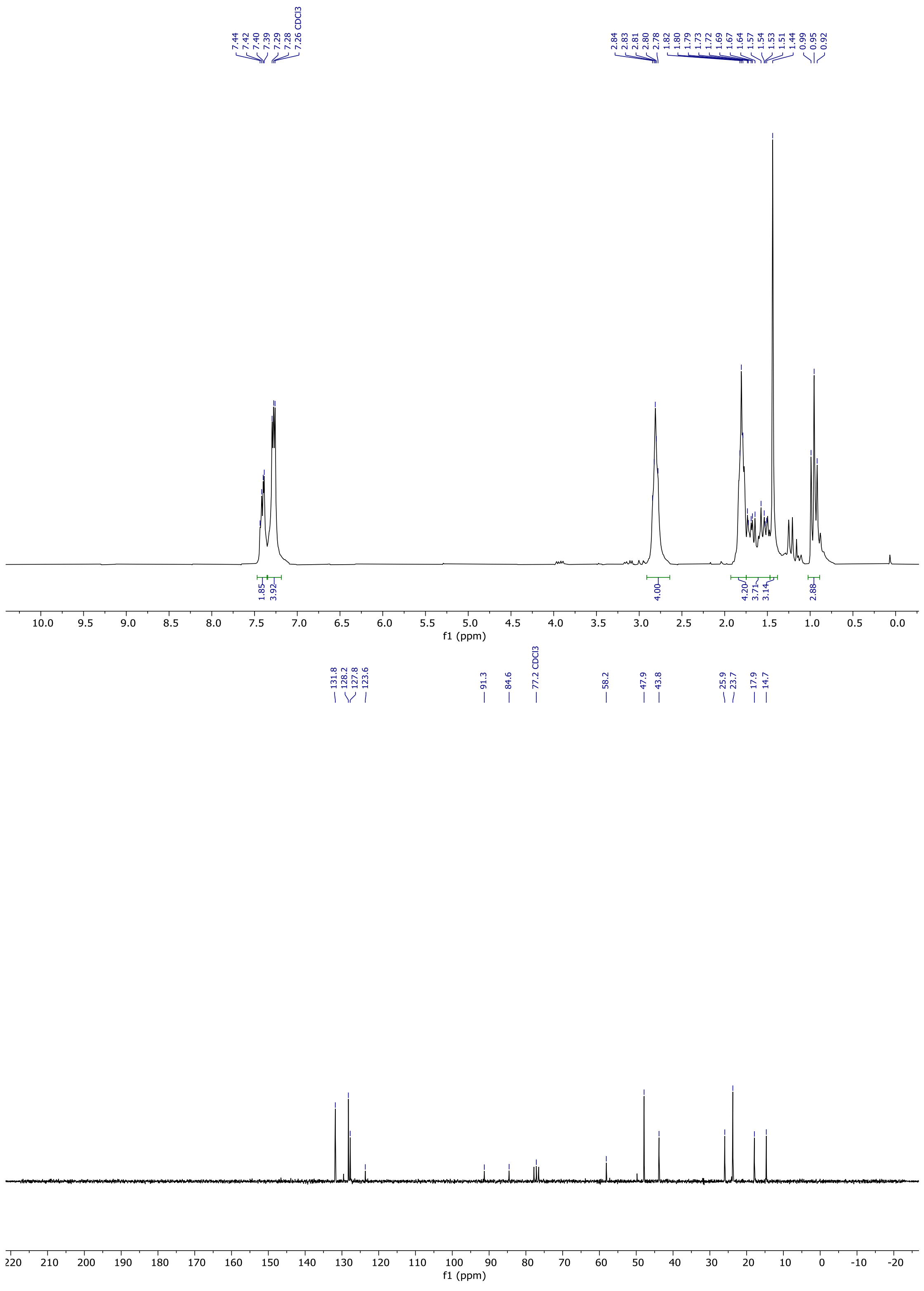

Figure S15: ^{1}H (200 MHz) and ^{13}C (50 MHz) in $CDCl_3$ for **4p**.

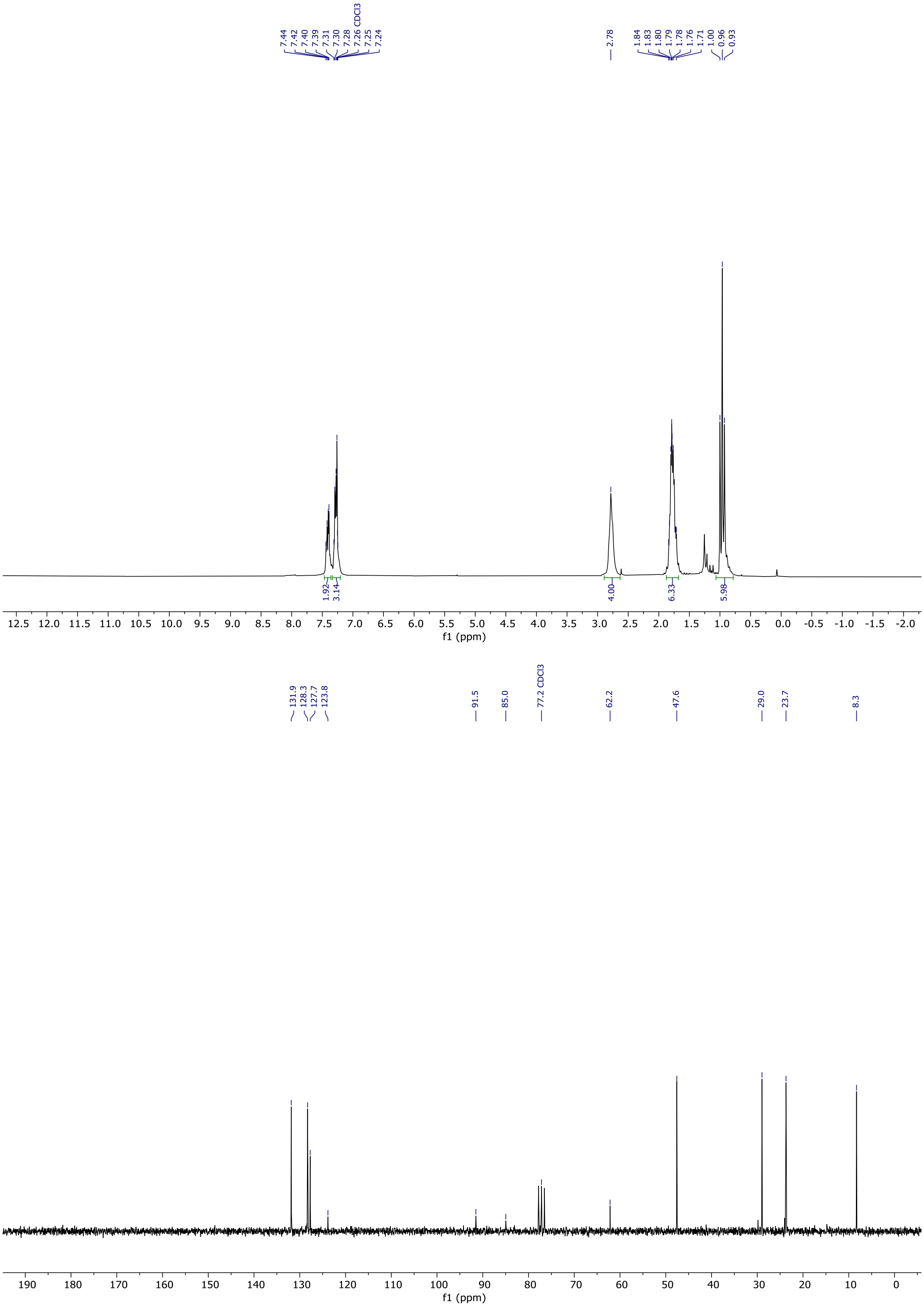

Figure S16: ^{1}H (200 MHz) and ^{13}C (50 MHz) in $CDCl_3$ for **4q**.

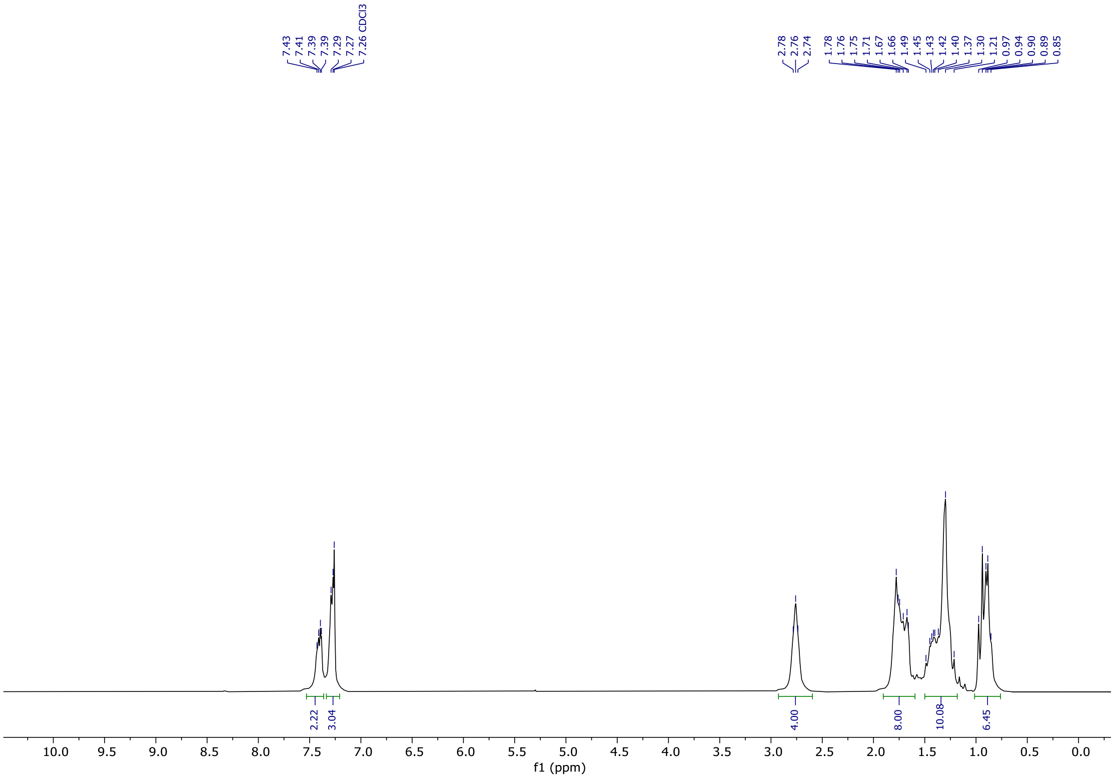

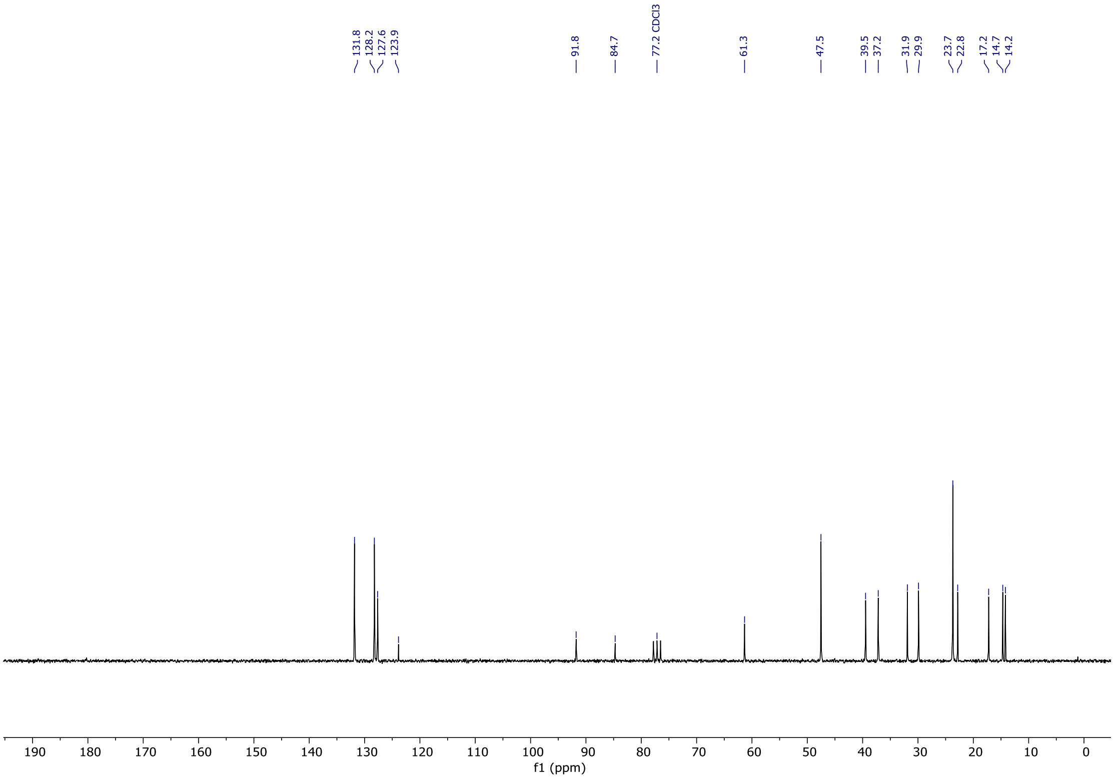

Figure S17: ^{1}H (400 MHz) and ^{13}C (50 MHz) in $CDCl_3$ for **4r**.

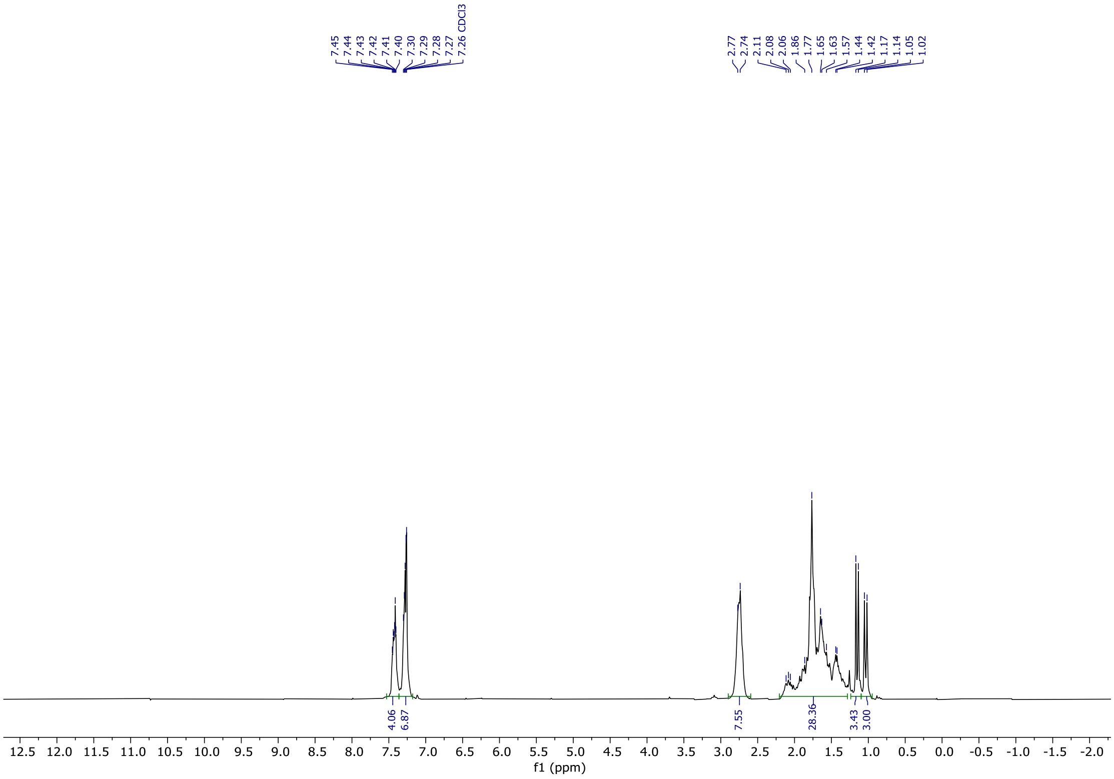

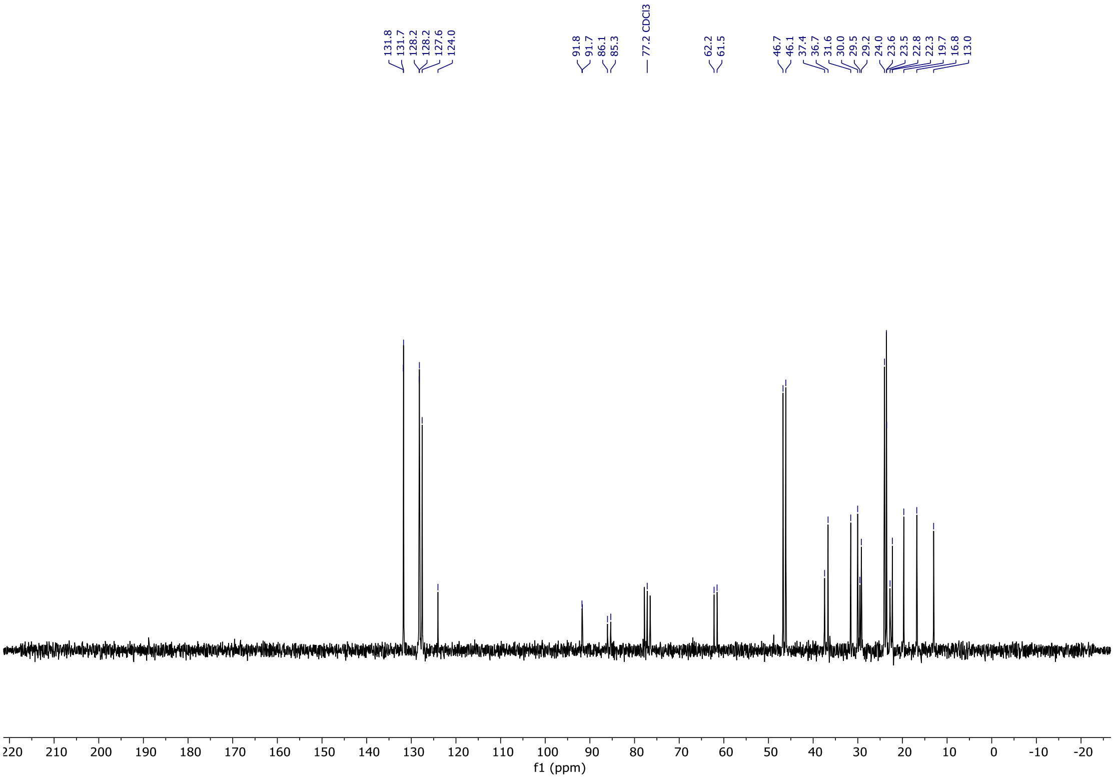

Figure S18: ^{1}H (200 MHz) and ^{13}C (50 MHz) in $CDCl_3$ for **4s**.

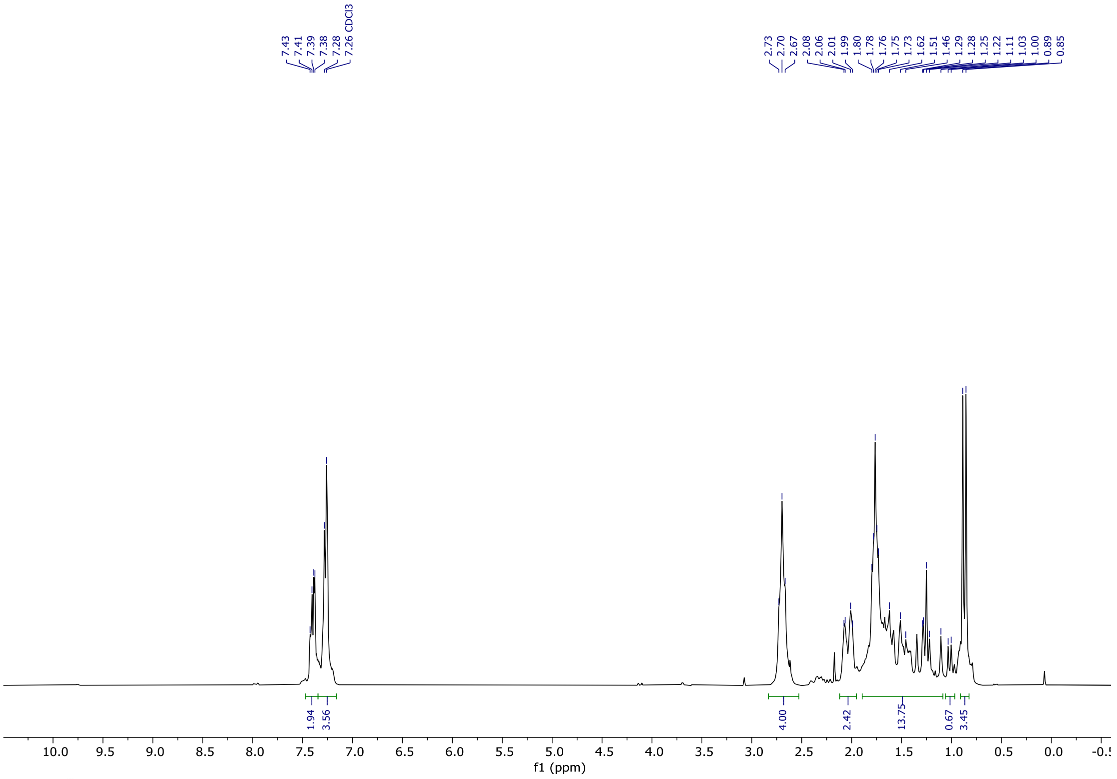

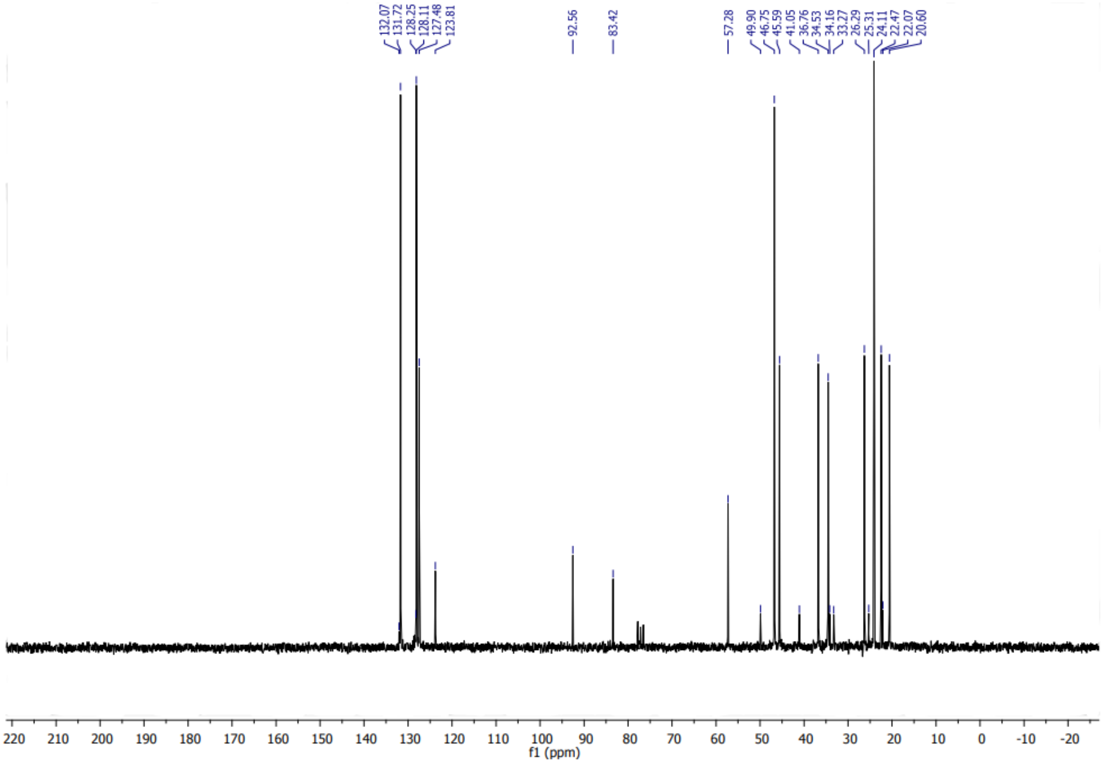

Figure S19: ^{1}H (200 MHz) and ^{13}C (50 MHz) in $CDCl_3$ for **4t**.

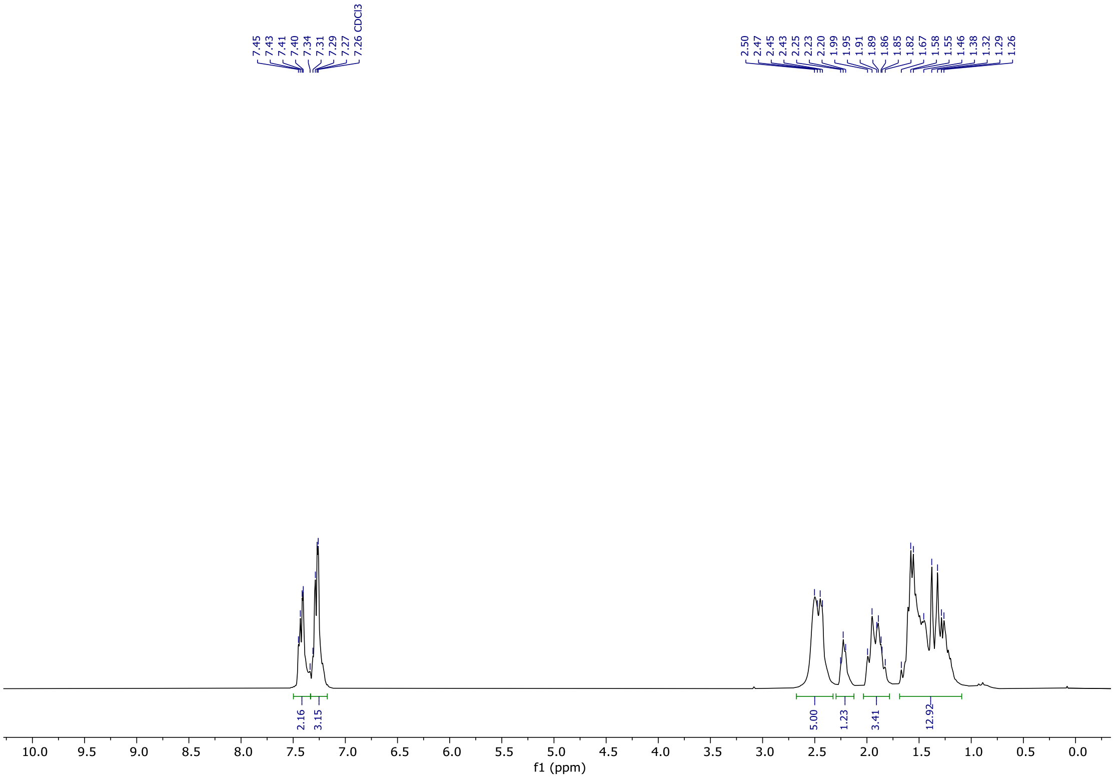

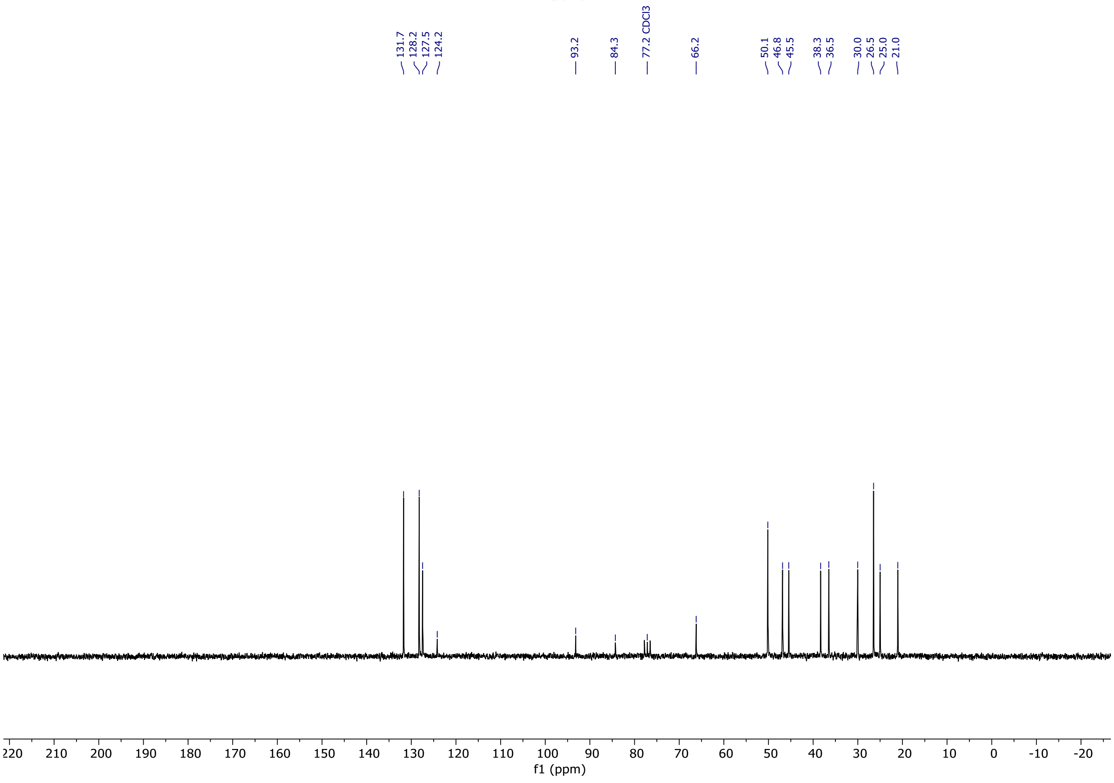

Figure S20: ^{1}H (200 MHz) and ^{13}C (50 MHz) in $CDCl_3$ for **4u**.

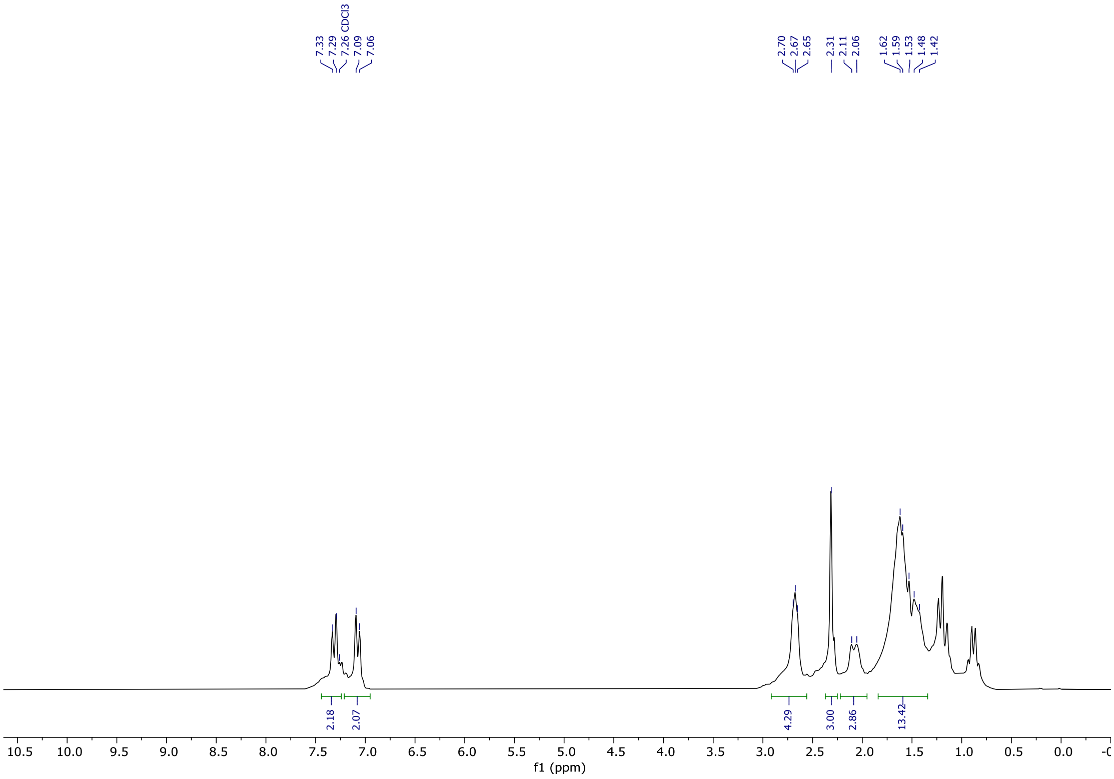

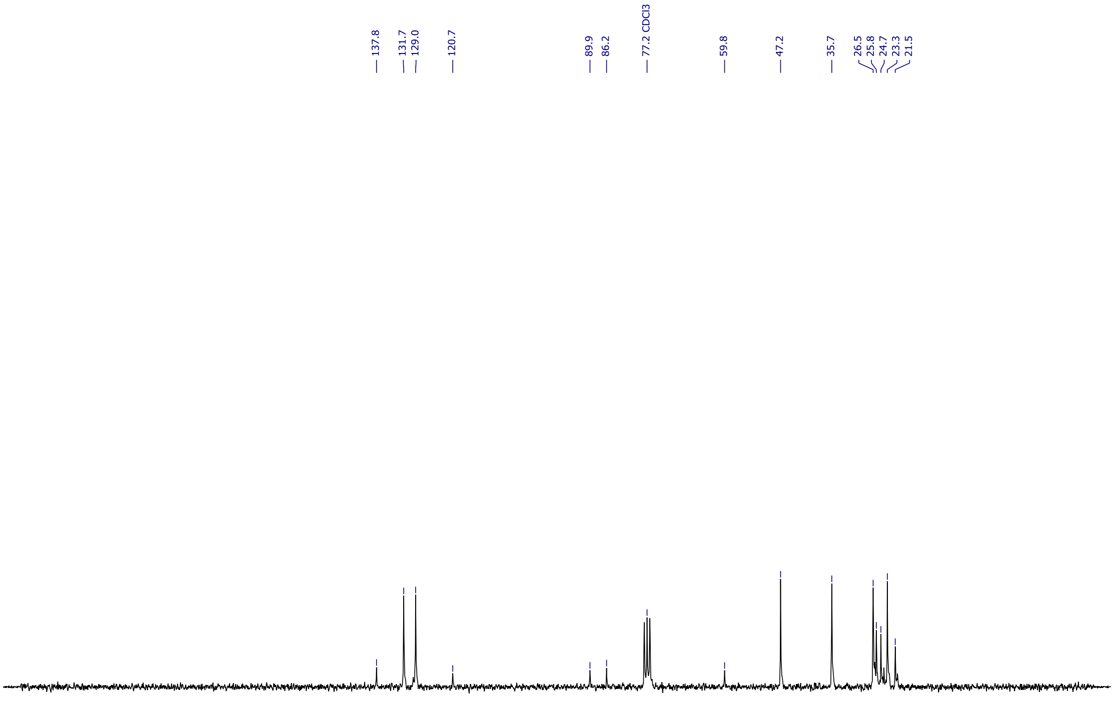

Figure S21: ^{1}H (200 MHz) and ^{13}C (50 MHz) in $CDCl_3$ for **4v**.

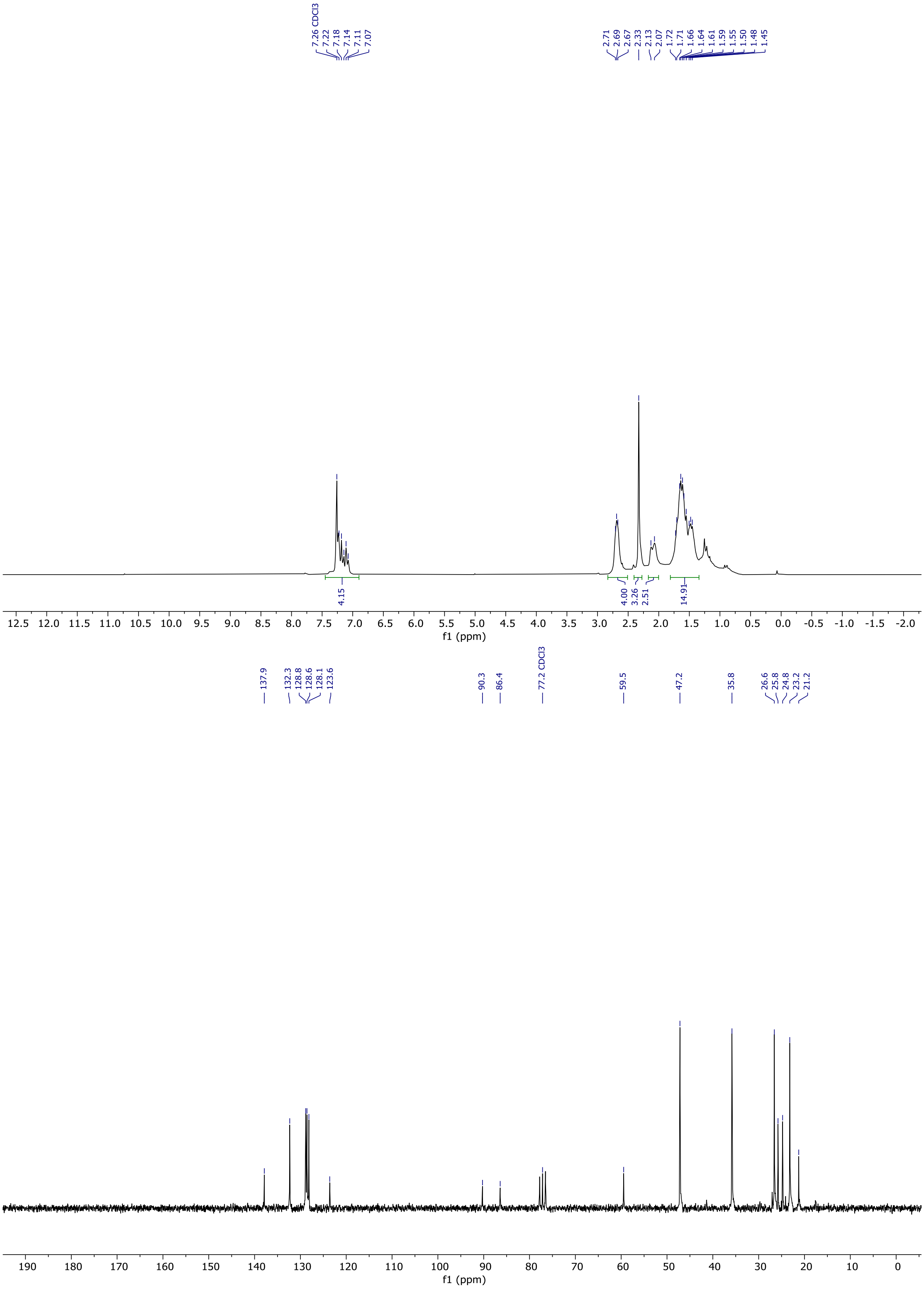

Figure S22: ^{1}H (200 MHz) and ^{13}C (50 MHz) in $CDCl_3$ for **4w**.

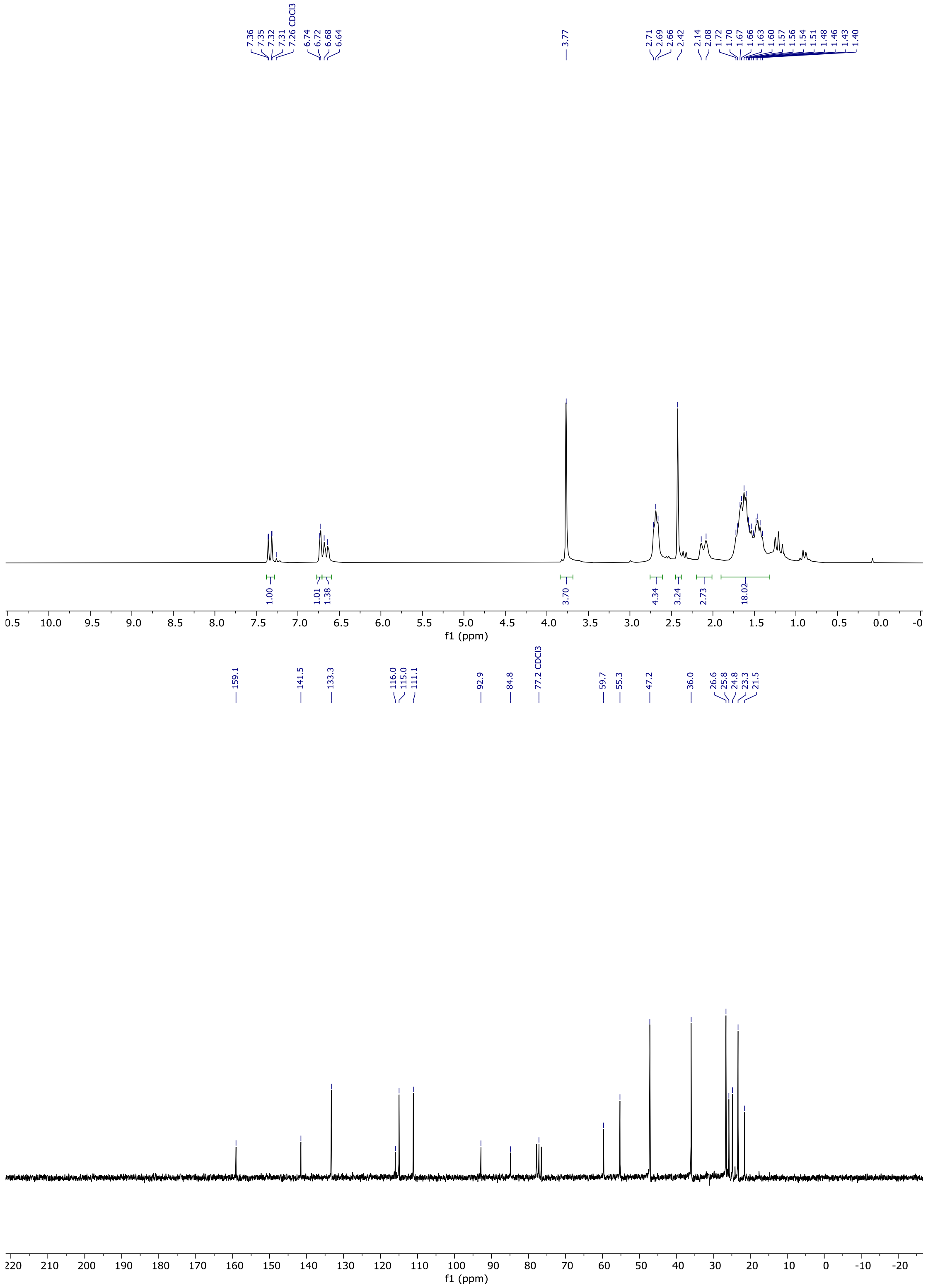

Figure S23: ^{1}H (200 MHz) and ^{13}C (50 MHz) in $CDCl_3$ for **4x**.

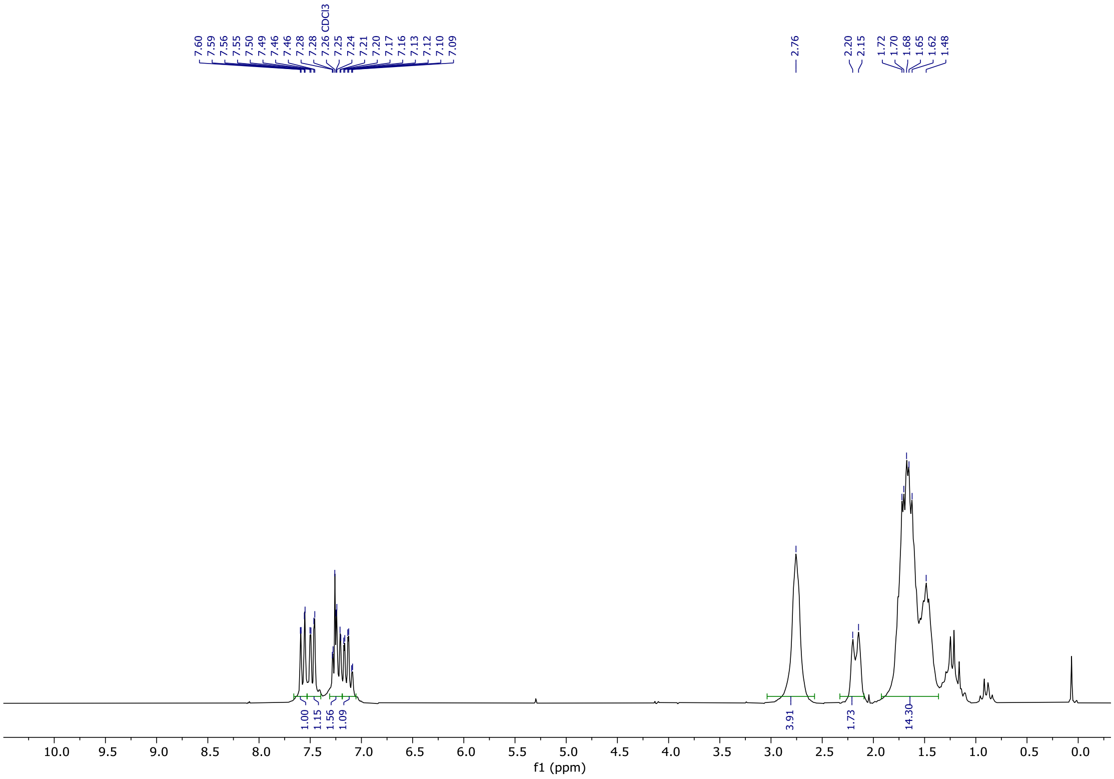

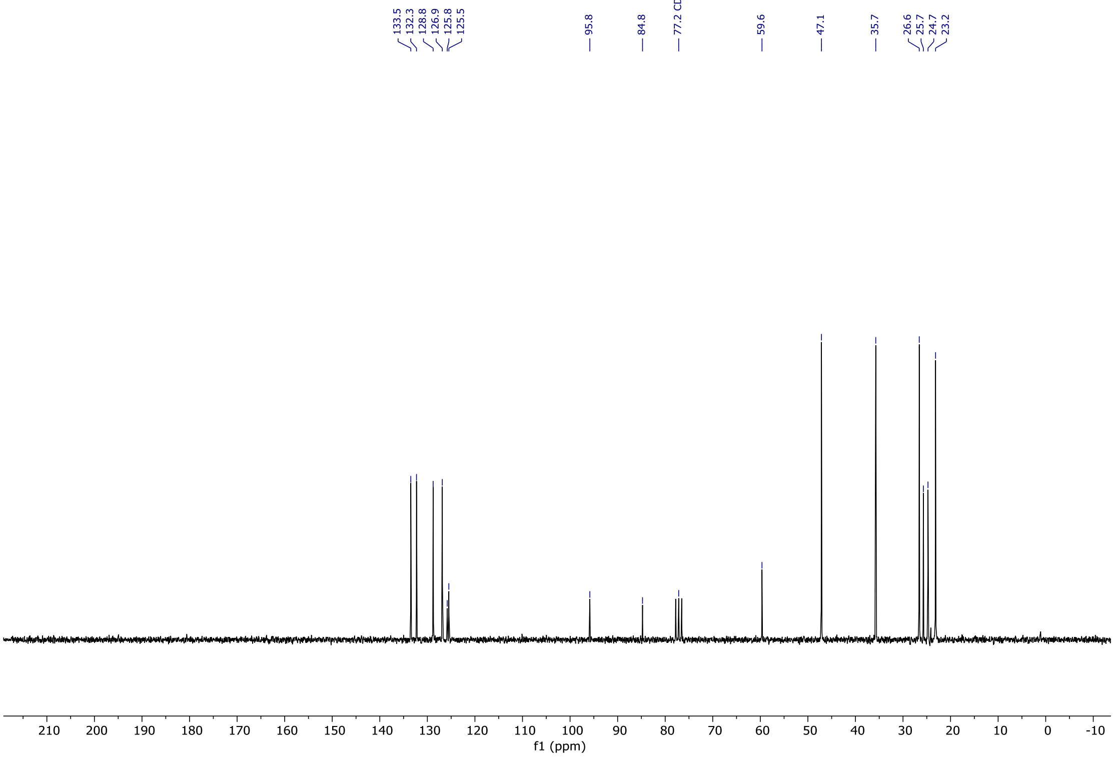

Figure S24: ^{1}H (200 MHz) and ^{13}C (50 MHz) in $CDCl_3$ for **4y**.

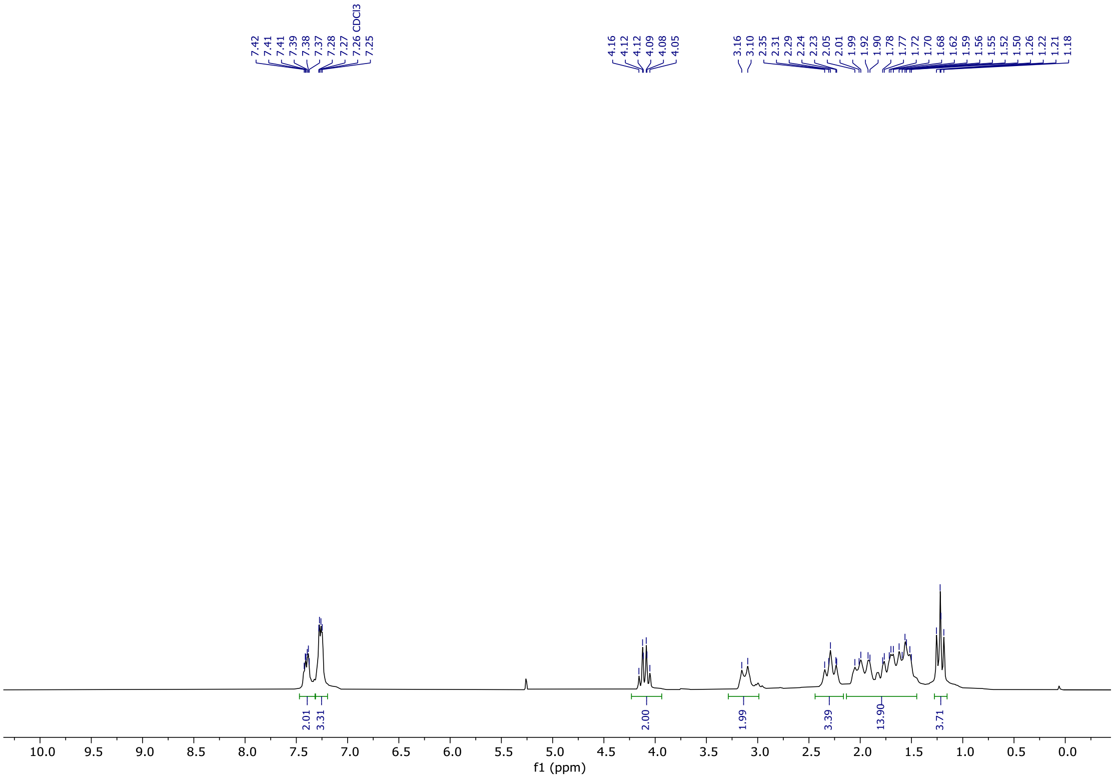

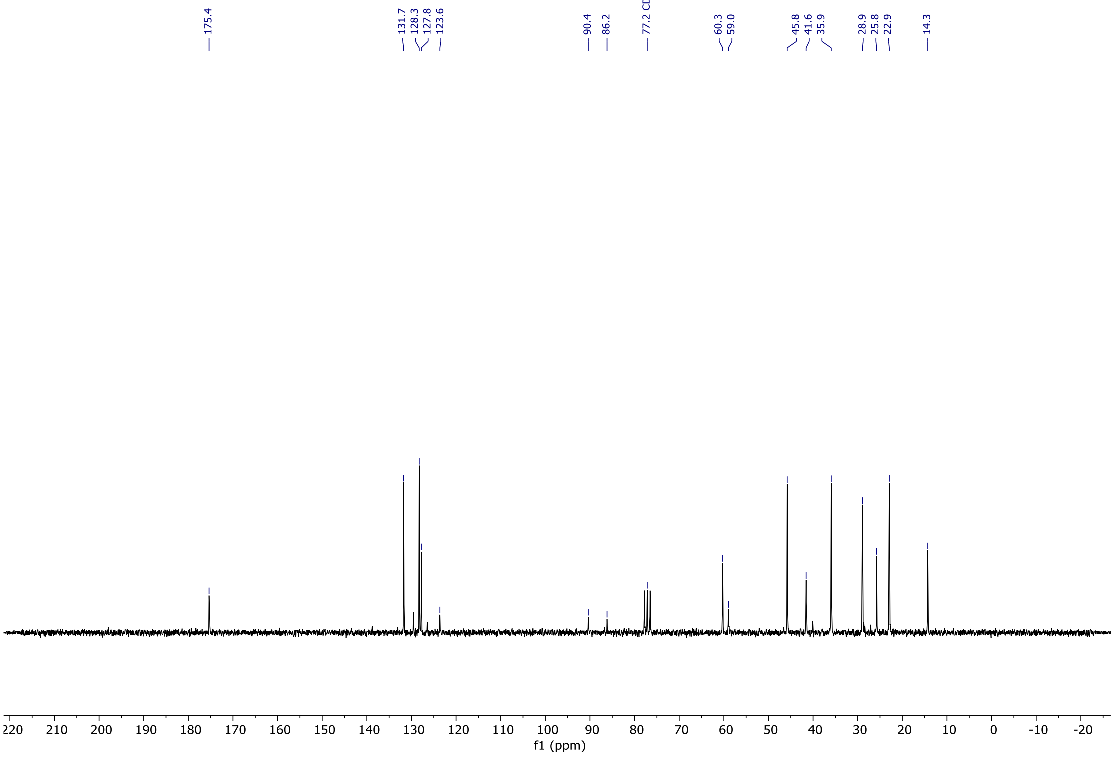

Figure S25: ^{1}H (200 MHz) and ^{13}C (50 MHz) in $CDCl_3$ for **4z**.

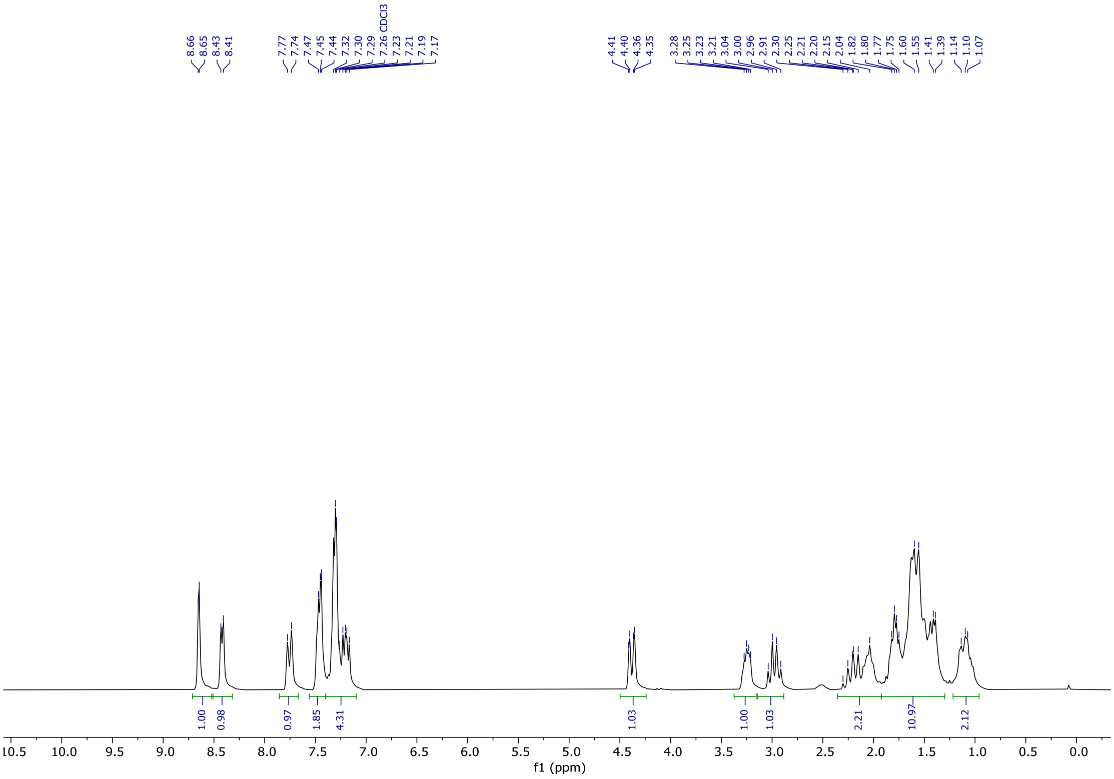

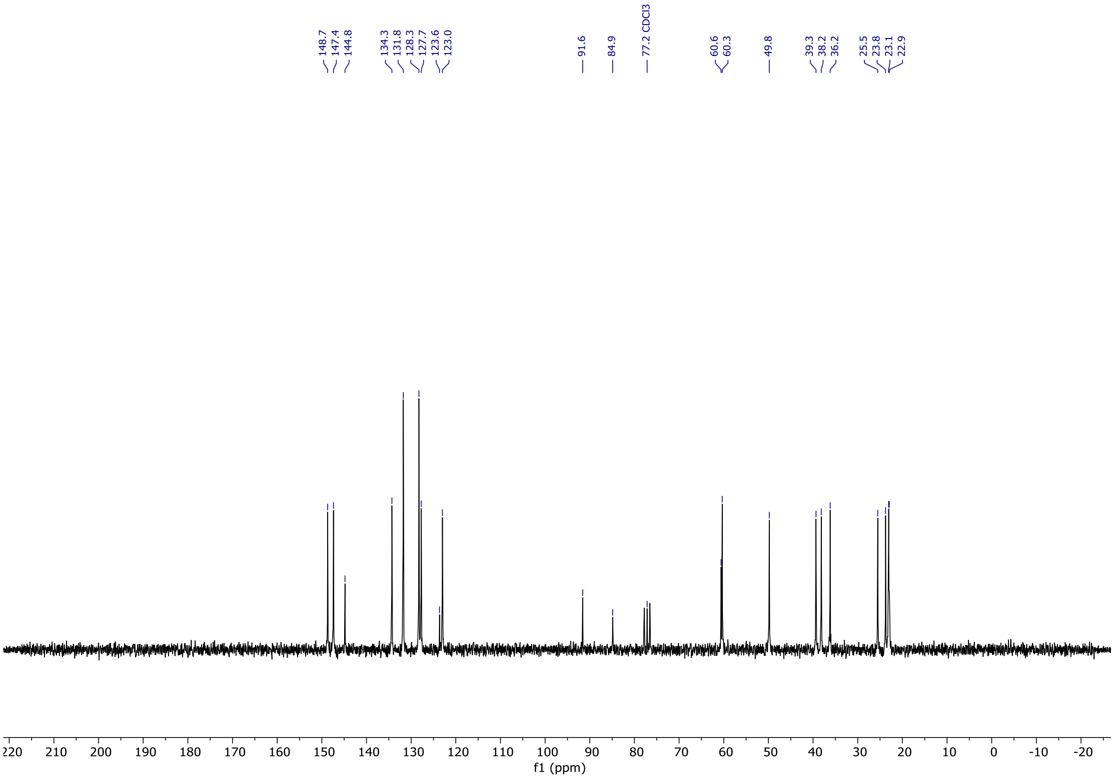

Figure S26: ^{1}H (200 MHz) and ^{13}C (50 MHz) in $CDCl_3$ for **4aa**.

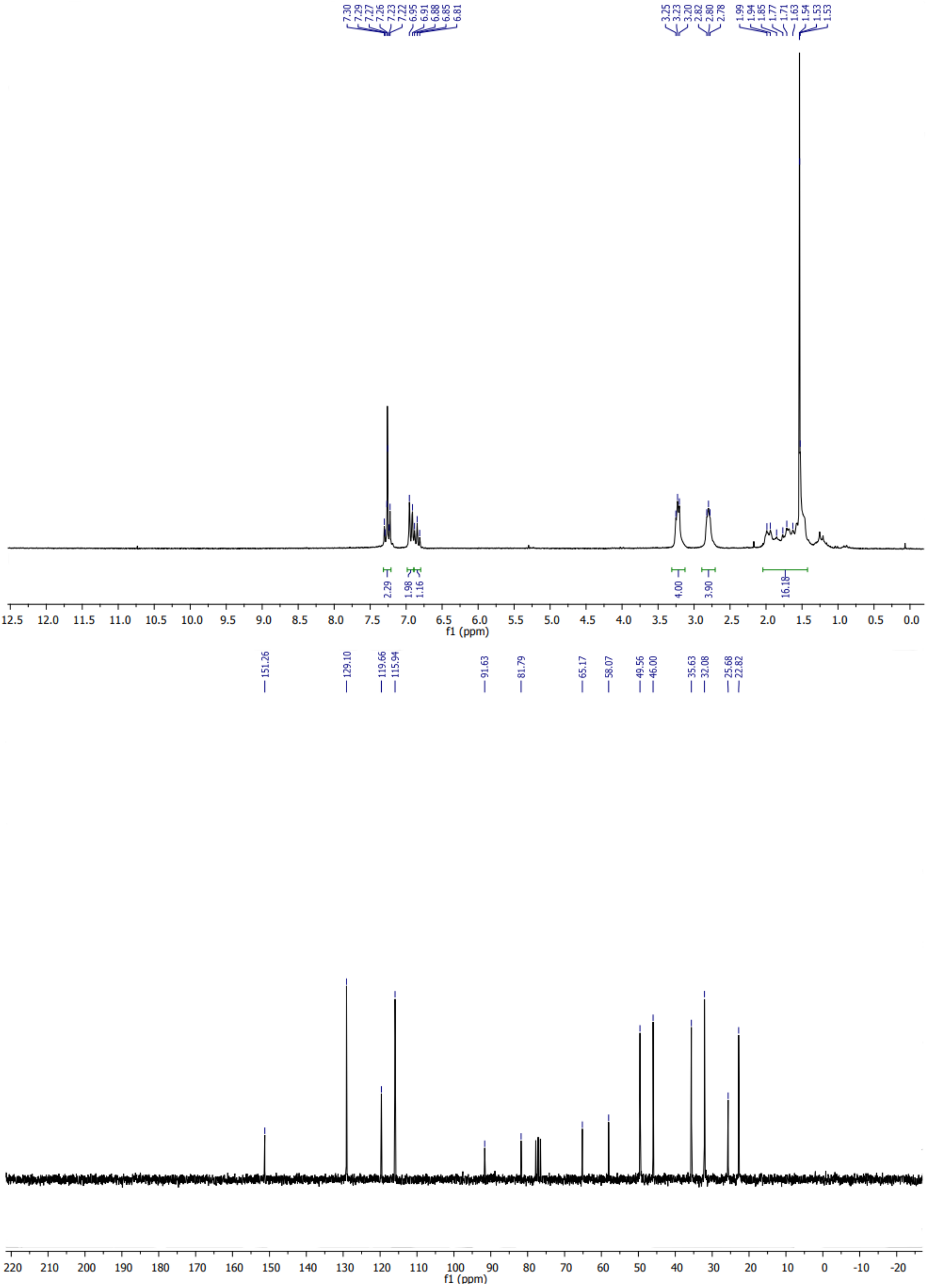

Figure S27: ^{1}H (200 MHz) and ^{13}C (50 MHz) in $CDCl_3$ for **4ab**.

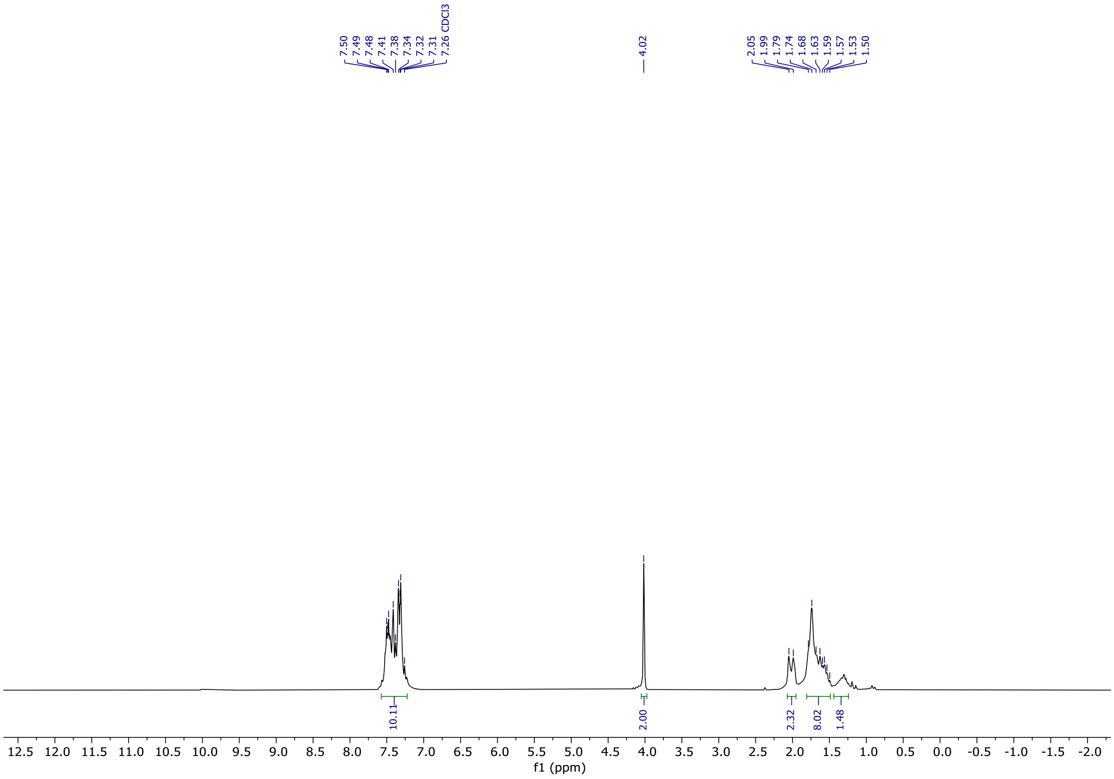

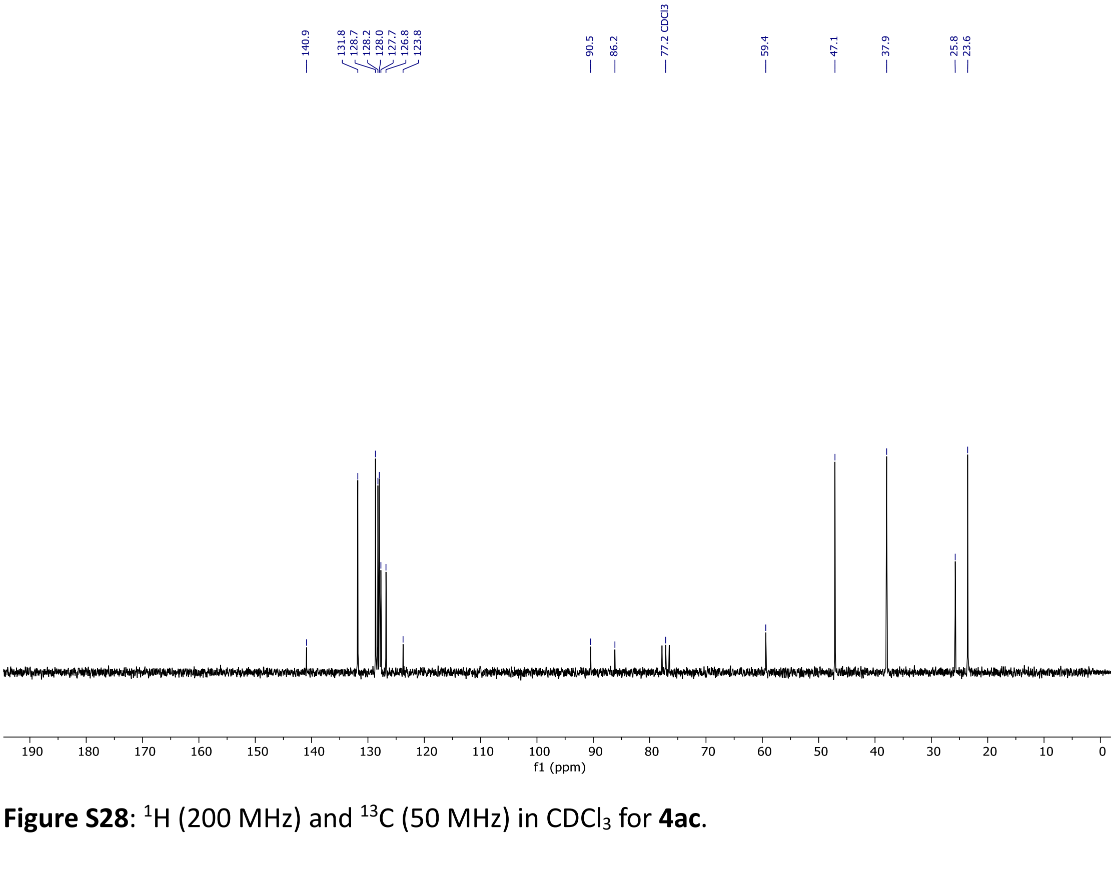

Figure S28: ^{1}H (200 MHz) and ^{13}C (50 MHz) in $CDCl_3$ for **4ac**.

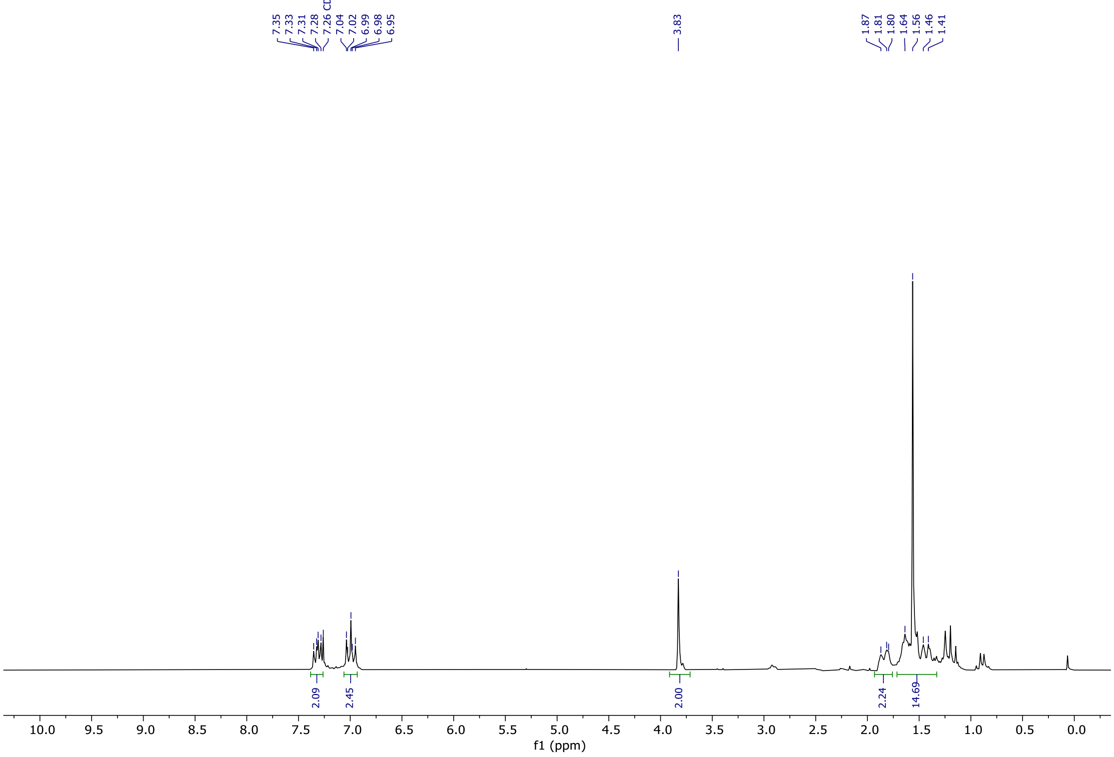
7.35
7.33
7.31
7.28
7.26 CDCl3
7.04
7.02
6.99
6.98
6.95
3.83
1.87
1.81
1.80
1.64
1.56
1.46
1.41
2.09
2.45
2.00
2.24
14.69
10.0 9.5 9.0 8.5 8.0 7.5 7.0 6.5 6.0 5.5 5.0 4.5 4.0 3.5 3.0 2.5 2.0 1.5 1.0 0.5 0.0
f1 (ppm)

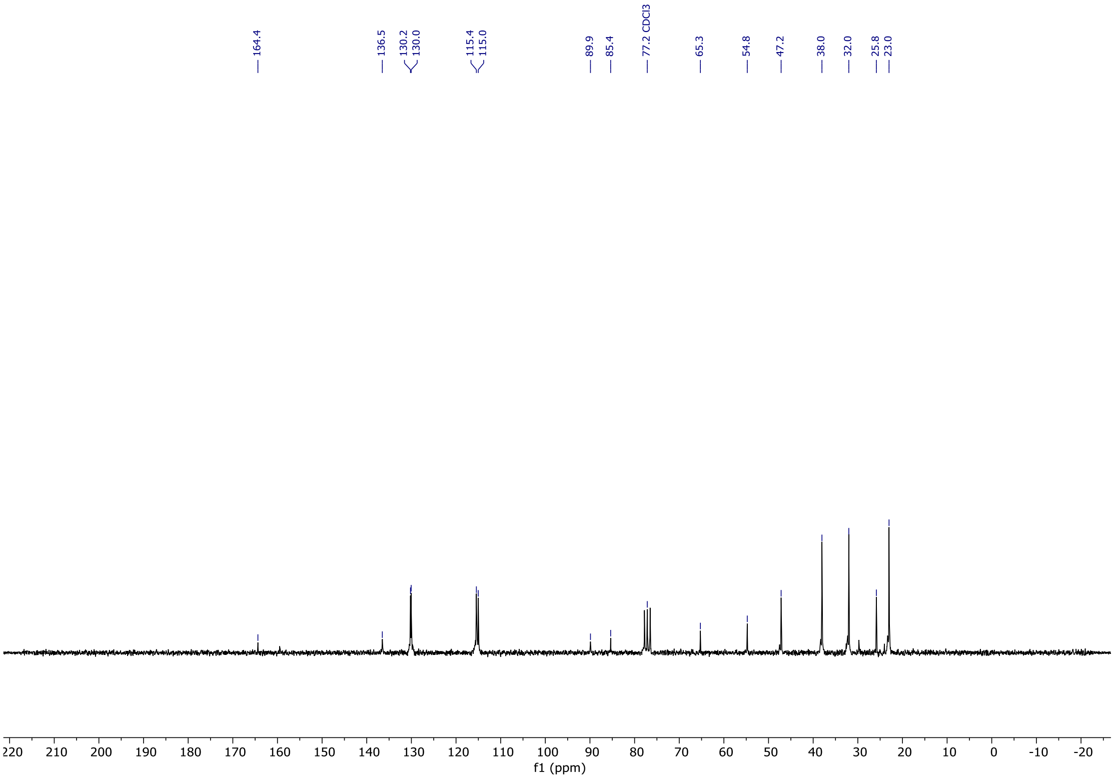
164.4
136.5
130.2
130.0
115.4
115.0
89.9
85.4
77.2 CDCl3
65.3
54.8
47.2
38.0
32.0
25.8
23.0
220 210 200 190 180 170 160 150 140 130 120 110 100 90 80 70 60 50 40 30 20 10 0 -10 -20
f1 (ppm)

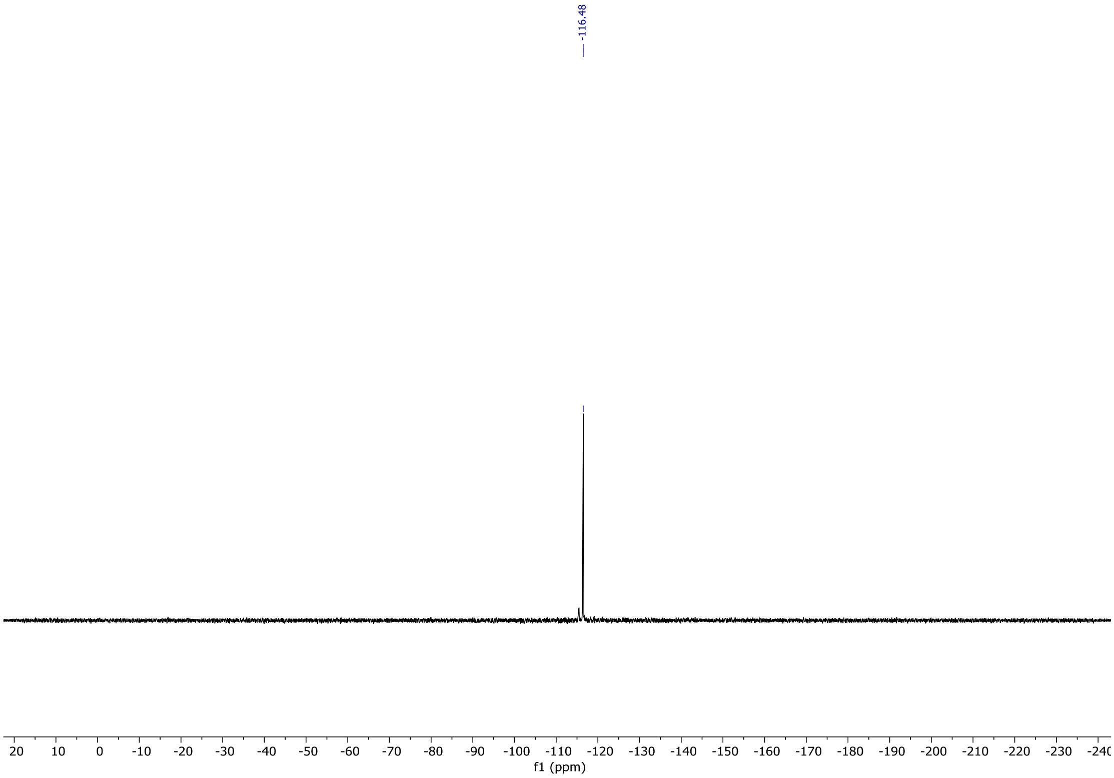

Figure S29: ^{1}H (200 MHz), ^{13}C (50 MHz) and ^{19}F (188 MHz) in $CDCl_3$ for **4ad**.

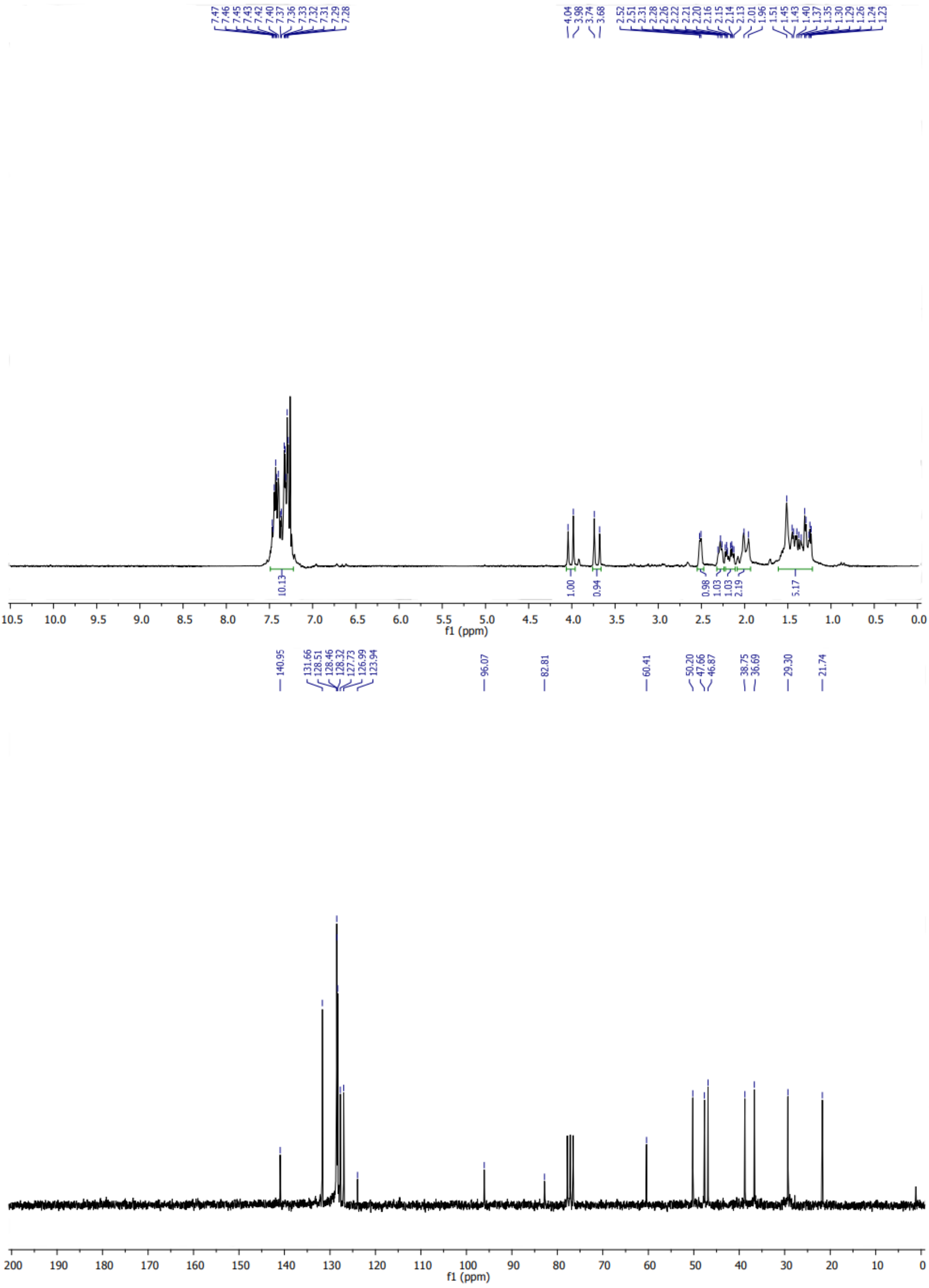

Figure S30: ^{1}H (200 MHz) and ^{13}C (50 MHz) in $CDCl_3$ for **4ae**.
